# High-pressure reversibility in a plastically flexible coordination polymer crystal

**DOI:** 10.1038/s41467-021-24165-x

**Published:** 2021-06-23

**Authors:** Xiaojiao Liu, Adam A. L. Michalchuk, Biswajit Bhattacharya, Nobuhiro Yasuda, Franziska Emmerling, Colin R. Pulham

**Affiliations:** 1 EaStChem School of Chemistry and Centre for Science at Extreme Conditions (CSEC), University of Edinburgh, Edinburgh, UK; 2 Federal Institute for Materials Research and Testing (BAM), Berlin, Germany; 3 Japan Synchrotron Radiation Research Institute (JASRI), Hyogo, Japan

**Keywords:** Mechanical properties, Crystal engineering, Computational chemistry

## Abstract

Single crystals which exhibit mechanical flexibility are promising materials for advanced technological applications. Before such materials can be used, a detailed understanding of the mechanisms of bending is needed. Using single crystal X-ray diffraction and microfocus Raman spectroscopy, we study in atomic detail the high-pressure response of the plastically flexible coordination polymer [Zn(μ-Cl)_2_(3,5-dichloropyridine)_2_]_*n*_ (**1**). Contradictory to three-point bending, quasi-hydrostatic compression of (**1**) is completely reversible, even following compression to over 9 GPa. A structural phase transition is observed at *ca*. 5 GPa. DFT calculations show this transition to result from the pressure-induced softening of low-frequency vibrations. This phase transition is not observed during three-point-bending. Microfocus synchrotron X-ray diffraction revealed that bending yields significant mosaicity, as opposed to compression. Hence, our studies indicate of overall disparate mechanical responses of bulk flexibility and quasi-hydrostatic compression within the same crystal lattice. We suspect this to be a general feature of plastically bendable materials.

## Abstract (web summary)

Mechanically flexible single crystals are promising materials for advanced technological applications. Here, the authors study the high pressure response of a plastically flexible coordination polymer and provide indication of an overall disparate mechanical response of bulk flexibility and quasi-hydrostatic compression within the same crystal lattice.

## Introduction

The mechanical flexibility exhibited by some single crystals of organic molecules and coordination polymers (CP) has attracted widespread attention^1–4^. Examples are now known to include both elastically (fully reversible) and plastically (irreversible) bendable materials. These materials offer exceptional opportunities to develop next-generation advanced materials. Applications are already reported for optical waveguide technologies^5^, as tunable magnets^6^, and as flexible electronic conductors^7,8^. In some cases, useful functional properties*—*such as fluorescence output*—*are known to vary with the state of bending^9,10^. Such effects offer significant potential for sensing and display applications.

Models have been proposed to explain the mechanical flexibility of molecular and metal-organic crystals^11–13^. Conceptually, these models are derived from analysis of the intermolecular interactions that are present in the solid state. Plastically flexible crystals typically comprise low-energy shear planes, whereas elastically flexible materials often contain relatively weak isotropic intermolecular interactions. To date, these models have proved highly successful for the design of new materials. However, an increasing number of mechanically flexible materials are being reported which do not adhere to existing design principles^8,14,15^. Notable exceptions include the recently discovered class of flexible single-crystal CPs^3,16^. The mechanisms of flexible materials must be better understood before such materials can be selectively designed for targeted, practical applications as advanced functional materials.

The need for further crystallographic detail was recently highlighted in the pioneering work of Reddy and Naumov^17^. Using microfocus synchrotron X-ray diffraction, deformation of the crystallographic unit cell was measured directly within the deformed region of a bent single crystal. Atomic positions have been since measured in similar experiments, providing unrivalled detail of mechanical flexibility^14,18,19^. Importantly, X-ray diffraction offers only an averaged picture of the irradiated material; the inhomogeneity of distortion during bending remains an exceptional challenge^20^. Only a single study has so far attempted to capture this inhomogeneous field by exploring modulated crystallographic distortions in aminoboranes^18^. Hence, despite impressive efforts, direct approaches to extract atomistic detail of mechanically bent single crystals remain a significant challenge.

It is instead convenient to explore atomistic deformations during mechanical bending indirectly, through models. Early studies proposed that the structural distortions associated with mechanical bending should follow the ‘intrinsic’ thermal motion of atoms in the crystal lattice^21,22^. This notion was recently refuted for the case of an elastically flexible molecular material, Cu(II) acetylacetonate^23^, although its validity across a wider array of flexible crystals has not yet been explored. Alternatively, it is widely suggested that mechanical bending results from local compressive and tensile distortions, a hypothesis supported also by the recent microfocus X-ray diffraction studies^14,18^. Correspondingly, extensive efforts have been devoted to measuring the anisotropic mechanical properties (e.g., Young’s modulus and Poisson ratio) of flexible materials^14,17,19,24^. The mechanical hardness is known to vary across the deformed crystal, hardening at the inner arc^19^. This strongly suggests an increase of local pressure during bending. These findings have motivated us to explore the effects of hydrostatic compression on the structure of mechanically flexible materials.

Hydrostatic compression to high pressure (HP) is an indispensable tool for elucidating the reactivity and behaviour of organic and metal-organic materials^25^. Through HP X-ray diffraction and vibrational spectroscopy, both the structural and dynamical origins of material mechanical properties are accessible. By systematically exploring structural and dynamical changes at pressure, we expect to elucidate the favoured relaxation pathways of flexible materials when exposed to mechanical stress. To the best of our knowledge, no such studies have yet been reported.

**Fig. 1:**
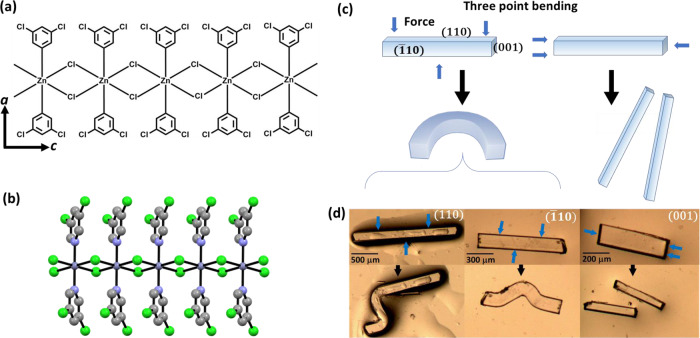
Description of [Zn(μ-Cl)_2_(3,5-dichloropyridine)_2_]_*n*_ (**1**), as reported in our previous work^9^. **a** Schematic representation of the coordination polymer chains of (**1**). **b** Ball and stick model representation of the coordination polymer chain shown in **a**. Atoms are coloured as C- grey; N- blue; Cl- green; Zn – dark blue. **c** Schematic representation of three-point bending. Crystal bending is achieved by immobilising the ends of the crystal on one face and exerting a force in the opposing direction. Blue arrows show the direction of three-point bending. **d** Microphotographs of plastically bent crystals along with the (110) and ($\bar{1}$10) faces, and brittle along the CP chains, i.e., the (001) face. Blue arrows show the direction of three-point bending as in **c**.

As a model system, we studied the HP response of a plastically bendable 1D coordination polymer (CP) [Zn(μ-Cl)_2_(3,5-dichloropyridine)_2_]_*n*_ (**1**), Fig. 1. With nearly isostructural crystal packing as compared with other mechanically flexible CPs^3,16^, (**1**) crystallises in the tetragonal space group $P\bar{4}b2$, with covalent CP chains aligned along the crystallographic *c* axis. Single crystals of (**1**) deform plastically across two crystallographic faces when exposed to three-point bending (Fig. 1c, d)^16^. The reported vibrational spectra indicated that no structural changes occur within the CP chains during bending. Instead, the high mosaicity, which resulted from bending was ascribed to inter-CP chain distortions, i.e., to a discrete response of the crystal lattice to mechanical stress. Moreover, atomic force microscopy revealed a loss of the lattice mechanical integrity at increased loading. By subjecting (**1**) to extremes of pressure, we seek to unravel if the bulk plastic flexibility relates to the atomistic structural distortions under high-pressure conditions, and whether the collapse of lattice integrity under three-point bending foreshadows the high-pressure amorphization reported for other metal-organic frameworks^26,27^.

## Results and discussion

**Fig. 2:**
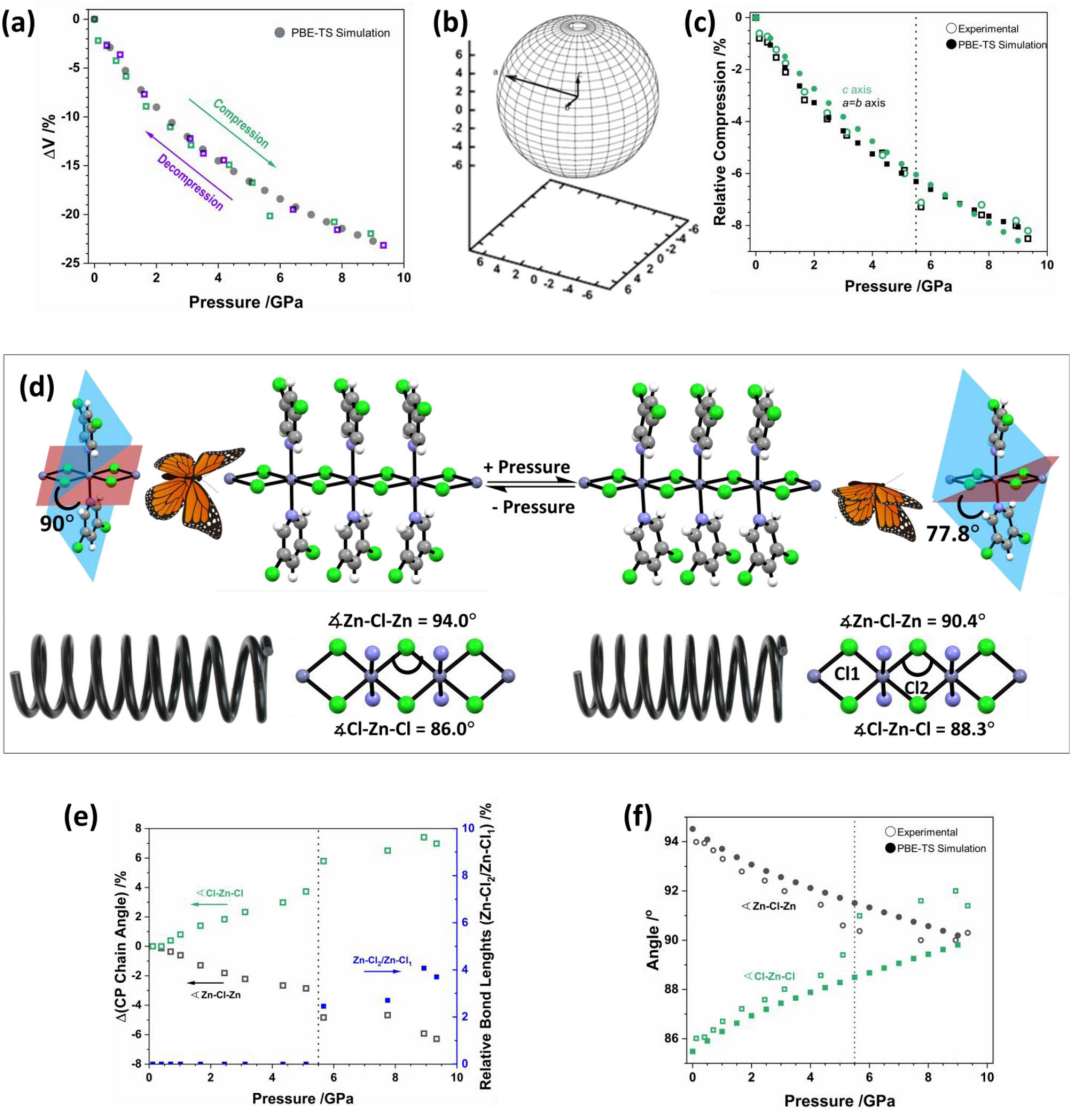
Effects of high pressure on the crystallographic structure of (**1**). **a** The relative change of the crystallographic unit-cell volume during (green) compression and (purple) subsequent decompression. Unit-cell volumes obtained by PBE-TS simulations are shown in black. The phase transition point is indicated by a vertical dotted line. **b** The compressibility indicatrix obtained from the pressure derivatives of the unit-cell axes of (**1**) to 9.35 GPa. The unit-cell axes are indicated by the inset arrows. **c** The relative change of the crystallographic unit-cell axes of (**1**) during compression. Experimental values are shown as open symbols (black squares – *a*, *b* axes; green circles – *c* axis) and PBE-TS values are indicated as closed symbols (black squares – *a*, *b* axes; green circles – *c* axis). The phase transition point is shown with a vertical line. **d** Effects of the crystallographic phase transition on the CP chain geometry. With increasing pressure, the CP chains are compressed as springs. Across the phase transition, a puckering of the chloro-pyridine ligands occurs. This leads to a significant change in angle between the planes of the chloro-pyridine ligands and the CP backbone akin to a butterfly flying motion. $\measuredangle$Zn-Cl-Zn is marked in the CP chains. **e** Evolution of CP chain geometry with increasing pressure; (green) relative change in $\measuredangle$Cl-Zn-Cl, (black) relative change in $\measuredangle$Zn-Cl-Zn, (blue) relative change in Cl-Zn bond lengths. Note that symmetry breaking at the phase transition leads to two unique Zn-Cl distances (Zn-Cl1; Zn-Cl2) as indicated in **d**. No divergence of $\measuredangle$Zn-Cl-Zn is observed over the phase transition. **f** Change in $\measuredangle$Zn-Cl-Zn and $\measuredangle$Cl-Zn-Cl angles as a function of pressure, obtained by experiment and PBE-TS simulations. The phase transition is marked by a vertical line in **e** and **f**.

Our HP X-ray diffraction experiments were conducted using a single crystal of (**1**) loaded into a Merrill-Bassett diamond anvil cell (DAC) with ruby spheres as the pressure calibrant (see Supplementary Note 1). A 1:1 pentane-isopentane mixture was used as a pressure-transmitting medium. With increasing pressure, all crystallographic unit-cell axes compress monotonically (i.e., smoothly), Fig. 2a. The CP chains (*c* axis) compress at a nearly identical rate as the inter-chain voids (*a* and *b*), thereby yielding a spherical compressibility indicatrix, Fig. 2b, c. This so-called indicatrix provides a map of the magnitude of compressibility in each direction^28^. The sphericity of (**1**) compressibility differs markedly from its variable temperature response, which is highly anisotropic (see Supplementary Note 3.6).

Inspection of the atomic structure shows that CP compression occurs as a ‘spring’, with the Cl-Zn-Cl angles expanding with pressure, Fig. 2d, f. Only minimal changes in the Cl-Zn bond lengths are observed, Fig. 2e. The HP response of (**1**) is exceptionally well reproduced by our density functional theory (DFT) calculations at Perdew-Burke-Ernzerhof–Tkachenko and Scheffler (PBE-TS) level, Fig. 2 and Supplementary Note 3.2. No significant changes are observed in the electronic structure of the covalent Zn-Cl-Zn chain upon compression, Supplementary Note 3.4.

Single crystals of (**1**) exhibit unmistakable plasticity during three-point bending Fig. 1c, d^16^. In stark contrast, the HP response of (**1**) is fully reversible, even following compression to 9.34 GPa. During decompression, the crystallographic unit-cell relaxes along the same pressure-volume (p-V) path with no detectable hysteresis, Fig. 2a, b. The complete reversibility and notable compressibility of the CP chains (*c* axis) demonstrate decisively that the bulk mechanical properties exhibited under quasi-hydrostatic compression do not directly dictate the mechanical properties observed during three-point bending.

**Fig. 3:**
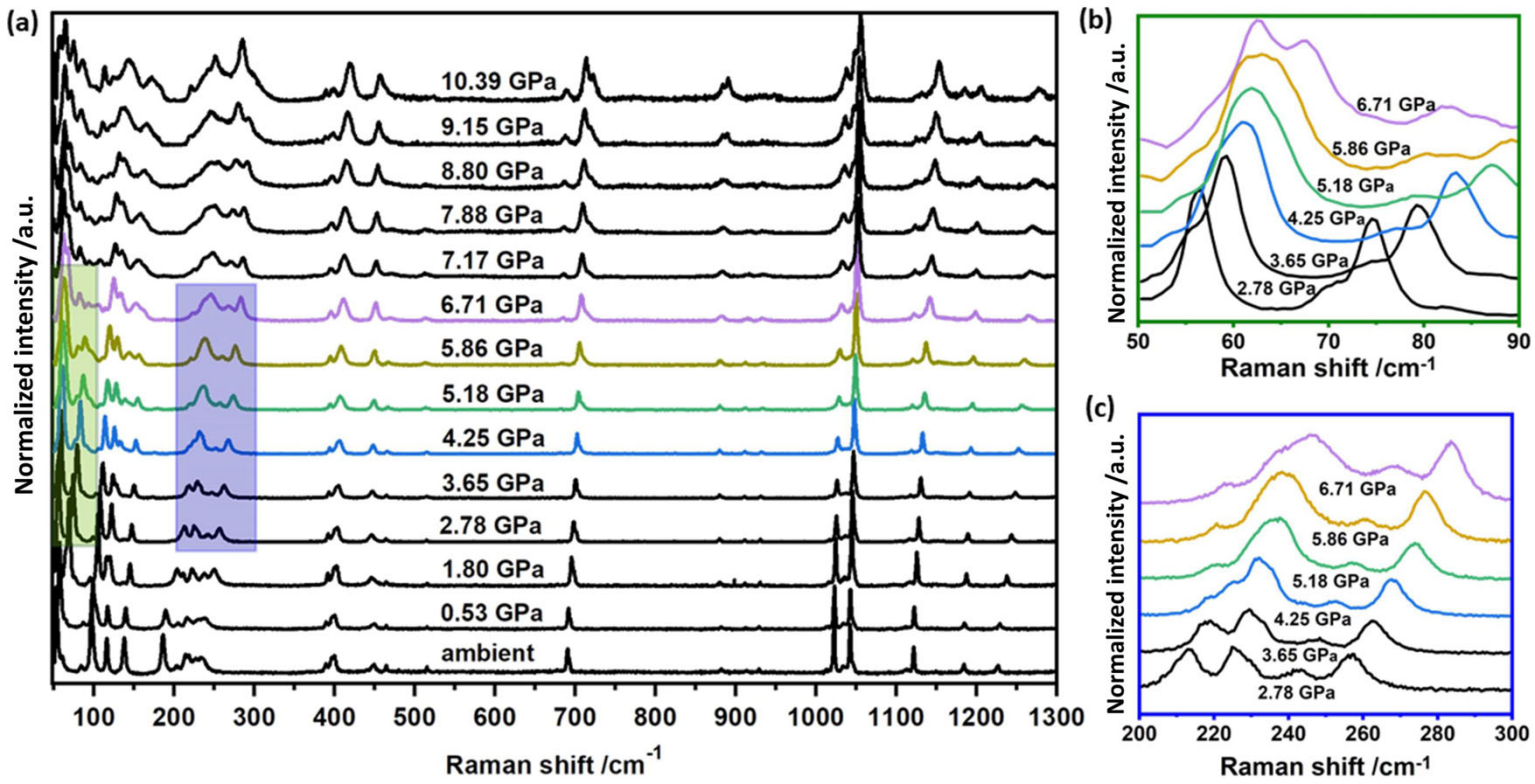
High-pressure Raman spectra collected for a polycrystalline sample of (**1**). **a** Raman spectra collected across complete pressure range; **b**–**c** magnification of selected regions which exhibit band splitting across the phase transition. Full spectra are given in Supplementary Note 5.

Microfocus Raman spectra provided further detail regarding the HP response of a powdered sample of (**1**) (see Supplementary Note 1). The ambient pressure Raman frequencies are reproduced well by our PBE-TS approach, thereby allowing band assignments (see Supplementary Note 4 and 5). With increasing pressure, significant hardening (i.e., blue shift) of the vibrational modes of (**1**) was observed, Fig. 3a. Consistent with the compression of Zn-Cl bonds, the Raman active (symmetry species $E$) Zn-Cl stretching modes harden from *ca*. 93 cm^−1^ at ambient pressure to *ca.* 136 cm^−1^ by 4 GPa. In contrast, the Raman bands associated with intramolecular interactions in the dichloropyridine ligands (i.e., > 300 cm^−1^) exhibit minimal hardening, Fig. 3 and Supplementary Note 5. The mode hardening observed under HP conditions contrasts with our previous three-point-bending results, in which no changes were detected in the Raman spectra during bending^16^. Moreover, compression of (**1**) did not result in the loss of lattice vibrational bands < 100 cm^−1^ (Fig. 3a, b and Supplementary Note 5) previously noted upon three-point-bending. Instead, the Raman band at *ca.* 50 cm^−1^ (wagging of the pyridyl ligands)_,_ which remains unchanged during three-point bending, blueshifts. This shift indicates significant perturbation of the inter-chain interactions upon compression, far beyond the local forces sustained in the crystal during bending.

The pressure-volume (p-V) curves obtained up to 9.34 GPa exhibit no obvious discontinuities. However, deeper analysis of the HP behaviour reveals the onset of a structural phase transition in (**1**) between 4 and 5 GPa, Table 1. Our X-ray diffraction data suggest this to be a continuous transition, reaching only partial conversion by 5.12 GPa. By 5.67 GPa the transition appears complete. The phase transition is associated with a loss of symmetry, with the unit-cell space group symmetry reducing from $P\bar{4}b2$ to $P\bar{4}$. The plane formed from the Zn-Cl-Zn backbone sits orthogonal to the plane formed by either Zn-pyridine in the low-pressure form. In contrast, these same planes jump to internal angles of *ca.* 78^o^ in the high-pressure phase, Fig. 2d–f. Hence structurally, this transition is accompanied by puckering of the dichloropyridine ligands, akin to a ‘butterfly’ motion, Fig. 2d.

**Table 1:** Experimentally determined lattice parameters for (**1**) before and after the phase transition.

	Ambient	4.35 GPa	5.67 GPa
SG	$P\bar{4}b2$	$P\bar{4}b2$	$P\bar{4}$
*a* = *b*/Å	13.8212(6)	13.1008(5)	12.8139(14)
*c* / Å	3.6510(2)	3.4575(2)	3.3912(5)
V /Å^3^	697.43(7)	593.414	556.822

Ambient pressure parameters are taken from Ref. ^16^. The space group (SG) is given in each case.

The structural changes observed across the phase transition break the C_2_ symmetry of the Zn(3,5-dichloropyridine)_2_ moieties parallel to the N-Zn-N vector (see Supplementary Note 3.1). This has the unexpected effect of allowing the Zn-Cl bonds above and below the Zn(3,5-dichloropyridine)_2_ planes to vary independently with pressure, Fig. 2d–f. Whereas before the phase transition all Zn-Cl bonds compress by 0.015 Å/GPa, they compress asymmetrically after the transition, by 0.017 Å/GPa and 0.003 Å/GPa on either side of the pyridyl ligand plane.

Indications of this structural phase transition are also observed in the Raman spectra, Fig. 3b, c. This is notable by the onset of splitting in the Raman bands at *ca*. 60 cm^−1^ (Fig. 3b) and 220 cm^−1^ (Fig. 3c). Splitting of Raman bands is typical of structural phase transitions, which result in a decrease of symmetry. According to our PBE-TS simulations, these bands correspond to the wagging of the pyridine ligands and an out-of-plane pyridine bending mode, respectively.

**Fig. 4:**
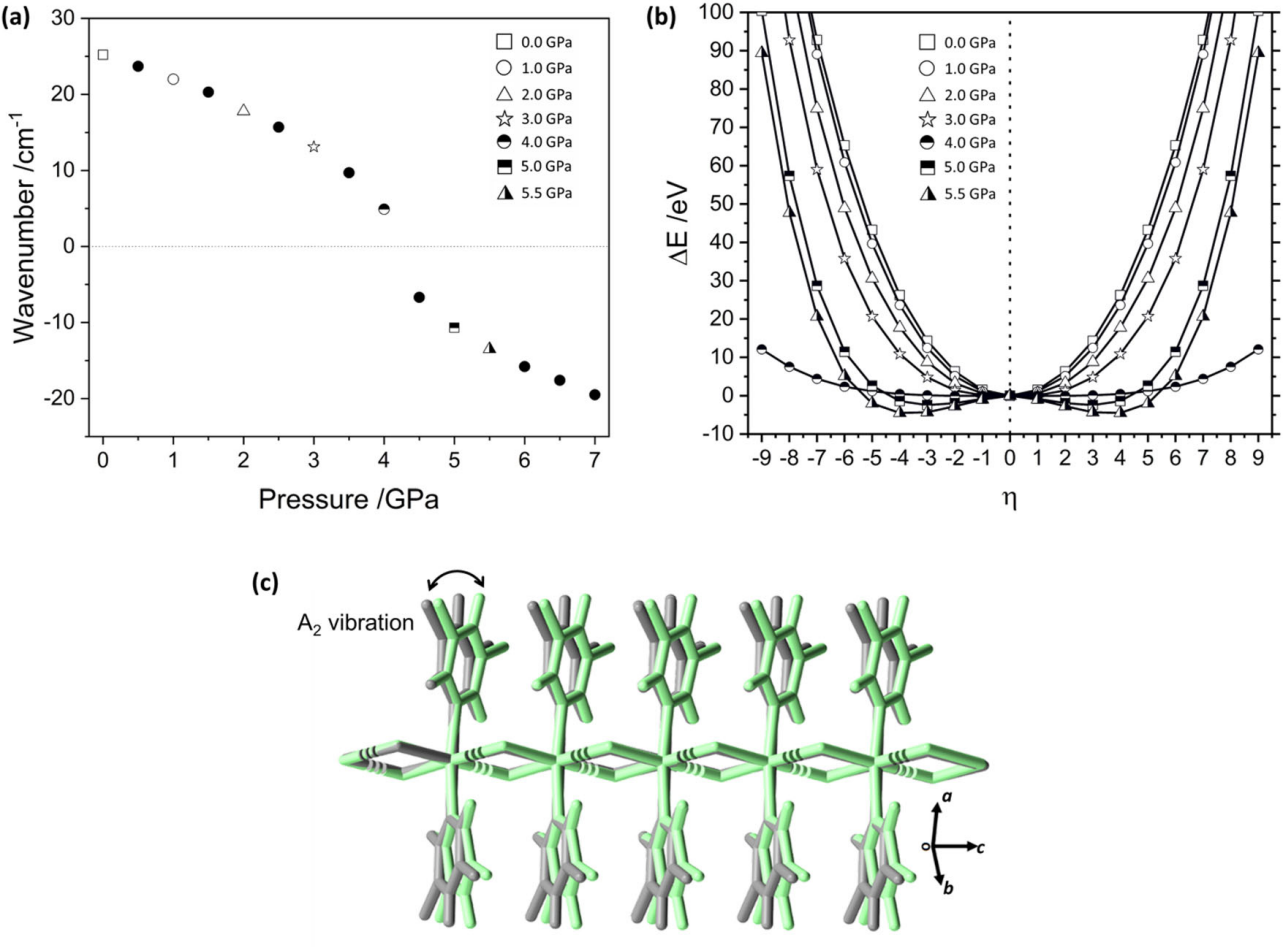
Mode softening of the lowest frequency *A*_2_ vibrational mode in (**1**) with pressure. **a** Vibrational frequency of the* A*_2_ band as a function of pressure. **b** Potential energy surface associated with the lowest frequency *A*_2_ normal mode in the low-pressure phase of (**1**). Displacements are shown as regular displacements along the *A*_2_ mode eigenvector away from equilibrium. Note that the curves for *P* =5 GPa and *P* = 5.5 GPa have been multiplied by 10 for clarity. **c** Overlay of the *A*_2_ normal mode displacement from (grey) *η* = 0 to (green) *η* = 4.

The crystallographic unit-cell contains the same translational symmetry in both the low- and high-pressure phases. Correspondingly, the structural phase transition is dominated by distortion at the Γ-point of reciprocal space. Symmetry reduction from point group *D*_2d_ →*S*_4_ suggests that a symmetry coordinate of *A*_2_ character should be responsible. *A*_2_ modes are not Raman active, and can therefore not be observed in the Raman spectra in Fig. 3. Our simulations do, however, reveal a low-energy (25.2 cm^−1^) vibrational mode with atomic displacements that are consistent with the pyridyl ligand ‘butterfly’ distortion of the structural phase transition. This *A*_2_ vibrational band softens (i.e. redshifts) with pressure and becomes dynamically unstable by 4.5 GPa, Fig. 4a and Supplementary Note 4. This is consistent with the experimentally observed pressure for the phase transition.

By incrementally ‘freezing’ the phonon distortion by factor *η* along the *A*_2_ eigenvector at each pressure, we mapped out the potential energy surface (PES) of the phase transition, Fig. 4b. With increasing pressure, the curvature of the PES decreases, consistent with mode softening. By 4.5 GPa the minimum energy point on the *A*_2_ PES shifts from *η* = 0 to *η* = ±4. Importantly, at the resolution available from our simulations, and within the confines of the harmonic phonon approximation, only a single energetic minimum is found at each pressure. This is consistent with a continuous (second-order) phase transition in (**1**). Consistent with the experiment, our DFT simulations also show complete reversibility of this vibrationally driven phase change upon decompression of high-pressure structure (see Supplementary Note 3). Hence, this structural transition is strongly indicative of the nature of mechanical deformation which occurs in (**1**). Moreover, the deformation of the CP chain which drives the HP phase transition does not occur during three-point bending, as demonstrated by Raman spectroscopy.

Analysis of the PBE-TS eigenvectors obtained from symmetry-constrained DFT calculations shows that the *A*_2_ vibrational mode is polarised almost exclusively along the crystallographic *c* axis (Supplementary Note 4). Correspondingly, compression of the perpendicular axes is predominantly responsible for the observed phase transition (Supplementary Note 6). This is illustrated by simulating the anisotropic compression of (**1**) along the (100) and (010) axes. In this case, softening of the same *A*_2_ mode is observed, albeit at significantly lower pressures (*ca.* 3 GPa). It follows that this phase transition can be observed by anisotropic compression, and its absence during three-point bending suggests that a polymorphic transformation is not responsible for the observed mechanical flexibility. These contrasts reported mechanisms for mechanical bending in molecular crystals^29^, thereby further demonstrating the unique complexity of flexibility in single-crystal coordination polymers.

**Fig. 5:**
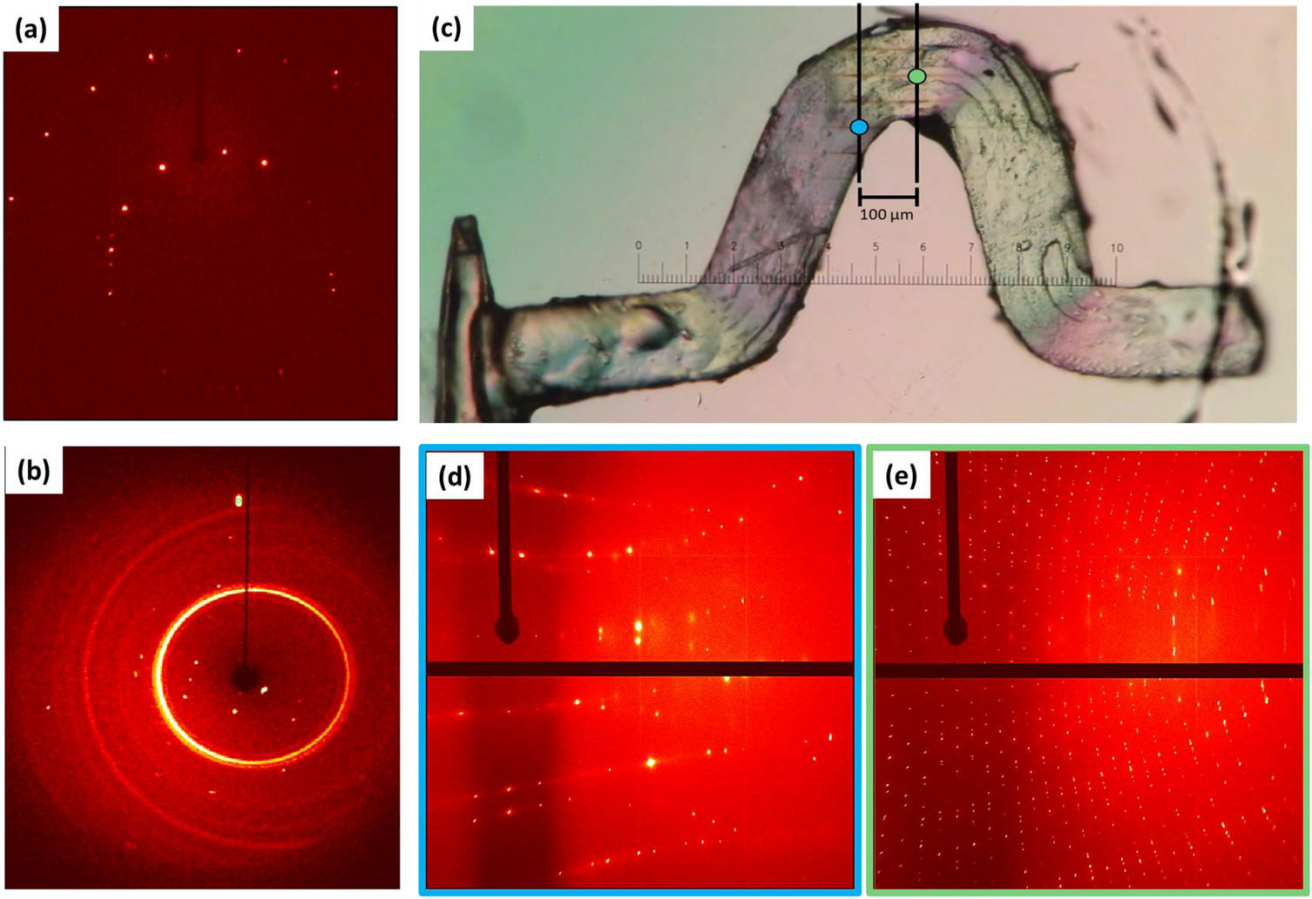
2D X-ray diffraction images collected for single crystals of (**1**) in the *hk0* direction. **a** Data collected by laboratory diffraction for unstressed crystals. **b** Data collected by laboratory diffraction for a single crystal at 9.34 GPa. Note that diffraction rings correspond to scattering from the gasket of the high-pressure cell. **c** Data collected by microfocus synchrotron diffraction within the bent region of the displayed plastically bent crystal. Diffraction images are shown as collected in the centre (**e**) of the bend, and 100 µm displaced from the bend (**d**). Note each increment on the scale bar corresponds to 8.33 µm. Further microfocus diffraction images are shown in Supplementary Note 7.

With an understanding of the high-pressure response of (**1**) we, therefore, sought to elucidate the local conditions present within the bent crystals. Our microfocus X-ray diffraction experiments (0.922 × 3.67 μm beam size) on the bent (i.e., anisotropically stressed) portion of single crystals of (**1**) show that mechanical bending leads to a significant increase in the number of crystalline domains, Fig. 5. No notable changes in the diffraction profiles were observed across the bend, from the convex to the concave surface (see Supplementary Note 7). In all cases, our data suggest a higher degree of mosaicity in the *ac* and *bc* planes, i.e., between the CP chains (see Supplementary Note 7.1). This is consistent with our previously proposed ‘spaghetti model’^16^, which requires the intertwining of these CP chains along with the *a* and *b* axes. Remarkably, the observed mosaicity in all directions was found to decrease with increasing distance from the bent region, Fig. 5d, e, thereby suggesting that the number of crystalline domains increases as we approach the bent region of the crystal.

Despite the significant mosaicity of the plastically bent single crystals, it was possible to extract a series of unit-cell geometries from across the bent region of the crystal. The unit-cell parameters remained largely unaltered by the plastic bending. An exception is the unit-cell extracted from the convex surface of the crystal, whose volume appears to expand by *ca.* 1% (see Supplementary Note 7.3), presumably owing to tensile stresses over the outer surface or peeling of surface layers. Hence, our studies suggest that the atomistic response of (**1**) to three-point bending does not follow directly from its high-pressure response. Bulk lattice integrity is lost upon three-point bending, with significant mosaicity observed along the long-axis of the single crystals. In contrast, the mechanical response of the same lattice to quasi-hydrostatic compression is entirely elastic, with no lasting deformation. Hence, our findings demonstrate two distinctly different bulk responses to mechanical stress in the same crystal lattice, and two markedly different material response properties.

In summary, we report here a high-pressure study of a known mechanically flexible coordination polymer, [Zn(μ-Cl)_2_(3,5-dichloropyridine)_2_]_*n*_, (**1**). Despite significant crystallographic anisotropy, single-crystal X-ray diffraction revealed that all three crystallographic axes undergo identical relative compression. Moreover, the quasi-hydrostatic compression of (**1**) to 9.35 GPa is completely reversible. Upon decompression, the unit-cell parameters of (**1**) relax with no detectable hysteresis. This reversibility is remarkable considering the marked plastic response of (**1**) to three-point bending. Both X-ray diffraction and microfocus Raman spectroscopy suggest the onset of a structural phase transition between 4 and 5 GPa. This phase transition corresponds to distortion of the dichloropyridine ligands and results in loss of crystallographic symmetry. Symmetry mode analysis and DFT-D simulations confirm the critical role of a low-frequency vibrational mode in driving this phase transition by softening at increased pressure. This soft mode is polarised along the crystallographic *c* axis, and hence compression of the *a* and *b* axes drives the phase transition. However, as established by previous Raman spectra, there is no distortion of the CP band during three-point bending. In contrast to reported mechanisms for flexibility in molecular crystals, the observed phase transition does not appear to be responsible for the mechanical flexibility thereby highlighting the unique complexity of flexibility mechanisms in single-crystal CPs. Our microfocus synchrotron X-ray diffraction results suggest that bending results in the formation of microdomains within the bent region, consistent with our previous ‘spaghetti’ model for flexibility in 1D CPs. Our combined experimental and computational studies provide a strong indication for the overall disparate mechanical responses of bulk flexibility and quasi-hydrostatic compression within the same crystal lattice. We suspect this unique structural response under different mechanical stress regimes to be a general feature of plastically bendable materials.

## Methods

High-quality single crystals of [Zn(µ-Cl)_2_(3,5-Cl_2_Py)_2_]_*n*_ (**1**) were prepared by a stirring reaction of zinc chloride with 3,5-dichloropyridine in ethanol and followed by slow evaporation from the same solvent within 7 days. A suitable crystal was loaded into a Merrill-Bassett DAC with 1:1 pentane:isopentane as pressure-transmitting medium. Ruby spheres were used for pressure calibration. Single-crystal X-ray diffraction was collected on a Bruker D8 Venture X-ray diffractometer using Mo-K_α_ radiation at room temperature condition (*ca* 297 K). Full details are given in Supplementary Note 1. Microfocus synchrotron X-ray diffraction experiments were conducted at the BL40XU beamline, SPring-8 (Japan), using 0.81042 Å radiation and beam size of 0.922 × 3.67 μm. High-pressure Raman spectra were collected using a powdered sample loaded in a Diacell One20DAC with 1:1 pentane:isopentane as pressure-transmitting medium (see Supplementary Note 1). Ruby fluorescence was used for pressure calibration. An excitation wavelength of 532 nm was used for spectroscopic measurements. DFT calculations were performed within the CASTEP v19.11 suite using the exchange-correlation functional of PBE with the dispersion correction of TS. Electron-nuclear interactions were modelled using norm-conserving pseudopotentials generated ‘on-the-fly’ within the CASTEP code. Phonon calculations were performed at the Brillouin zone centre by linear response methods. Full computational details are provided in Supplementary Note 2.

## Supplementary information

Supplementary Information

## Data Availability

All relevant data are included in the paper and its supplementary information files. The X-ray crystallographic coordinates for structures reported in this study have been deposited at the Cambridge Crystallographic Data Centre (CCDC), under deposition numbers 2020152-2020172. These data can be obtained free of charge from The Cambridge Crystallographic Data Centre via www.ccdc.cam.ac.uk/data_request/cif.
