# Supplementary material for: High-pressure reversibility in a plastically flexible coordination polymer crystal
Source: Nat Commun. 2021 Jun 23;12:3871. doi: 10.1038/s41467-021-24165-x (PMC8222229; doi:10.1038/s41467-021-24165-x)
Supplement: Supplementary file 1 — Supplementary Information [file 41467_2021_24165_MOESM1_ESM.pdf]

High-Pressure Reversibility in a Plastically Flexible Coordination Polymer Crystal

Xiaojiao Liu,*†[1] Adam A.L. Michalchuk,*†[2] Biswajit Bhattacharya,*[2] Nobuhiro Yasuda,[3] Franziska Emmerling,[2] and Colin R. Pulham[1]

[1] EaStChem School of Chemistry and Centre for Science at Extreme Conditions (CSEC), University of Edinburgh, Edinburgh, UK.

[2] Federal Institute for Materials Research and Testing (BAM), Berlin, Germany.

[3] Japan Synchrotron Radiation Research Institute (JASRI) 1-1-1, Kouto, Sayo-cho, Sayo-gun, Hyogo Japan

† These authors contributed equally

Correspondence: xiaojiao.liu@ed.ac.uk; adam.michalchuk@bam.de; biswajit.bhattacharya@bam.de

Supplementary Note 1| Experimental Details

Supplementary Note 1.1| Materials

Zinc(II) chloride (99.999%; CAS No: 7646-85-7) and 3,5-dichloropyridine (98%; CAS No: 2457-47-8) were purchased from Sigma-Aldrich and were used without further purification. AR grade Ethanol (99%, CHEMSOLUTE) was used as received.

Supplementary Note 1.2| Crystal Growth

Zinc(II)chloride (2 mmol, 0.273 g) was dissolved in 20 mL ethanol and 3,5-dichloropyridine (4 mmol, 0.592 g) was dissolved in 20 mL of ethanol. The resulting colorless solutions were mixed together with constant stirring for 3 hours at room temperature. After seven days, colorless block shaped crystals were obtained from the filtrate, which corresponded to the product, $[Zn(\mu\text{-}Cl)_2(3,5\text{-}ichloropyridine)_2]_n$ (**1**).

Yield 85%. Anal. Calc. for $C_{10}H_6Cl_6N_2Zn$ (MW: 432.26): C, 27.79; H, 1.40; N, 6.48%. Found: C, 27.35; H, 1.29; N, 6.36%.

Supplementary Note 1.3| High Pressure X-ray Diffraction

High pressure single crystal X-ray diffraction was conducted on a Bruker D8 Venture X-ray diffractometer equipped with a CCD detector. A crystal of sufficient quality for X-ray diffraction was loaded into a Merrill-Bassett diamond anvil cell (DAC) with 1:1 pentane:isopentane as pressure transmitting medium, Supplementary Figure 1.1. A stainless steel gasket was used, with a hole diameter of 300 μm. Ruby spheres were used for pressure calibration.[1] Data were collected using Mo-K_α radiation at room temperature conditions (*ca* 297 K). Full details of the collected diffraction data are given in Supplementary Note 3.3. Data reduction and application of Lorentz polarization and absorption corrections were performed with the Bruker APEX III software package. Structure determination was done within the Olex2 software,[2] using the ShelXT program[3] using Intrinsic Phasing methods. The refinement was performed by the ShelXL program[4] using least squares minimization.

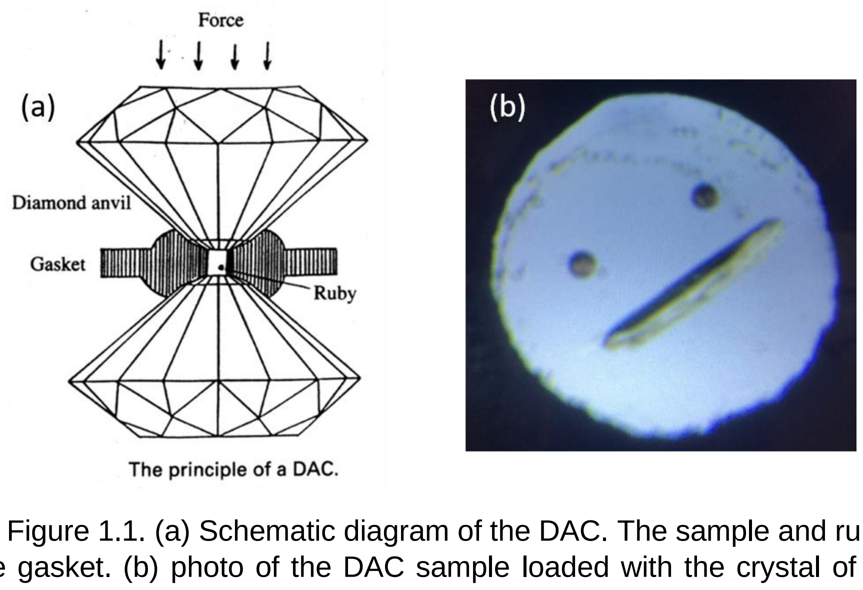

Supplementary Figure 1.1. (a) Schematic diagram of the DAC. The sample and ruby spheres are confined by the gasket. (b) photo of the DAC sample loaded with the crystal of $[Zn(\mu\text{-}Cl)_2(3,5\text{-}ichloropyridine)_2]_n$ and two ruby spheres. The gasket hole diameter is *ca* 300 μm.

Supplementary Note 1.4| High Pressure Raman Spectroscopy

High pressure Raman spectra were collected using a Diacell One20DAC. To ensure averaging of the Raman signal, a powdered sample of $[Zn(\mu\text{-}Cl)_2(3,5\text{-ichloropyridine})_2]_n$ was loaded into the DAC with 1:1 pentane:isopentane mixture as pressure transmitting medium. Ruby chips were used as pressure calibrant. Raman spectroscopic measurements were performed using a Horiba Jobin Yvon Labram HR800 Raman microscopy system. This includes an Olympus BX41 microscope. The white-light microscopic was used in conjunction with a 10×/N.A. = 0.25 objective lens (with N.A. denoting the numerical aperture) to acquire images of the loaded material. Moreover, these images were used to select appropriate locations for spectroscopic measurements. Raman excitation was performed by a continuous-wave diode-pumped solid-state laser with λ=532 nm, which was focused onto the sample by a 50×/N.A. = 0.75 objective. Scattered light was collected by the same lens. Reflected light and Rayleigh scatter were filtered by a bandpass filter, and the Stokes-shifted Raman-scattered light was dispersed by an 1800 mm^{-1} grating. The signal was detected by a liquid-nitrogen-cooled charge-coupled device (CCD) camera operated at -130°C. The spectrometer entrance slit was 100 µm wide with a confocal pinhole 1000 µm in diameter. In this configuration, the spectral resolution varied between 0.5 cm^{-1} per CCD pixel (at 100 cm^{-1} Raman shift) and 0.3 cm^{-1} per CCD pixel (at 3800 cm^{-1} Raman shift) within the observed spectral range. In this configuration, the laser spot resolution is approx. 1 µm laterally 20 µm vertically.[5]

Supplementary Note 1.5| Microfocus Synchrotron X-ray Diffraction

Microfocus synchrotron X-ray diffraction experiments were conducted on the the precision diffractometer equipped with a hybrid photon counting detector, EIGER X 1M detector (DECTRIS) in the SPring-8 BL40XU beamline.[6,7] The X-ray beam (λ = 0.81042 Å) was focused to 0.922 (vertical) x 3.67 (horizontal) µm using a zone plate. To irradiate only a focused beam, a 30-µm diameter sorting aperture and a 40-µm-diameter centre stop were used.

The bent crystal was attached on the glass needle with superglue. The focused X-ray was incident normal to the bending plane (ω = 0°). The ω range, oscillation angle (Δω) and exposure time were ±90°, 0.5° and 0.5 s, respectively. Data were collected at room temperature. The measured position was moved from outside to inside of bend by 50 µm, and 360 diffraction images were collected from each position. After the measurement of the centre of the bend, the crystal was shift to 100 µm displaced from the bend and the same diffraction measurements were carried out. Data reduction and unit cell determination were performed with the CrysAlisPro software package.

Supplementary Note 2| Computational Details

Density Functional Theory (DFT) calculations were performed with the CASTEP software (v19.11).[8] The initial atomic coordinates and unit cell parameters were taken from the ambient pressure experimental structure (See Section S1). The electronic structure was expanded in plane waves to a kinetic energy cut-off of 1100 eV for the wave function. The Brillouin zone was sampled on a Monkhorst Pack grid[9] of spacing 0.05 $Å^{-1}$ which gave dimensions 2 x 2 x 6 (3 *k*-points in the irreducible Brillouin zone). The nuclear charge was modelled norm-conserving pseudopotentials, as generated 'on-the-fly' within the CASTEP suite. DFT calculations were performed with the exchange-correlation functional of Perdew-Burke-Ernzerhof (PBE)[10] and dispersion interactions were included with dispersion correction of Tkachenko and Scheffler (TS).[11] The structure was fully relaxed until the residual atomic forces converged < 5 x 10^{-4} eV. $Å^{-1}$, the electronic wave function converged to < 1 x 10^{-10} eV and the bulk stress < 0.01 GPa. Vibrational frequencies were calculated at the Brillouin zone centre *via* linear response theory.[12] LO-TO splitting at $\boldsymbol{k} \rightarrow 0$ was not considered. The acoustic sum rule was imposed analytically. Hydrostatic compression was performed incrementally, wherein pressure point p_n was optimized based on the simulated structure at p_{n-1}. Pressure steps of 0.5 GPa were used, with relaxation criteria for geometry and phonon calculations as described above. For anisotropic compression simulations, tetragonal space group symmetry was conserved such that $a = b$ and $\alpha = \beta = \gamma = 90^o$. An external potential was placed along the *z* vector (crystallographic *c*-axis), and the full cell was left to relax.

For calculation of the electronic band structures, the experimental geometries were used, without further relaxation. The electronic wave function was calculated using QuantumESPRESSO[13] within the projector augmented wave (PAW) framework.[14] The wavefunction was expanded in plane waves to a kinetic energy cut-off of 60 Ry and a charge density cutoff of 480 Ry. The electronic structure was sampled on a 2 x 2 x 6 Γ-centred Monkhorst-Pack grid.[9] A subsequent non-SCF calculation was performed to include electronic states in the conduction band. The wavefunction was subsequently analysed in LOBSTER v4.0 for calculation of the crystal overlap Hamilton populations.[15–17] Projections were made for configurations H (1s), C (2s 2p), N (2s 2p), Cl (3s 3p), Zn (3d 4s 4p) yielding absolute charge spilling < 1% for all structures.

Supplementary Note 3| High Pressure Crystallographic Structure

Supplementary Note 3.1| Experimental High-Pressure Structures

The unit cell parameters obtained from single crystal X-ray diffraction for compression of (**1**) are tabulated in Supplementary Table 3.1. Owing to crystallographic symmetry, the crystallographic *a* and *b* axes remain equivalent across the whole pressure range. Upon decompression, the unit cell axes appear to relax along the same pressure-volume curve, indicating complete elasticity of the lattice up to 9.34 GPa with no detectable hysteresis. This is in significant contrast to the exceptional plasticity exhibited by this same compound under three-point bending experiments.[18]

Supplementary Table 3.1: Experimental high-pressure unit cell geometry for (**1**). The unit cells of all crystal structures are tetragonal with $\alpha = \beta = \gamma = 90^o$. Experimental error is given in brackets.

p/GPa	***a*** **/Å**	***b*** **/Å**	***c*** **/Å**	**V /Å³**
		Compression		
0.12	13.7105(8)	13.7105(8)	3.6288(3)	682.13(10)
0.40	13.6865(5)	13.6865(5)	3.6238(2)	678.81(6)
0.70	13.6088(4)	13.6088(4)	3.6058(2)	667.79(5)
1.02	13.5303(4)	13.5303(4)	3.5867(1)	656.61(4)
1.67	13.3827(4)	13.3827(4)	3.5466(1)	635.18(4)
2.45	13.2818(4)	13.2818(4)	3.5170(2)	620.42(5)
3.12	13.1946(4)	13.1946(4)	3.4898(2)	607.57(5)
4.35	13.1008(5)	13.1008(5)	3.4575(2)	593.41(6)
5.10	13.0084(5)	13.0084(5)	3.4318(2)	580.72(6)
5.67	12.8139(14)	12.8139(14)	3.3912(5)	556.82(15)
7.75	12.7712(19)	12.7712(19)	3.3879(6)	552.58(19)
8.93	12.715(5)	12.715(5)	3.3657(15)	544.1(5)
9.34	12.645(4)	12.645(4)	3.3514(13)	535.9(4)
		Decompression		
7.85	12.733(5)	12.733(5)	3.3737(17)	547.0(5)
6.42	12.868(2)	12.868(2)	3.3911(8)	561.5(2)
4.18	13.1240(17)	13.1240(17)	3.4645(6)	596.72(19)
3.52	13.154(3)	13.154(3)	3.4768(10)	601.6(3)
3.09	13.2291(17)	13.2291(17)	3.4983(6)	612.23(19)
1.61	13.450(3)	13.450(3)	3.5590(11)	643.8(3)
0.83	13.644(2)	13.644(2)	3.6103(7)	672.1(2)
0.40	13.690(2)	13.690(2)	3.6211(7)	678.7(2)

* Unit cell parameters under ambient conditions, as reported in Ref [18], are: a = b = 13.8212(6) Å, c = 3.6510(2) Å, V = 697.43(7) $Å^3$.

The atom labelling scheme used for discussion throughout this document is shown in Supplementary Figures 3.1 and 3.2. With increasing pressure, the covalent CP network of (**1**) varies as shown in, Supplementary Table 3.2. Below *ca.* 5 GPa, the Zn…Cl bonding interactions, as well as ∡Cl-Zn-Cl and ∡Zn-Cl-Zn, above and below the plane of the pyridyl ligands vary symmetrically. This is consistent with the crystallographic symmetry $P\bar{4}b2$. Above *ca.* 5 GPa, the

molecular symmetry is broken, and these interactions vary independently. The crystallographic details for each structure are reported in Supplementary Table 3.3, a more detailed bond list for each structure is given in Supplementary Tables 3.4-3.25.

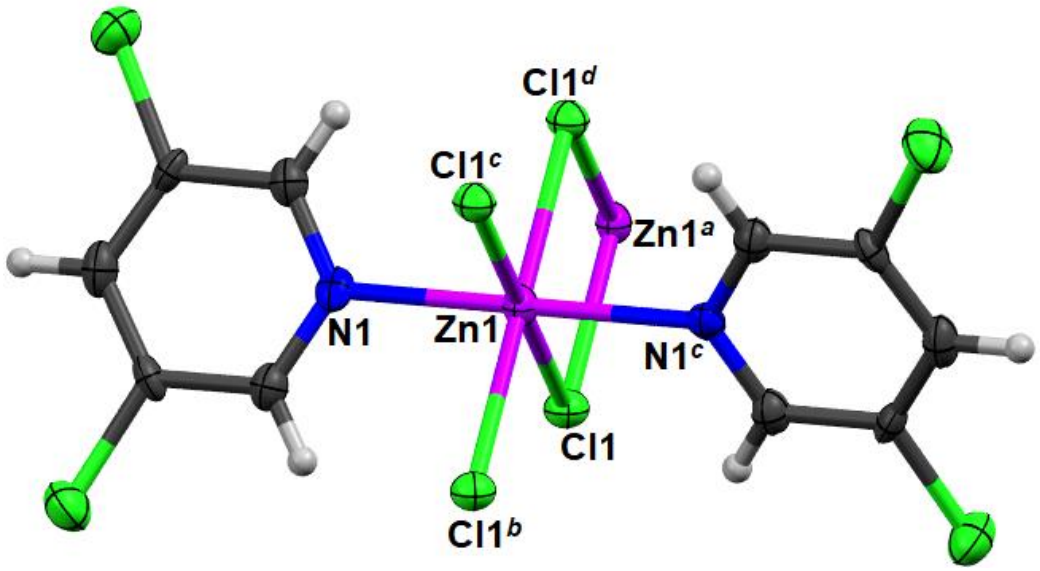

Supplementary Figure 3.1. Thermal ellipsoid drawing (50% probability ellipsoids) of [Zn(μ-Cl)$_2$(3,5-ichloropyridine)$_2$]$_n$ (**1**) at 0.12 GPa showing atom labelling scheme. Atoms are colored as: Zn – pink; Cl – green; N – blue; C – grey; and H – white.

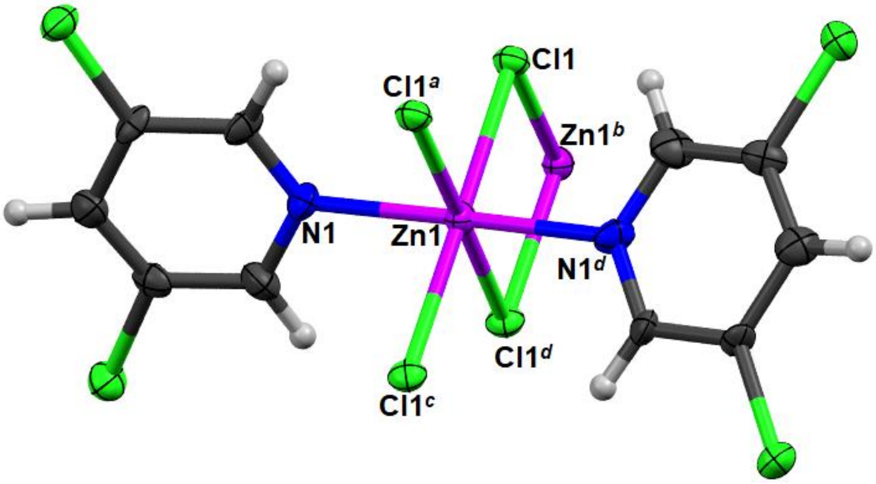

Supplementary Figure 3.2. Thermal ellipsoid drawing (50% probability ellipsoids) of [Zn(μ-Cl)$_2$(3,5-ichloropyridine)$_2$]$_n$ (**1**) at 5.67 GPa showing atom labelling scheme. Atoms are colored as: Zn – pink ; Cl – green; N – blue; C – grey; and H – white.

Supplementary Table 3.2: Experimental molecular geometric parameters for (**1**) as a response to increasing pressure. Selected bond distances [r(A...B)] and [∡A-B-C] are given. Note Cl_1 = $Cl1^c$ and $Cl1^d$ in Supplementary Figure 3.1; Cl_2 = $Cl1^b$ and Cl1 in Supplementary Figure 3.1

p/GPa	r(Zn…Cl_1) /Å	r(Zn…Cl_2) /Å	r(Zn…N) /Å	∡Cl_1-Zn-Cl_1 /°	∡Zn-Cl_1-Zn /°	∡Cl_2-Zn-Cl_2 /°	∡Zn-Cl_2-Zn /°
				Compression			
0.12	2.4810(8)	2.4810(8)	2.191(4)	86.01(3)	93.99(4)	86.01(3)	93.99(4)
0.40	2.4787(11)	2.4787(11)	2.191(5)	86.06(4)	93.94(6)	86.06(4)	93.94(6)
0.70	2.4722(11)	2.4722(11)	2.198(5)	86.35(4)	93.65(6)	86.35(4)	93.65(6)
1.02	2.4663(15)	2.4663(15)	2.193(8)	86.70(5)	93.30(7)	86.70(5)	93.30(7)
1.67	2.4489(10)	2.4489(10)	2.169(5)	87.21(4)	92.79(5)	87.21(4)	92.79(5)
2.45	2.4360(11)	2.4360(11)	2.160(5)	87.58(4)	92.42(5)	87.58(4)	92.42(5)
3.12	2.4259(13)	2.4259(13)	2.146(7)	88.01(4)	91.99(6)	88.01(4)	91.99(6)
4.35	2.4148(11)	2.4148(11)	2.146(5)	88.57(4)	91.44(5)	88.56(8)	91.43(8)
5.10	2.414(10)	2.414(10)	2.06(4)	89.4(7)	90.6(7)	89.4(7)	90.6(7)
5.67	2.361(5)	2.419(5)	2.112(17)	90.99(17)	90.37(17)	88.3(2)	90.37(15)
7.75	2.362(6)	2.429(6)	2.11(2)	91.6(2)	90.0(2)	88.4(3)	90.0(2)
8.93	2.338(12)	2.421(12)	2.12(3)	92.0(4)	90.0(5)	88.0(5)	90.0(4)
9.34	2.329(12)	2.399(12)	2.08(3)	91.4(4)	90.3(4)	88.1(6)	90.3(4)
				Decompression			
7.85	2.347(10)	2.434(10)	2.10(2)	92.4(4)	89.7(4)	88.2(5)	89.7(4)
6.42	2.358(16)	2.416(16)	2.10(4)	90.9(7)	90.5(5)	90.9(7)	90.5(5)
4.18	2.413(4)	2.413(4)	2.147(18)	88.2(3)	91.8(3)	88.3(3)	91.8(3)
3.52	2.408(5)	2.408(5)	2.11(2)	87.59(16)	92.4(2)	87.59(16)	92.4(2)
3.09	2.4266(19)	2.4266(19)	2.159(11)	87.76(6)	92.25(9)	87.76(6)	92.25(9)
1.61	2.446(4)	2.446(4)	2.172(19)	86.63(13)	93.37(18)	86.63(13)	93.37(18)
0.83	2.466(4)	2.466(4)	2.20(2)	85.91(13)	94.09(19)	85.91(13)	94.09(19)
0.40	2.477(3)	2.477(3)	2.192(14)	86.06(10)	93.94(14)	86.06(10)	93.94(14)

Supplementary Table 3.3. Crystallographic and structural refinement parameters of [Zn(μ-Cl)$_2$(3,5-ichloropyridine)$_2$]$_n$ (**1**) at different pressure.

Pressure	Ambient	0.12 GPa	0.40 GPa	0.70 GPa	1.02 GPa
Temperature	RT	RT	RT	RT	RT
Formula	$C_{10}H_6Cl_6N_2Zn$	$C_{10}H_6Cl_6N_2Zn$	$C_{10}H_6Cl_6N_2Zn$	$C_{10}H_6Cl_6N_2Zn$	$C_{10}H_6Cl_6N_2Zn$
Formula Weight	432.24	432.24	432.24	432.24	432.24
Crystal System	Tetragonal	Tetragonal	Tetragonal	Tetragonal	Tetragonal
Space group	P-4b2	P-4b2	P-4b2	P-4b2	P-4b2
a/ Å	13.8212(6)	13.7105(8)	13.6865(5)	13.6088(4)	13.5303(4)
b/Å	13.8212(6)	13.7105(8)	13.6865(5)	13.6088(4)	13.5303(4)
c/ Å	3.6510(2)	3.6288(3)	3.6238(2)	3.6058(2)	3.5867(1)
α/°	90	90	90	90	90
β/°	90	90	90	90	90
γ/°	90	90	90	90	90
V/ $Å^3$	697.43(7)	682.13(10)	678.81(6)	667.79(5)	656.61(4)
Z	2	2	2	2	2
D_c/ g cm^{-3}	2.058	2.105	2.115	2.150	2.186
μ /mm^{-1}	2.892	2.957	2.972	3.021	3.072
F(000)	424	424	424	424	424
θ range/°	2.9-25.6	2.1-26.4	2.1-26.4	2.1-26.4	3.4-24.7
Reflections collected	7836	3480	3444	3941	1747
Unique reflections	664	390	397	385	241
Reflections $I > 2\sigma(I)$	663	347	327	315	193
R_{int}	0.034	0.036	0.043	0.052	0.053
goodness-of-fit (F^2)	1.26	1.13	1.10	1.12	1.06
*R*1 ($I > 2\sigma(I)$)	0.0167	0.0210	0.0282	0.0285	0.0263
*wR*2($I > 2\sigma(I)$)	0.0430	0.0449	0.0578	0.0521	0.0506
CCDC No.	1947282	2020154	2020169	2020168	2020152

Pressure	1.67 GPa	2.45 GPa	3.12 GPa	4.35 GPa	5.10 GPa
Temperature	RT	RT	RT	RT	RT
Formula	$C_{10}H_6Cl_6N_2Zn$	$C_{10}H_6Cl_6N_2Zn$	$C_{10}H_6Cl_6N_2Zn$	$C_{10}H_6Cl_6N_2Zn$	$C_{10}H_6Cl_6N_2Zn$
Formula Weight	432.24	432.24	432.24	432.24	432.24
Crystal System	Tetragonal	Tetragonal	Tetragonal	Tetragonal	Tetragonal
Space group	P-4b2	P-4b2	P-4b2	P-4b2	P-4b2
a/ Å	13.3827(4)	13.2818(4)	13.1946(4)	13.1008(5)	13.0084(5)
b/Å	13.3827(4)	13.2818(4)	13.1946(4)	13.1008(5)	13.0084(5)
c/ Å	3.5466(1)	3.5170(2)	3.4898(2)	3.4575(2)	3.4318(2)
α/°	90	90	90	90	90
β/°	90	90	90	90	90
γ/°	90	90	90	90	90
V/ $Å^3$	635.18(4)	620.42(5)	607.57(5)	593.41(6)	580.72(6)
Z	2	2	2	2	2
D_c/ g cm^{-3}	2.260	2.314	2.363	2.419	2.461
μ /mm^{-1}	3.176	3.251	3.320	3.399	3.474
F(000)	424	424	424	424	424

θ range/°	2.2-26.4	2.2-26.3	2.2-26.3	2.2-26.2	2.2-26.4
Reflections collected	3625	3502	3538	3343	2872
Unique reflections	370	357	350	344	315
Reflections $I > 2\sigma(I)$	316	317	309	306	282
R_{int}	0.050	0.040	0.052	0.043	0.059
goodness-of-fit (F^2)	1.14	1.19	1.09	1.10	1.11
$R1$ ($I > 2\sigma(I)$)	0.0264	0.0228	0.0301	0.0275	0.1640
$wR2$($I > 2\sigma(I)$)	0.0406	0.0484	0.0514	0.0540	0.3836
CCDC No.	2020157	2020161	2020153	2020172	2020155

Pressure	5.67 GPa	7.75 GPa	8.93 GPa	9.34 GPa	7.85 GPa^{dcom}
Temperature	RT	RT	RT	RT	RT
Formula	$C_{10}H_6Cl_6N_2Zn$	$C_{10}H_6Cl_6N_2Zn$	$C_{10}H_6Cl_6N_2Zn$	$C_{10}H_6Cl_6N_2Zn$	$C_{10}H_6Cl_6N_2Zn$
Formula Weight	432.24	432.24	432.24	432.24	432.24
Crystal System	Tetragonal	Tetragonal	Tetragonal	Tetragonal	Tetragonal
Space group	P-4	P-4	P-4	P-4	P-4
a/ Å	12.8139(14)	12.7712(19)	12.715(5)	12.645(4)	12.733(5)
b/Å	12.8139(14)	12.7712(19)	12.715(5)	12.645(4)	12.733(5)
c/ Å	3.3912(5)	3.3879(6)	3.3657(15)	3.3514(13)	3.3737(17)
α/°	90	90	90	90	90
β/°	90	90	90	90	90
γ/°	90	90	90	90	90
V/ Å^3	556.82(15)	552.58(19)	544.1(5)	535.9(4)	547.0(5)
Z	2	2	2	2	2
D_c/ g cm^{-3}	2.578	2.598	2.638	2.679	2.624
μ /mm^{-1}	3.623	3.651	3.707	3.764	3.688
F(000)	424	424	424	424	424
θ range/°	2.2-25.3	2.3-22.2	2.3-20.0	2.3-22.6	2.3-20.3
Reflections collected	2590	2381	1275	1335	1097
Unique reflections	561	448	331	414	333
Reflections $I > 2\sigma(I)$	466	377	240	322	229
R_{int}	0.047	0.071	0.087	0.068	0.087
goodness-of-fit (F^2)	1.10	1.08	1.14	1.121	1.09
$R1$ ($I > 2\sigma(I)$)	0.0497	0.0576	0.0799	0.0874	0.0800
$wR2$($I > 2\sigma(I)$)	0.1124	0.1369	0.2294	0.2426	0.2030
CCDC No.	2020156	2020171	2020158	2020159	2020160

Pressure	6.42 GPa^{dcom}	4.18 GPa^{dcom}	3.52 GPa^{dcom}	3.09 GPa^{dcom}	1.61 GPa^{dcom}
Temperature	RT	RT	RT	RT	RT
Formula	$C_{10}H_6Cl_6N_2Zn$	$C_{10}H_6Cl_6N_2Zn$	$C_{10}H_6Cl_6N_2Zn$	$C_{10}H_6Cl_6N_2Zn$	$C_{10}H_6Cl_6N_2Zn$
Formula Weight	432.24	432.24	432.24	432.24	432.24
Crystal System	Tetragonal	Tetragonal	Tetragonal	Tetragonal	Tetragonal

Space group	P-4	P-4b2	P-4b2	P-4b2	P-4b2
a/ Å	12.868(2)	13.1240(17)	13.154(3)	13.2291(17)	13.450(3)
b/Å	12.868(2)	13.1240(17)	13.154(3)	13.2291(17)	13.450(3)
c/ Å	3.3911(8)	3.4645(6)	3.4768(10)	3.4983(6)	3.5590(11)
α/°	90	90	90	90	90
β/°	90	90	90	90	90
γ/°	90	90	90	90	90
V/ Å^3	561.5(2)	596.72(19)	601.6(3)	612.23(19)	643.8(3)
Z	2	2	2	2	2
D_c/ g cm^{-3}	2.557	2.406	2.386	2.345	2.230
μ /mm^{-1}	3.592	3.380	3.353	3.295	3.133
F(000)	424	424	424	424	424
θ range/°	2.2-21.2	2.2-20.8	2.2-22.2	2.2-22.8	2.1-21.0
Reflections collected	1750	2077	1890	2203	1325
Unique reflections	398	214	248	286	227
Reflections $I > 2σ(I)$	329	177	202	233	186
R_{int}	0.060	0.068	0.078	0.060	0.061
goodness-of-fit (F^2)	1.21	1.26	1.40	1.12	1.17
*R*1 ($I > 2σ(I)$)	0.0938	0.0431	0.0578	0.0451	0.0494
*wR*2($I > 2σ(I)$)	0.2393	0.0900	0.1529	0.1018	0.1198
CCDC No.	2020165	2020162	2020163	2020164	2020170

Pressure	0.83 GPa^{dcom}	0.40 GPa^{dcom}
Temperature	RT	RT
Formula	$C_{10}H_6Cl_6N_2Zn$	$C_{10}H_6Cl_6N_2Zn$
Formula Weight	432.24	432.24
Crystal System	Tetragonal	Tetragonal
Space group	P-4b2	P-4b2
a/ Å	13.644(2)	13.690(2)
b/Å	13.644(2)	13.690(2)
c/ Å	3.6103(7)	3.6211(7)
α/°	90	90
β/°	90	90
γ/°	90	90
V/ Å^3	672.1(2)	678.7(2)
Z	2	2
D_c/ g cm^{-3}	2.136	2.115
μ /mm^{-1}	3.001	2.973
F(000)	424	424
θ range/°	2.1-23.8	2.1-26.4
Reflections collected	2504	2919
Unique reflections	305	405
Reflections $I > 2σ(I)$	230	276
R_{int}	0.081	0.092
goodness-of-fit (F^2)	1.22	1.09
*R*1 ($I > 2σ(I)$)	0.0584	0.0586
*wR*2($I > 2σ(I)$)	0.1504	0.1463
CCDC No.	2020166	2020167

RT = Room Temperature; *dcom* = decompression.

Supplementary Table 3.4. Selected bond lengths (Å) and bond angles (°) for (**1**) at ambient pressure

**Bond Lengths**			
Zn1-Cl1	2.4885(4)	Zn1-Cl1[b]	2.4885(4)
Zn1-Cl1[c]	2.4885(4)	Zn1-Cl1[d]	2.4885(4)
Zn1-N1	2.1886(19)	Zn1-N1[c]	2.1886(19)
**Bond Angles**			
Cl1-Zn1-N1	90.00(4)	Cl1-Zn1-Cl1[b]	94.38(1)
Cl1-Zn1-Cl1[c]	85.62(1)	Cl1-Zn1-N1[c]	90.00(4)
Cl1-Zn1-Cl1[d]	180.00	Cl1[b]-Zn1-N1	90.00(4)
Cl1[c]-Zn1-N1	90.00(4)	N1-Zn1-N1[c]	180.00
Cl1[d]-Zn1-N1	90.00(4)	Cl1[b]-Zn1-Cl1[c]	180.00
Cl1[b]-Zn1-N1[c]	90.00(4)	Cl1[b]-Zn1-Cl1[d]	85.62(1)
Cl1[c]-Zn1-N1[c]	90.00(4)	Cl1[c]-Zn1-Cl1[d]	94.38(1)
Cl1[d]-Zn1-N1[c]	90.00(4)	Zn1-Cl1-Zn1[a]	94.38(2)

Symmetry code: *a* = x, y, -1+z; *b* = x, y, 1+z; *c* = 1-x, 2-y, z; *d* = 1-x, 2-y, 1+z.

Supplementary Table 3.5. Selected bond lengths (Å) and bond angles (°) for (**1**) at 0.12 GPa

**Bond Lengths**			
Zn1-Cl1	2.4810(8)	Zn1-Cl1[b]	2.4810(8)
Zn1-Cl1[c]	2.4810(8)	Zn1-Cl1[d]	2.4810(8)
Zn1-N1	2.191(4)	Zn1-N1[c]	2.191(4)
**Bond Angles**			
Cl1-Zn1-N1	90.00(8)	Cl1-Zn1-Cl1[b]	93.99(3)
Cl1-Zn1-Cl1[c]	86.01(3)	Cl1-Zn1-N1[c]	90.00(8)
Cl1-Zn1-Cl1[d]	180.00	Cl1[b]-Zn1-N1	90.00(8)
Cl1[c]-Zn1-N1	90.00(8)	N1-Zn1-N1[c]	180.00
Cl1[d]-Zn1-N1	90.00(8)	Cl1[b]-Zn1-Cl1[c]	180.00
Cl1[b]-Zn1-N1[c]	90.00(8)	Cl1[b]-Zn1-Cl1[d]	86.01(3)
Cl1[c]-Zn1-N1[c]	90.00(8)	Cl1[c]-Zn1-Cl1[d]	93.99(3)
Cl1[d]-Zn1-N1[c]	90.00(8)	Zn1-Cl1-Zn1[a]	93.99(4)

Symmetry code: *a* = x, y, -1+z; *b* = x, y, 1+z; *c* = 2-x, 1-y, z; *d* = 2-x, 1-y, 1+z.

Supplementary Table 3.6. Selected bond lengths (Å) and bond angles (°) for (**1**) at 0.40 GPa

**Bond Lengths**			
Zn1-Cl1	2.4787(11)	Zn1-Cl1^{b}	2.4787(11)
Zn1-Cl1^{c}	2.4787(11)	Zn1-Cl1^{d}	2.4787(11)
Zn1-N1	2.191(5)	Zn1-N1^{c}	2.191(5)
**Bond Angles**			
Cl1-Zn1-N1	90.00(10)	Cl1-Zn1-Cl1^{b}	93.94(4)
Cl1-Zn1-Cl1^{c}	86.06(4)	Cl1-Zn1-N1^{c}	90.00(10)
Cl1-Zn1-Cl1^{d}	180.00	Cl1^{b}-Zn1-N1	90.00(10)
Cl1^{c}-Zn1-N1	90.00(10)	N1-Zn1-N1^{c}	180.00
Cl1^{d}-Zn1-N1	90.00(10)	Cl1^{b}-Zn1-Cl1^{c}	180.00
Cl1^{b}-Zn1-N1^{c}	90.00(10)	Cl1^{b}-Zn1-Cl1^{d}	86.06(4)
Cl1^{c}-Zn1-N1^{c}	90.00(10)	Cl1^{c}-Zn1-Cl1^{d}	93.94(4)
Cl1^{d}-Zn1-N1^{c}	90.00(10)	Zn1-Cl1-Zn1^{a}	93.94(6)

Symmetry code: *a* = x, y, -1+z; *b* = x, y, 1+z; *c* = 2-x, 1-y, z; *d* = 2-x, 1-y, 1+z.

Supplementary Table 3.7. Selected bond lengths (Å) and bond angles (°) for (**1**) at 0.70 GPa

**Bond Lengths**			
Zn1-Cl1	2.4722(11)	Zn1-Cl1^{b}	2.4722(11)
Zn1-Cl1^{c}	2.4722(11)	Zn1-Cl1^{d}	2.4722(11)
Zn1-N1	2.198(5)	Zn1-N1^{c}	2.198(5)
**Bond Angles**			
Cl1-Zn1-N1	90.00(10)	Cl1-Zn1-Cl1^{b}	93.65(4)
Cl1-Zn1-Cl1^{c}	86.35(4)	Cl1-Zn1-N1^{c}	90.00(10)
Cl1-Zn1-Cl1^{d}	180.00	Cl1^{b}-Zn1-N1	90.00(10)
Cl1^{c}-Zn1-N1	90.00(10)	N1-Zn1-N1^{c}	180.00
Cl1^{d}-Zn1-N1	90.00(10)	Cl1^{b}-Zn1-Cl1^{c}	180.00
Cl1^{b}-Zn1-N1^{c}	90.00(10)	Cl1^{b}-Zn1-Cl1^{d}	86.35(4)
Cl1^{c}-Zn1-N1^{c}	90.00(10)	Cl1^{c}-Zn1-Cl1^{d}	93.65(4)
Cl1^{d}-Zn1-N1^{c}	90.00(10)	Zn1-Cl1-Zn1^{a}	93.65(6)

Symmetry code: *a* = x, y, -1+z; *b* = x, y, 1+z; *c* = 1-x, 2-y, z; *d* = 1-x, 2-y, 1+z.

Supplementary Table 3.8. Selected bond lengths (Å) and bond angles (°) for (**1**) at 1.02 GPa

**Bond Lengths**			
Zn1-Cl1	2.4663(15)	Zn1-Cl1^b	2.4663(15)
Zn1-Cl1^c	2.4663(15)	Zn1-Cl1^d	2.4663(15)
Zn1-N1	2.193(8)	Zn1-N1^c	2.193(8)
**Bond Angles**			
Cl1-Zn1-N1	90.00(15)	Cl1-Zn1-Cl1^b	93.30(5)
Cl1-Zn1-Cl1^c	86.70(5)	Cl1-Zn1-N1^c	90.00(15)
Cl1-Zn1-Cl1^d	180.00	Cl1^b-Zn1-N1	90.00(15)
Cl1^c-Zn1-N1	90.00(15)	N1-Zn1-N1^c	180.00
Cl1^d-Zn1-N1	90.00(15)	Cl1^b-Zn1-Cl1^c	180.00
Cl1^b-Zn1-N1^c	90.00(15)	Cl1^b-Zn1-Cl1^d	86.70(5)
Cl1^c-Zn1-N1^c	90.00(15)	Cl1^c-Zn1-Cl1^d	93.30(5)
Cl1^d-Zn1-N1^c	90.00(15)	Zn1-Cl1-Zn1^a	93.30(7)

Symmetry code: *a* = x, y, -1+z; *b* = x, y, 1+z; *c* = 2-x, 1-y, z; *d* = 2-x, 1-y, 1+z.

Supplementary Table 3.9. Selected bond lengths (Å) and bond angles (°) for (**1**) at 1.67 GPa

**Bond Lengths**			
Zn1-Cl1	2.4489(10)	Zn1-Cl1^b	2.4489(10)
Zn1-Cl1^c	2.4489(10)	Zn1-Cl1^d	2.4489(10)
Zn1-N1	2.169(5)	Zn1-N1^c	2.169(5)
**Bond Angles**			
Cl1-Zn1-N1	90.00(10)	Cl1-Zn1-Cl1^b	92.79(4)
Cl1-Zn1-Cl1^c	87.21(4)	Cl1-Zn1-N1^c	90.00(10)
Cl1-Zn1-Cl1^d	180.00	Cl1^b-Zn1-N1	90.00(10)
Cl1^c-Zn1-N1	90.00(10)	N1-Zn1-N1^c	180.00
Cl1^d-Zn1-N1	90.00(10)	Cl1^b-Zn1-Cl1^c	180.00
Cl1^b-Zn1-N1^c	90.00(10)	Cl1^b-Zn1-Cl1^d	87.21(4)
Cl1^c-Zn1-N1^c	90.00(10)	Cl1^c-Zn1-Cl1^d	92.79(4)
Cl1^d-Zn1-N1^c	90.00(10)	Zn1-Cl1-Zn1^a	92.79(5)

Symmetry code: *a* = x, y, -1+z; *b* = x, y, 1+z; *c* = 2-x, 1-y, z; *d* = 2-x, 1-y, 1+z.

Supplementary Table 3.10. Selected bond lengths (Å) and bond angles (°) for (**1**) at 2.45 GPa

**Bond Lengths**			
Zn1-Cl1	2.4360(11)	Zn1-Cl1^{b}	2.4360(11)
Zn1-Cl1^{c}	2.4360(11)	Zn1-Cl1^{d}	2.4360(11)
Zn1-N1	2.160(5)	Zn1-N1^{c}	2.160(5)
**Bond Angles**			
Cl1-Zn1-N1	90.00(10)	Cl1-Zn1-Cl1^{b}	92.42(4)
Cl1-Zn1-Cl1^{c}	87.58(4)	Cl1-Zn1-N1^{c}	90.00(10)
Cl1-Zn1-Cl1^{d}	180.00	Cl1^{b}-Zn1-N1	90.00(10)
Cl1^{c}-Zn1-N1	90.00(10)	N1-Zn1-N1^{c}	180.00
Cl1^{d}-Zn1-N1	90.00(10)	Cl1^{b}-Zn1-Cl1^{c}	180.00
Cl1^{b}-Zn1-N1^{c}	90.00(10)	Cl1^{b}-Zn1-Cl1^{d}	87.58(4)
Cl1^{c}-Zn1-N1^{c}	90.00(10)	Cl1^{c}-Zn1-Cl1^{d}	92.42(4)
Cl1^{d}-Zn1-N1^{c}	90.00(10)	Zn1-Cl1-Zn1^{a}	92.42(5)

Symmetry code: *a* = x, y, -1+z; *b* = x, y, 1+z; *c* = 2-x, 1-y, z; *d* = 2-x, 1-y, 1+z.

Supplementary Table 3.11. Selected bond lengths (Å) and bond angles (°) for (**1**) at 3.12 GPa

**Bond Lengths**			
Zn1-Cl1	2.4259(13)	Zn1-Cl1^{b}	2.4259(13)
Zn1-Cl1^{c}	2.4259(13)	Zn1-Cl1^{d}	2.4259(13)
Zn1-N1	2.146(7)	Zn1-N1^{c}	2.146(7)
**Bond Angles**			
Cl1-Zn1-N1	90.00(13)	Cl1-Zn1-Cl1^{b}	91.99(4)
Cl1-Zn1-Cl1^{c}	88.01(4)	Cl1-Zn1-N1^{c}	90.00(13)
Cl1-Zn1-Cl1^{d}	180.00	Cl1^{b}-Zn1-N1	90.00(13)
Cl1^{c}-Zn1-N1	90.00(13)	N1-Zn1-N1^{c}	180.00
Cl1^{d}-Zn1-N1	90.00(13)	Cl1^{b}-Zn1-Cl1^{c}	180.00
Cl1^{b}-Zn1-N1^{c}	90.00(13)	Cl1^{b}-Zn1-Cl1^{d}	88.01(4)
Cl1^{c}-Zn1-N1^{c}	90.00(13)	Cl1^{c}-Zn1-Cl1^{d}	91.99(4)
Cl1^{d}-Zn1-N1^{c}	90.00(13)	Zn1-Cl1-Zn1^{a}	91.99(6)

Symmetry code: *a* = x, y, -1+z; *b* = x, y, 1+z; *c* = 1-x, -y, z; *d* = 1-x, -y, 1+z.

Supplementary Table 3.12. Selected bond lengths (Å) and bond angles (°) for (**1**) at 4.35 GPa

**Bond Lengths**			
Zn1-Cl1	2.4148(11)	Zn1-Cl1^b	2.4148(11)
Zn1-Cl1^c	2.4148(11)	Zn1-Cl1^d	2.4148(11)
Zn1-N1	2.146(5)	Zn1-N1^c	2.146(5)
**Bond Angles**			
Cl1-Zn1-N1	90.00(10)	Cl1-Zn1-Cl1^b	91.44(4)
Cl1-Zn1-Cl1^c	88.57(4)	Cl1-Zn1-N1^c	90.00(10)
Cl1-Zn1-Cl1^d	180.00	Cl1^b-Zn1-N1	90.00(10)
Cl1^c-Zn1-N1	90.00(10)	N1-Zn1-N1^c	180.00
Cl1^d-Zn1-N1	90.00(10)	Cl1^b-Zn1-Cl1^c	180.00
Cl1^b-Zn1-N1^c	90.00(10)	Cl1^b-Zn1-Cl1^d	88.57(4)
Cl1^c-Zn1-N1^c	90.00(10)	Cl1^c-Zn1-Cl1^d	91.44(4)
Cl1^d-Zn1-N1^c	90.00(10)	Zn1-Cl1-Zn1^a	91.44(5)

Symmetry code: *a* = x, y, -1+z; *b* = x, y, 1+z; *c* = 1-x, 2-y, z; *d* = 1-x, 2-y, 1+z.

Supplementary Table 3.13. Selected bond lengths (Å) and bond angles (°) for 1 at 5.10 GPa

**Bond Lengths**			
Zn1-Cl1	2.414(10)	Zn1-Cl1^b	2.414(10)
Zn1-Cl1^c	2.414(10)	Zn1-Cl1^d	2.414(10)
Zn1-N1	2.06(4)	Zn1-N1^c	2.06(4)
**Bond Angles**			
Cl1-Zn1-N1	90.0(8)	Cl1-Zn1-Cl1^b	90.6(3)
Cl1-Zn1-Cl1^c	89.4(3)	Cl1-Zn1-N1^c	90.0(8)
Cl1-Zn1-Cl1^d	180.00	Cl1^b-Zn1-N1	90.0(8)
Cl1^c-Zn1-N1	90.0(8)	N1-Zn1-N1^c	180.00
Cl1^d-Zn1-N1	90.0(8)	Cl1^b-Zn1-Cl1^c	180.00
Cl1^b-Zn1-N1^c	90.0(8)	Cl1^b-Zn1-Cl1^d	89.4(3)
Cl1^c-Zn1-N1^c	90.0(8)	Cl1^c-Zn1-Cl1^d	90.6(3)
Cl1^d-Zn1-N1^c	90.0(8)	Zn1-Cl1-Zn1^a	90.6(5)

Symmetry code: *a* = x, y, -1+z; *b* = x, y, 1+z; *c* = -x, 1-y, z; *d* = -x, 1-y, 1+z.

Supplementary Table 3.14. Selected bond lengths (Å) and bond angles (°) for (**1**) at 5.67 GPa

**Bond Lengths**			
Zn1-Cl1	2.361(5)	Zn1-Cl1^{a}	2.419(5)
Zn1-Cl1^{c}	2.419(5)	Zn1-Cl1^{d}	2.361(5)
Zn1-N1	2.112(17)	Zn1-N1^{d}	2.112(17)
**Bond Angles**			
Cl1-Zn1-N1	91.3(4)	Cl1-Zn1-Cl1^{a}	90.37(14)
Cl1-Zn1-Cl1^{c}	178.64(17)	Cl1-Zn1-Cl1^{d}	90.99(17)
Cl1-Zn1-N1^{d}	91.3(4)	Cl1^{a}-Zn1-N1	88.7(4)
Cl1^{c}-Zn1-N1	88.7(4)	Cl1^{d}-Zn1-N1	91.3(4)
N1-Zn1-N1^{d}	176.3(5)	Cl1^{a}-Zn1-Cl1^{c}	88.27(17)
Cl1^{a}-Zn1-Cl1^{d}	178.64(17)	Cl1^{a}-Zn1-N1^{d}	88.7(4)
Cl1^{c}-Zn1-Cl1^{d}	90.37(14)	Cl1^{c}-Zn1-N1^{d}	88.7(4)
Cl1^{d}-Zn1-N1^{d}	91.3(4)	Zn1-Cl1-Zn1^{b}	90.37(17)

Symmetry code: *a* = x, y, -1+z; *b* = x, y, 1+z; *c* = 1-x, 2-y, -1+z; *d* = 1-x, 2-y, z.

Supplementary Table 3.15. Selected bond lengths (Å) and bond angles (°) for (**1**) at 7.75 GPa

**Bond Lengths**			
Zn1-Cl1	2.362(6)	Zn1-Cl1^{a}	2.429(6)
Zn1-Cl1^{c}	2.429(6)	Zn1-Cl1^{d}	2.362(6)
Zn1-N1	2.11(2)	Zn1-N1^{d}	2.11(2)
**Bond Angles**			
Cl1-Zn1-N1	92.1(6)	Cl1-Zn1-Cl1^{a}	90.0(2)
Cl1-Zn1-Cl1^{c}	178.4(2)	Cl1-Zn1-Cl1^{d}	91.6(2)
Cl1-Zn1-N1^{d}	91.4(6)	Cl1^{a}-Zn1-N1	88.5(6)
Cl1^{c}-Zn1-N1	87.8(6)	Cl1^{d}-Zn1-N1	91.4(6)
N1-Zn1-N1^{d}	174.9(7)	Cl1^{a}-Zn1-Cl1^{c}	88.4(2)
Cl1^{a}-Zn1-Cl1^{d}	178.4(2)	Cl1^{a}-Zn1-N1^{d}	87.8(6)
Cl1^{c}-Zn1-Cl1^{d}	90.0(2)	Cl1^{c}-Zn1-N1^{d}	88.5(6)
Cl1^{d}-Zn1-N1^{d}	92.1(6)	Zn1-Cl1-Zn1^{b}	90.0(2)

Symmetry code: *a* = x, y, -1+z; *b* = x, y, 1+z; *c* = 1-x, 2-y, -1+z; *d* = 1-x, 2-y, z.

Supplementary Table 3.16. Selected bond lengths (Å) and bond angles (°) for (**1**) at 8.93 GPa

**Bond Lengths**			
Zn1-Cl1	2.338(12)	Zn1-Cl1^{a}	2.421(12)
Zn1-Cl1^{c}	2.421(12)	Zn1-Cl1^{d}	2.338(12)
Zn1-N1	2.12(3)	Zn1-N1^{d}	2.12(3)
**Bond Angles**			
Cl1-Zn1-N1	90.6(7)	Cl1-Zn1-Cl1^{a}	90.0(4)
Cl1-Zn1-Cl1^{c}	178.0(4)	Cl1-Zn1-Cl1^{d}	92.0(4)
Cl1-Zn1-N1^{d}	93.9(7)	Cl1^{a}-Zn1-N1	86.1(7)
Cl1^{c}-Zn1-N1	89.3(7)	Cl1^{d}-Zn1-N1	93.9(7)
N1-Zn1-N1^{d}	173.6(10)	Cl1^{a}-Zn1-Cl1^{c}	88.0(4)
Cl1^{a}-Zn1-Cl1^{d}	178.0(5)	Cl1^{a}-Zn1-N1^{d}	89.3(7)
Cl1^{c}-Zn1-Cl1^{d}	90.0(4)	Cl1^{c}-Zn1-N1^{d}	86.1(7)
Cl1^{d}-Zn1-N1^{d}	90.6(7)	Zn1-Cl1-Zn1^{b}	90.0(5)

Symmetry code: *a* = x, y, 1+z; *b* = x, y, -1+z; *c* = 1-x, 2-y, 1+z; *d* = 1-x, 2-y, z.

Supplementary Table 3.17. Selected bond lengths (Å) and bond angles (°) for (**1**) at 9.34 GPa

**Bond Lengths**			
Zn1-Cl1	2.329(12)	Zn1-Cl1^{a}	2.399(12)
Zn1-Cl1^{c}	2.399(12)	Zn1-Cl1^{d}	2.329(12)
Zn1-N1	2.08(3)	Zn1-N1^{d}	2.08(3)
**Bond Angles**			
Cl1-Zn1-N1	90.3(8)	Cl1-Zn1-Cl1^{a}	90.3(4)
Cl1-Zn1-Cl1^{c}	178.3(4)	Cl1-Zn1-Cl1^{d}	91.4(4)
Cl1-Zn1-N1^{d}	91.6(8)	Cl1^{a}-Zn1-N1	88.4(8)
Cl1^{c}-Zn1-N1	89.6(8)	Cl1^{d}-Zn1-N1	91.6(8)
N1-Zn1-N1^{d}	177.3(11)	Cl1^{a}-Zn1-Cl1^{c}	88.1(4)
Cl1^{a}-Zn1-Cl1^{d}	178.3(4)	Cl1^{a}-Zn1-N1^{d}	89.6(8)
Cl1^{c}-Zn1-Cl1^{d}	90.3(4)	Cl1^{c}-Zn1-N1^{d}	88.4(8)
Cl1^{d}-Zn1-N1^{d}	90.3(8)	Zn1-Cl1-Zn1^{b}	90.3(4)

Symmetry code: *a* = x, y, 1+z; *b* = x, y, -1+z; *c* = 1-x, 2-y, 1+z; *d* = 1-x, 2-y, z.

Supplementary Table 3.18. Selected bond lengths (Å) and bond angles (°) for (**1**) at 7.85 GPa (decompression)

**Bond Lengths**			
Zn1-Cl1	2.347(10)	Zn1-Cl1^a	2.434(10)
Zn1-Cl1^c	2.434(10)	Zn1-Cl1^d	2.347(10)
Zn1-N1	2.10(2)	Zn1-N1^d	2.10(2)
**Bond Angles**			
Cl1-Zn1-N1	90.0(7)	Cl1-Zn1-Cl1^a	89.7(3)
Cl1-Zn1-Cl1^c	177.9(4)	Cl1-Zn1-Cl1^d	92.4(4)
Cl1-Zn1-N1^d	92.0(7)	Cl1^a-Zn1-N1	88.0(7)
Cl1^c-Zn1-N1	89.9(7)	Cl1^d-Zn1-N1	92.0(7)
N1-Zn1-N1^d	177.2(8)	Cl1^a-Zn1-Cl1^c	88.2(4)
Cl1^a-Zn1-Cl1^d	177.9(4)	Cl1^a-Zn1-N1^d	89.9(7)
Cl1^c-Zn1-Cl1^d	89.7(3)	Cl1^c-Zn1-N1^d	88.0(7)
Cl1^d-Zn1-N1^d	90.0(7)	Zn1-Cl1-Zn1^b	89.7(4)

Symmetry code: *a* = x, y, -1+z; *b* = x, y, 1+z; *c* = 2-x, 1-y, 1+z; *d* = 2-x, 1-y, z.

Supplementary Table 3.19. Selected bond lengths (Å) and bond angles (°) for (**1**) at 6.42 GPa (decompression).

**Bond Lengths**			
Zn1-Cl1	2.358(16)	Zn1-Cl1^a	2.416(16)
Zn1-Cl1^c	2.416(16)	Zn1-Cl1^d	2.358(16)
Zn1-N1	2.10(4)	Zn1-N1^d	2.10(4)
**Bond Angles**			
Cl1-Zn1-N1	91.2(11)	Cl1-Zn1-Cl1^a	90.5(5)
Cl1-Zn1-Cl1^c	178.6(6)	Cl1-Zn1-Cl1^d	90.9(6)
Cl1-Zn1-N1^d	91.2(12)	Cl1^a-Zn1-N1	88.7(11)
Cl1^c-Zn1-N1	88.7(11)	Cl1^d-Zn1-N1	91.2(11)
N1-Zn1-N1^d	176.5(17)	Cl1^a-Zn1-Cl1^c	88.1(6)
Cl1^a-Zn1-Cl1^d	178.6(6)	Cl1^a-Zn1-N1^d	88.7(12)
Cl1^c-Zn1-Cl1^d	90.5(5)	Cl1^c-Zn1-N1^d	88.7(12)
Cl1^d-Zn1-N1^d	91.2(12)	Zn1-Cl1-Zn1^b	90.5(5)

Symmetry code: *a* = x, y, -1+z; *b* = x, y, 1+z; *c* = 1-x, -y, -1+z; *d* = 1-x, -y, z.

Supplementary Table 3.20. Selected bond lengths (Å) and bond angles (°) for (**1**) at 4.18 GPa (decompression)

**Bond Lengths**			
Zn1-Cl1	2.413(4)	Zn1-Cl1^{b}	2.413(4)
Zn1-Cl1^{c}	2.413(4)	Zn1-Cl1^{d}	2.413(4)
Zn1-N1	2.147(18)	Zn1-N1^{c}	2.147(18)
**Bond Angles**			
Cl1-Zn1-N1	90.0(4)	Cl1-Zn1-Cl1^{b}	91.77(13)
Cl1-Zn1-Cl1^{c}	88.24(13)	Cl1-Zn1-N1^{c}	90.0(4)
Cl1-Zn1-Cl1^{d}	180.00	Cl1^{b}-Zn1-N1	90.0(4)
Cl1^{c}-Zn1-N1	90.0(4)	N1-Zn1-N1^{c}	180.00
Cl1^{d}-Zn1-N1	90.0(4)	Cl1^{b}-Zn1-Cl1^{c}	180.00
Cl1^{b}-Zn1-N1^{c}	90.0(4)	Cl1^{b}-Zn1-Cl1^{d}	88.24(13)
Cl1^{c}-Zn1-N1^{c}	90.0(4)	Cl1^{c}-Zn1-Cl1^{d}	91.77(13)
Cl1^{d}-Zn1-N1^{c}	90.0(4)	Zn1-Cl1-Zn1^{a}	91.77(18)

Symmetry code: *a* = x, y, -1+z; *b* = x, y, 1+z; *c* = -x, 1-y, z; *d* = -x, 1-y, 1+z.

Supplementary Table 3.21. Selected bond lengths (Å) and bond angles (°) for (**1**) at 3.52 GPa (decompression)

**Bond Lengths**			
Zn1-Cl1	2.408(5)	Zn1-Cl1^{b}	2.408(5)
Zn1-Cl1^{c}	2.408(5)	Zn1-Cl1^{d}	2.408(5)
Zn1-N1	2.11(2)	Zn1-N1^{c}	2.11(2)
**Bond Angles**			
Cl1-Zn1-N1	90.0(5)	Cl1-Zn1-Cl1^{b}	92.41(16)
Cl1-Zn1-Cl1^{c}	87.59(16)	Cl1-Zn1-N1^{c}	90.0(4)
Cl1-Zn1-Cl1^{d}	180.00	Cl1^{b}-Zn1-N1	90.0(5)
Cl1^{c}-Zn1-N1	90.0(5)	N1-Zn1-N1^{c}	180.00
Cl1^{d}-Zn1-N1	90.0(5)	Cl1^{b}-Zn1-Cl1^{c}	180.00
Cl1^{b}-Zn1-N1^{c}	90.0(4)	Cl1^{b}-Zn1-Cl1^{d}	87.59(16)
Cl1^{c}-Zn1-N1^{c}	90.0(4)	Cl1^{c}-Zn1-Cl1^{d}	92.41(16)
Cl1^{d}-Zn1-N1^{c}	90.0(4)	Zn1-Cl1-Zn1^{a}	92.4(2)

Symmetry code: *a* = x, y, -1+z; *b* = x, y, 1+z; *c* = 2-x, 1-y, z; *d* = 2-x, 1-y, 1+z.

Supplementary Table 3.22. Selected bond lengths (Å) and bond angles (°) for (**1**) at 3.09 GPa (decompression)

**Bond Lengths**			
Zn1-Cl1	2.4266(19)	Zn1-Cl1^{b}	2.4266(19)
Zn1-Cl1^{c}	2.4266(19)	Zn1-Cl1^{d}	2.4266(19)
Zn1-N1	2.159(11)	Zn1-N1^{c}	2.159(11)
**Bond Angles**			
Cl1-Zn1-N1	90.0(2)	Cl1-Zn1-Cl1^{b}	92.25(6)
Cl1-Zn1-Cl1^{c}	87.76(6)	Cl1-Zn1-N1^{c}	90.0(2)
Cl1-Zn1-Cl1^{d}	180.00	Cl1^{b}-Zn1-N1	90.0(2)
Cl1^{c}-Zn1-N1	90.0(2)	N1-Zn1-N1^{c}	180.00
Cl1^{d}-Zn1-N1	90.0(2)	Cl1^{b}-Zn1-Cl1^{c}	180.00
Cl1^{b}-Zn1-N1^{c}	90.0(2)	Cl1^{b}-Zn1-Cl1^{d}	87.76(6)
Cl1^{c}-Zn1-N1^{c}	90.0(2)	Cl1^{c}-Zn1-Cl1^{d}	92.25(6)
Cl1^{d}-Zn1-N1^{c}	90.0(2)	Zn1-Cl1-Zn1^{a}	92.25(9)

Symmetry code: *a* = x, y, -1+z; *b* = x, y, 1+z; *c* = 1-x, -y, z; *d* = 1-x, -y, 1+z.

Supplementary Table 3.23. Selected bond lengths (Å) and bond angles (°) for (**1**) at 1.61 GPa (decompression)

**Bond Lengths**			
Zn1-Cl1	2.446(4)	Zn1-Cl1^{b}	2.446(4)
Zn1-Cl1^{c}	2.446(4)	Zn1-Cl1^{d}	2.446(4)
Zn1-N1	2.172(19)	Zn1-N1^{c}	2.172(19)
**Bond Angles**			
Cl1-Zn1-N1	90.0(4)	Cl1-Zn1-Cl1^{b}	93.37(13)
Cl1-Zn1-Cl1^{c}	86.63(13)	Cl1-Zn1-N1^{c}	90.0(4)
Cl1-Zn1-Cl1^{d}	180.00	Cl1^{b}-Zn1-N1	90.0(4)
Cl1^{c}-Zn1-N1	90.0(4)	N1-Zn1-N1^{c}	180.00
Cl1^{d}-Zn1-N1	90.0(4)	Cl1^{b}-Zn1-Cl1^{c}	180.00
Cl1^{b}-Zn1-N1^{c}	90.0(4)	Cl1^{b}-Zn1-Cl1^{d}	86.63(13)
Cl1^{c}-Zn1-N1^{c}	90.0(4)	Cl1^{c}-Zn1-Cl1^{d}	93.37(13)
Cl1^{d}-Zn1-N1^{c}	90.0(4)	Zn1-Cl1-Zn1^{a}	93.37(18)

Symmetry code: *a* = x, y, -1+z; *b* = x, y, 1+z; *c* = 1-x, -y, z; *d* = 1-x, -y, 1+z.

Supplementary Table 3.24. Selected bond lengths (Å) and bond angles (°) for (**1**) at 0.83 GPa (decompression)

**Bond Lengths**			
Zn1-Cl1	2.466(4)	Zn1-Cl1^b	2.466(4)
Zn1-Cl1^c	2.466(4)	Zn1-Cl1^d	2.466(4)
Zn1-N1	2.20(2)	Zn1-N1^c	2.20(2)
**Bond Angles**			
Cl1-Zn1-N1	90.0(4)	Cl1-Zn1-Cl1^b	94.09(13)
Cl1-Zn1-Cl1^c	85.91(13)	Cl1-Zn1-N1^c	90.0(4)
Cl1-Zn1-Cl1^d	180.00	Cl1^b-Zn1-N1	90.0(4)
Cl1^c-Zn1-N1	90.0(4)	N1-Zn1-N1^c	180.00
Cl1^d-Zn1-N1	90.0(4)	Cl1^b-Zn1-Cl1^c	180.00
Cl1^b-Zn1-N1^c	90.0(4)	Cl1^b-Zn1-Cl1^d	85.91(13)
Cl1^c-Zn1-N1^c	90.0(4)	Cl1^c-Zn1-Cl1^d	94.09(13)
Cl1^d-Zn1-N1^c	90.0(4)	Zn1-Cl1-Zn1^a	94.09(19)

Symmetry code: *a* = x, y, -1+z; *b* = x, y, 1+z; *c* = 2-x, 1-y, z; *d* = 2-x, 1-y, 1+z.

Supplementary Table 3.25. Selected bond lengths (Å) and bond angles (°) for (**1**) at 0.40 GPa (decompression)

**Bond Lengths**			
Zn1-Cl1	2.477(3)	Zn1-Cl1^b	2.477(3)
Zn1-Cl1^c	2.477(3)	Zn1-Cl1^d	2.477(3)
Zn1-N1	2.192(14)	Zn1-N1^c	2.192(14)
**Bond Angles**			
Cl1-Zn1-N1	90.0(3)	Cl1-Zn1-Cl1^b	93.94(10)
Cl1-Zn1-Cl1^c	86.06(10)	Cl1-Zn1-N1^c	90.0(3)
Cl1-Zn1-Cl1^d	180.00	Cl1^b-Zn1-N1	90.0(3)
Cl1^c-Zn1-N1	90.0(3)	N1-Zn1-N1^c	180.00
Cl1^d-Zn1-N1	90.0(3)	Cl1^b-Zn1-Cl1^c	180.00
Cl1^b-Zn1-N1^c	90.0(3)	Cl1^b-Zn1-Cl1^d	86.06(10)
Cl1^c-Zn1-N1^c	90.0(3)	Cl1^c-Zn1-Cl1^d	93.94(10)
Cl1^d-Zn1-N1^c	90.0(3)	Zn1-Cl1-Zn1^a	93.94(14)

Symmetry code: *a* = x, y, -1+z; *b* = x, y, 1+z; *c* = -x, 1-y, z; *d* = -x, 1-y, 1+z.

The different symmetries in the low- and high-pressure phases were visualized using the Mercury software, Supplementary Figure 3.3. It is immediately clear that the C_2 axes which run along the N-Zn-N molecular axes are lost over the phase transition. This is the result of the 'butterfly' puckering motion displayed in Figure 2 in the main manuscript.

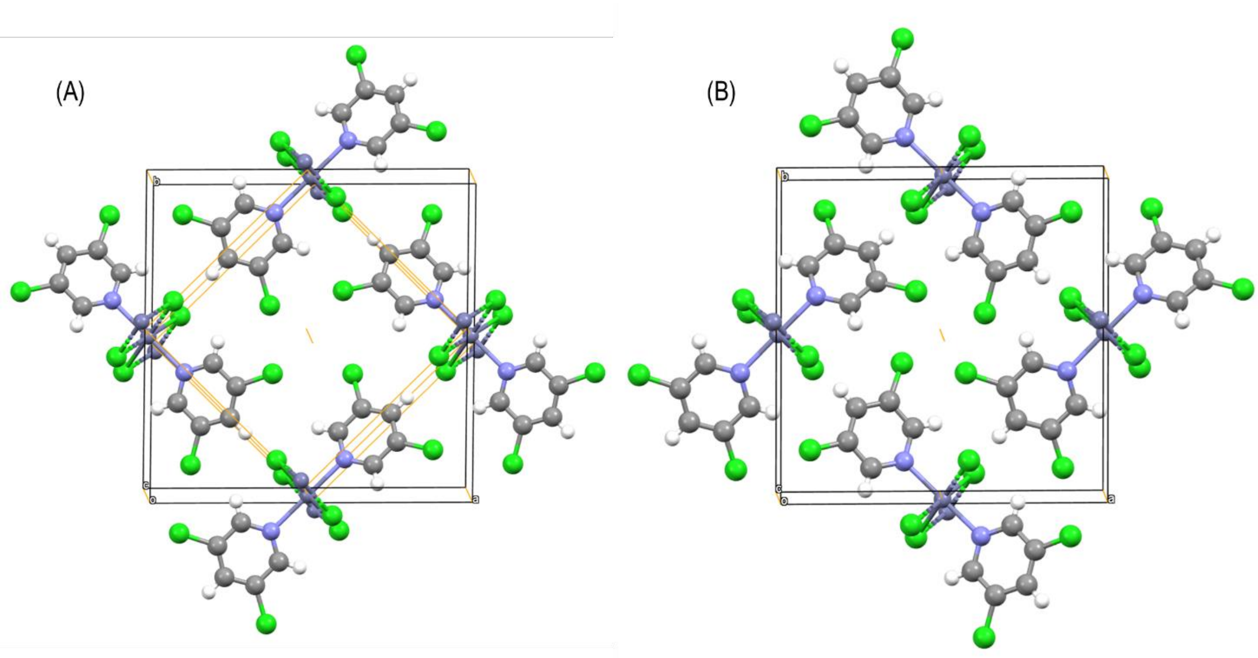

Supplementary Figure 3.3: Location of C_2 rotational axes within the low- and high-pressure structures of (**1**). The symmetry axes are identified by orange lines. Atoms are labelled as (green) Cl, (blue) N, (grey) C, and (white) H.

Supplementary Note 3.2| Computational High-Pressure Structures

The high-pressure response of (**1**) was explored by *ab initio* density functional theory calculations using the PBE-TS scheme (see Supplementary Note 2). Validation of the selected model was made by comparing the simulated lattice parameters against those of the experimentally reported structure under ambient conditions.[18] The crystallographic *a* and *b* axes are overestimated by only 0.4% by PBE-TS simulations, whereas the *c* axis (i.e. the covalently bound CP chains) is overestimated by 1.12%. Overall, our PBE-TS simulations overestimate the unit cell volume by < 2%, suggesting the model to provide a good representation of the experimental structure. The structure was modelled to elevated pressures in increments of 0.5 GPa, Supplementary Table 3.26, showing monotonic compression up to 8 GPa. The simulated high-pressure values are in excellent agreement with experimentally measured unit cell parameters (see e.g. Supplementary Table 3.1 and Figure 2 in the main text)

Supplementary Table 3.26: PBE-TS simulated high pressure unit cell geometry for (**1**). The initial structure is taken from the ambient pressure experimental geometry ($P\bar{4}b2$). For lattice parameters at p=0 GPa, comparison is given with the ambient experimental values (see Supplementary Table 3.1) in parentheses.

p/GPa	*a* /Å	*b* /Å	*c* /Å	V /Å^3
0	13.872 (+0.4%)	13.872 (+0.4%)	3.681 (+1.12%)	710.421 (+1.86%)
0.5	13.725	13.725	3.662	689.832
1.0	13.604	13.604	3.636	672.910
1.5	13.507	13.507	3.612	658.970
2.0	13.418	13.418	3.591	646.533
2.5	13.338	13.338	3.570	635.111
3.0	13.267	13.267	3.551	625.023
3.5	13.202	13.202	3.533	615.776
4.0	13.144	13.144	3.516	607.441
4.5	13.099	13.099	3.500	600.543
5.0	13.041	13.041	3.484	592.516
5.5	12.996	12.996	3.469	585.900
6.0	12.954	12.954	3.454	579.602
6.5	12.915	12.915	3.440	573.649
7.0	12.879	12.879	3.426	568.266
7.5	12.844	12.844	3.413	563.037
8.0	12.812	12.812	3.400	558.101

Selected molecular geometric parameters were extracted from the high pressure simulated structures, Supplementary Table 3.27. In good agreement with the experimentally determined geometries (see Supplementary Table 3.2), the Zn…Cl and Zn…N bonds are all found to compress with pressure, whereas the ∡Cl-Zn-Cl increases with pressure. The close agreement between experiment and theory further validates our choice of model.

Supplementary Table 3.27: PBE-TS simulated high pressure molecular geometry for (**1**). The initial structure is taken from the ambient pressure experimental geometry ($P\bar{4}b2$). Selected bond distances [r(A...B)] and [∡A-B-C] are given.

p/GPa	r(Zn…Cl_1) /Å	r(Zn…Cl_2) /Å	r(Zn…N) /Å	∡Cl-Zn-Cl /°	∡Zn-Cl-Zn /°
0	2.513	2.513	2.206	85.48	94.52
0.5	2.502	2.502	2.195	85.91	94.09
1.0	2.492	2.492	2.183	86.29	93.71
1.5	2.482	2.482	2.174	86.63	93.37
2.0	2.474	2.474	2.165	86.93	93.07
2.5	2.465	2.465	2.156	87.19	92.81
3.0	2.457	2.457	2.148	87.44	92.56
3.5	2.449	2.449	2.141	87.65	92.35
4.0	2.441	2.441	2.134	87.88	92.12
4.5	2.434	2.434	2.127	88.07	91.93
5.0	2.427	2.427	2.120	88.28	91.72
5.5	2.421	2.421	2.114	88.49	91.51
6.0	2.415	2.415	2.109	88.67	91.33
6.5	2.409	2.409	2.103	88.87	91.13
7.0	2.403	2.403	2.098	89.06	90.94
7.5	2.398	2.398	2.093	89.25	90.75
8.0	2.392	2.392	20.88	89.43	90.57

Similarly, we explored the high-pressure response of (**1**), starting from the high-pressure geometry, $P\bar{4}$, Supplementary Table 3.28. As $P\bar{4}$ is a subgroup of $P\bar{4}b2$, the full symmetry of the high-pressure structure can be captured by either geometry. Reassuringly, as the $P\bar{4}$ structure was relaxed under reduced pressure, it re-adopted the geometry of $P\bar{4}b2$. The unit cell geometry captured by DFT optimization across the pressure range corresponds well to the high-pressure behavior observed from simulation of the $P\bar{4}b2$ structure, Supplementary Table 3.28 and Supplementary Figure 3.4.

Supplementary Table 3.28: PBE-TS simulated high pressure unit cell geometry for (**1**). The initial structure is taken from the high-pressure experimental geometry ($P\bar{4}$). For lattice parameters at p=0 GPa, comparison is given with the ambient experimental values (see Supplementary Table 3.1) in parentheses.

p/GPa	*a* /Å	*b* /Å	*c* /Å	V /Å^3
0	13.873 (+0.4%)	13.873 (+0.4%)	3.681 (+1.12%)	710.543 (+1.86%)
1.0	13.608	13.608	3.637	673.491
2.0	13.419	13.419	3.591	646.630
3.0	13.268	13.268	3.551	625.117
4.0	13.144	13.144	3.516	607.441
5.0	13.041	13.041	3.484	592.516
6.0	12.953	12.953	3.454	579.513
7.0	12.869	12.869	3.431	567.352
8.0	12.781	12.785	3.407	556.920

Selected molecular geometric parameters were extracted from the high pressure simulated structures, Supplementary Table 3.29. These values are in good agreement with both the experimentally determined geometries (see Supplementary Table 3.2), and with the geometries obtained through high pressure simulations starting with the ambient pressure phase (see Supplementary Table 3.27).

Supplementary Table 3.29: PBE-TS simulated high pressure molecular geometry for (**1**). The initial structure is taken from the high-pressure experimental geometry ($P\bar{4}$). Selected bond distances [r(A…B)] and [∡A-B-C] are given. Note, symmetry breaking is observed above 5 GPa.

p/Gpa	r(Zn…Cl_1) /Å	r(Zn…Cl_2) /Å	r(Zn…N) /Å	∡Cl-Zn-Cl /º	∡Zn-Cl-Zn /º	∡Cl-Zn-Cl /º	∡Zn-Cl-Zn /º
0	2.513	2.513	2.207	85.47	94.53	85.48	94.53
1.0	2.492	2.492	2.184	86.28	93.72	86.29	93.72
2.0	2.474	2.474	2.165	86.93	93.07	86.93	93.07
3.0	2.457	2.457	2.148	87.44	92.56	87.44	92.56
4.0	2.441	2.441	2.134	87.88	92.13	87.87	92.13
5.0	2.424	2.432	2.121	88.46	91.72	88.10	91.72
6.0	2.411	2.419	2.109	88.84	91.33	88.50	91.33
7.0	2.389	2.424	2.099	88.22	90.95	89.88	90.95
8.0	2.377	2.417	2.089	88.48	90.57	90.37	90.57

The simulated pressure-volume curves for (**1**) starting from the high- and low-pressure phases is plotted in Supplementary Figure 3.4. No significant differences are observed, showing that the phase transition is predictable and reversible also through our DFT-TS simulations. The phase transition is captured at the atomistic level when symmetry restrictions are reduced and optimization of the pressure-volume curve is conducted at the lower symmetry, $P\bar{4}$, e.g. as indicated by the symmetry breaking in the geometry as tabulated in Supplementary Table 3.29.

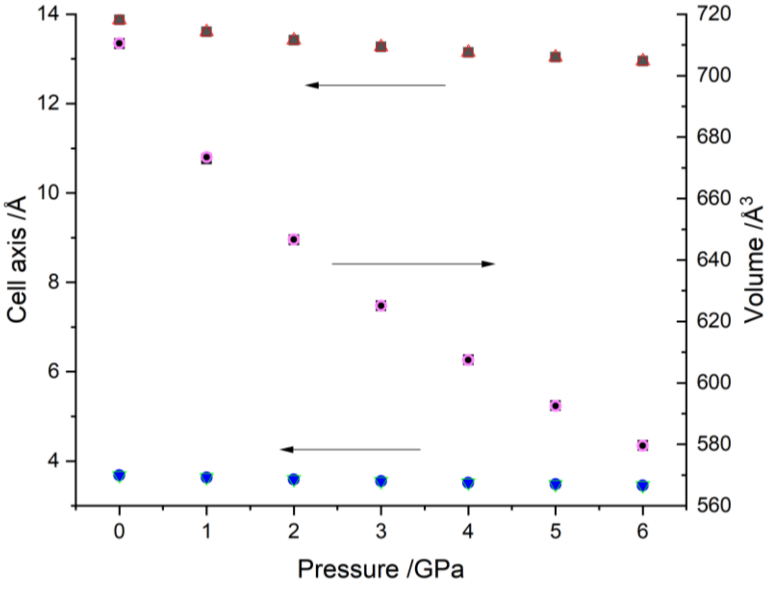

Supplementary Figure 3.4: Comparison of unit cell geometry obtained from PBE-TS simulations based on the ambient pressure (filled symbols) and high-pressure (open symbols) unit cells.

Supplementary Note 3.3| Equation of State

The isothermal equation of state (EoS) for (**1**) was obtained by fitting the volume-pressure data to established empirical equations, namely the 3rd order Birch-Murnaghan[19] and Vinet[20] EoS, Supplementary Equations 3.1 and 3.2, respectively.

$$p(V) = \frac{3}{2}B\left[\left(\frac{V_0}{V}\right)^{\frac{7}{3}} - \left(\frac{V_0}{V}\right)^{\frac{5}{3}}\right]\left[1 + \frac{3}{4}(B' - 4)\left(\frac{V_0}{V}\right)^{\frac{2}{3}} - 1\right] \quad \textbf{Eqn 3.1}$$

$$p(V) = 3B\left(1 - \frac{\left(\frac{V}{V_0}\right)^{\frac{1}{3}}}{\left(\frac{V}{V_0}\right)^{\frac{1}{9}}}\right)\exp\left(\frac{3}{2}(B' - 1)\left(1 - \left(\frac{V}{V_0}\right)^{\frac{1}{3}}\right)\right) \quad \textbf{Eqn 3.2}$$

Experimental and theoretical high-pressure data were fit to these equations using in house software *via* BFGS minimization. The ambient pressure volume (V_0) was allowed to vary during minimization and its agreement with experimental values was taken as indicative of the fit quality. Both the bulk modulus (B) and its derivative (B') are reported in Supplementary Table 3.30-3.31. Fits to Supplementary Equations 3.1 and 3.2 were performed using the complete dataset, and by separating the data set into the low- and high-pressure forms for both experimental and simulated data, Supplementary Figure 3.5-3.6, respectively.

Supplementary Table 3.30: Parameters obtained for fits to high pressure data for experimental (Exp) and simulation (Comp) data at the PBE-TS level of theory. Values correspond to fits against the whole dataset.

		B /GPa^{-1}	B'	V_0 (exp)	V_0 (fit)
Exp.	BM3	14.52(8)	7.418(3)	697.43	694.04(7)
	Vinet	14.41(8)	7.355(2)	697.43	694.38(8)
Comp.	BM3	17.01	5.597	708.45	708.39
	Vinet	16.77	5.872	708.45	708.53

Supplementary Table 3.31: Parameters obtained for fits to high pressure data for experimental (Exp) and simulation (Comp) data at the PBE-TS level of theory. Values correspond to fits against data up to 5 GPa.

		B /GPa^{-1}	B'	V_0 (exp)	V_0 (fit)
Exp.	BM3	13.00(2)	9.83(1)	697.43	694.59(1)
	Vinet	13.55(5)	8.77(5)	697.43	694.08(9)
Comp.	BM3	16.80	5.678	708.45	708.70
	Vinet	16.68	5.875	708.45	708.75

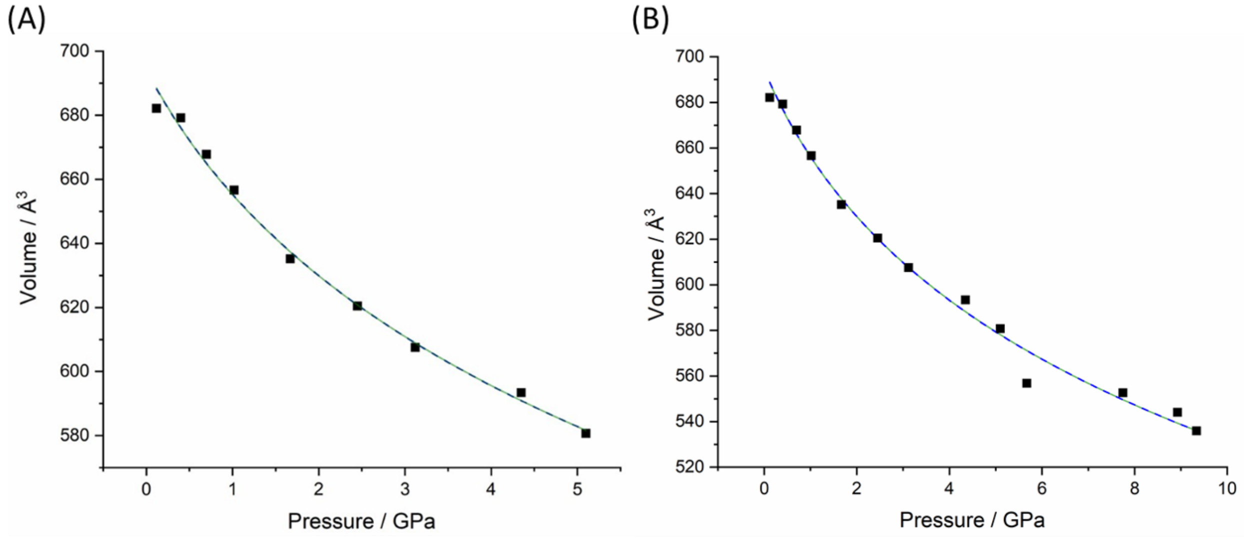

Supplementary Figure 3.5: EoS fits to volume-pressure data for the experimental compression of (**1**). Fits according to the (green) 3rd order Birch-Murnaghan (Supplementary Eqn 3.1) and (blue) Vinet (Supplementary Eqn 3.2) EoS formalisms are shown. (A) Fits to low pressure data; (B) Fit entire pressure range.

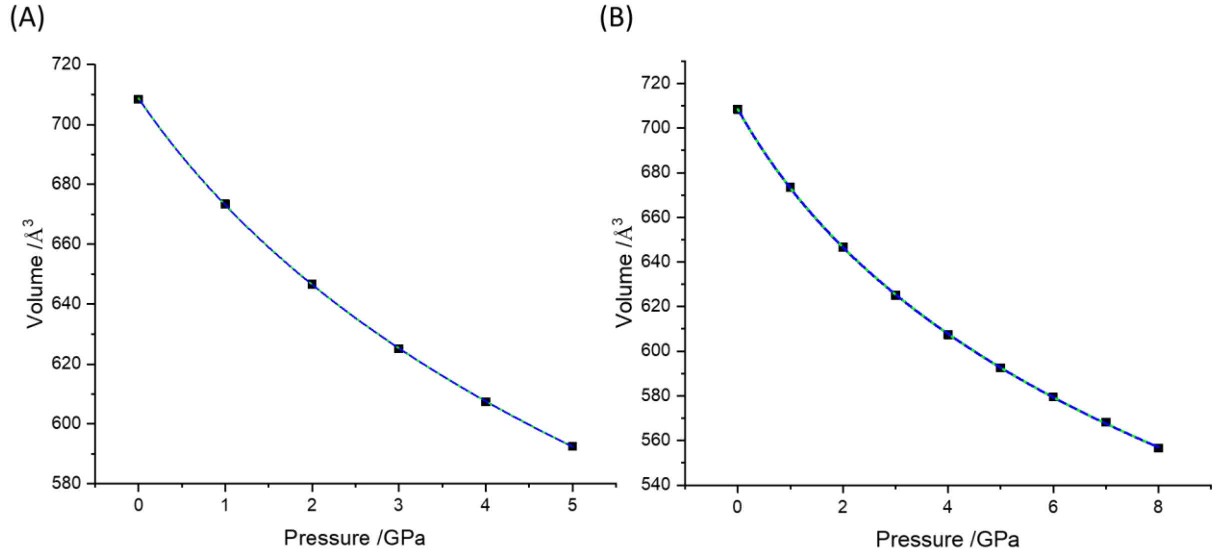

Supplementary Figure 3.6: EoS fits to volume-pressure data for the PBE-TS simulated compression of (**1**). Fits according to the (green) 3rd order Birch-Murnaghan (Supplementary Eqn 3.1) and (blue) Vinet (Supplementary Eqn 3.2) EoS formalisms are shown. (A) Fits to low pressure data; (B) Fit entire simulated dataset.

Supplementary Note 3.4| High Pressure Electronic Structure

The electronic band structure was calculated for the experimentally determined geometries of (**1**), Supplementary Figure 3.7. For expletive purposes we show the band structures at 0.12 GPa, 4.35 GPa and 5.67 GPa. The former two provide a comparison of low- and high-pressure structures with the same space group symmetry, whereas the latter highlights the effects of the phase transition on the electronic structure. Across all three pressure points, only minor differences are observed, with no significant change in the PBE-TS predicted band gap of approximately 3 eV. Analysis of the crystal orbital Hamilton populations (COHP) suggests also that pressure has no significant influence on the nature of Zn..Cl bonds which coordination polymer chains, aside from a slight increase in their covalent strength (see integrated ICOHP, Supplementary Table 3.32).

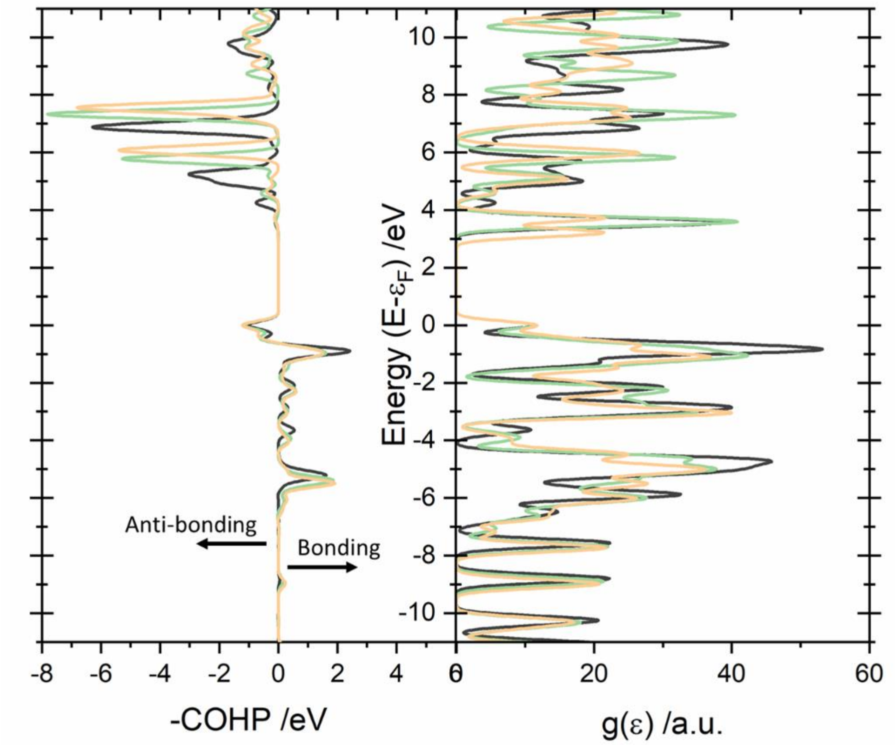

Supplementary Figure 3.7: Electronic structure of (**1**) at ambient and elevated pressures. The electronic band structures correspond to PBE-TS level at the experimentally determined unit cell volumes and atomic positions. (Right) The full band structure is provided for (black) the $P\bar{4}b2$ symmetry structure at 0.12 GPa, (green) the $P\bar{4}b2$ symmetry structure at 4.35 GPa, and (orange) the $P\bar{4}$ symmetry structure at 5.67 GPa. (Left) The crystal orbital Hamilton populations (COHP) is provided for the Zn..Cl covalent bonds in the CP chain of (**1**) at (black) 0.12 GPa, (green) 4.35 GPa, and (orange) 5.67 GPa.

Supplementary Table 3.32: Integrated COHP values up to the Fermi level (ϵ_F) for the Zn..Cl interactions in the CP chains at representative pressures.

	0.12 GPa	4.35 GPa	5.67 GPa
Zn-Cl_1	-6.82	-7.54	-7.79
Zn-Cl_2	-6.83	-7.567	-7.80

Supplementary Note 3.5| Effects of Pressure on Short Contacts

The inter-chain Cl…Cl interactions were measured with increasing pressure, Supplementary Figure 3.8. The Cl…Cl interactions were found to compress continuously up to the phase transition (*ca.* 5 GPa) at which point a marked discontinuity of compressibility is observed. This discontinuity reflects the nature of the phase transition, which involves the change in bipyridyl ligan geometry. The trend in Cl…Cl interaction distance is reflected closely during decompression, suggesting no detectable hysteresis in decompression. Similarly, the H…Cl interactions between the pyridyl H atom and backbone Cl atoms compress quasi-continuously until the phase transition point, at which point symmetry splitting occurs and the two H…Cl interactions become asymmetric. In contrast, we find no discontinuity in the compressibility of the Zn…Zn internuclear distances, consistent with the continuity of compressibility in the unit cell axes (see Supplementary Figure 3.5)

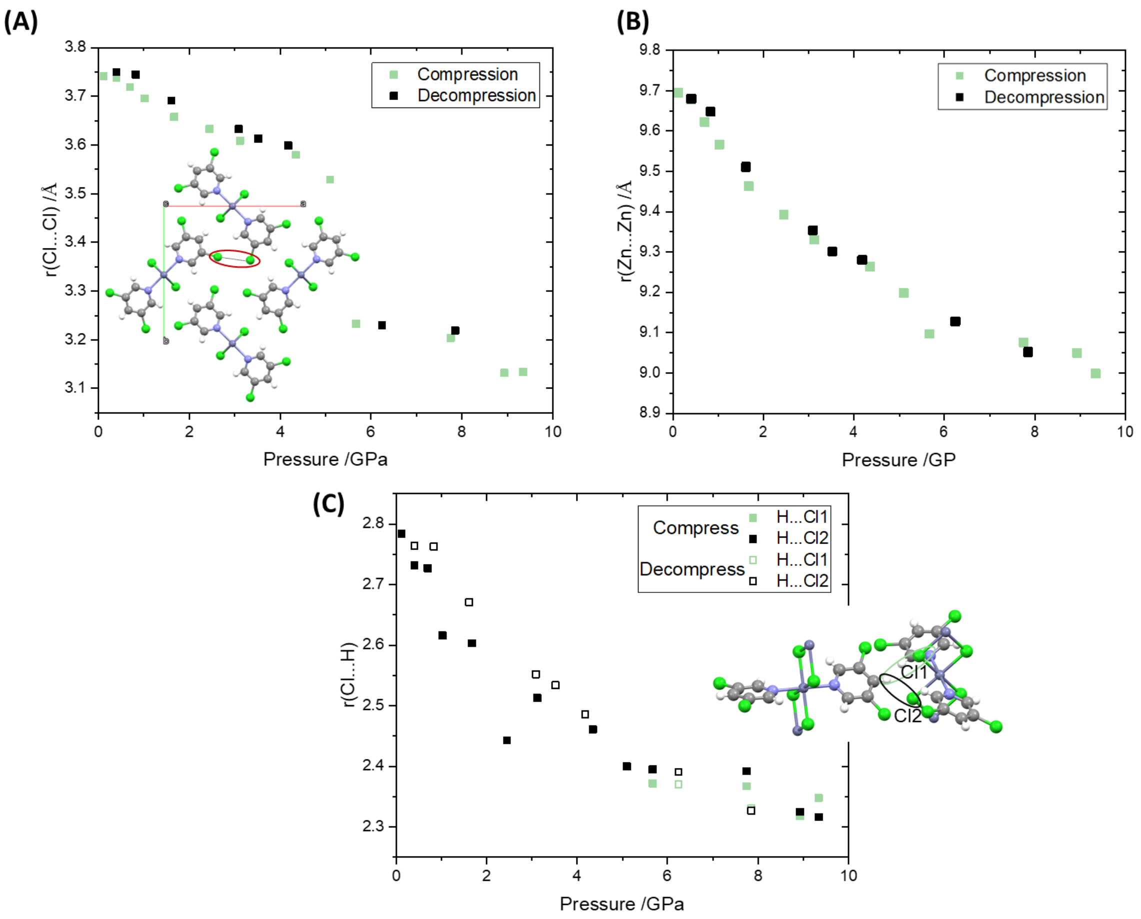

Supplementary Figure 3.8: The effects of pressure on short contacts in (1) upon compression (green) and decompression (black). (A) Compressibility of inter-chain Cl…Cl interactions and (B) compression of the Zn…Zn distances. (C) The effects of pressure on the two H…Cl interactions upon (filled) compression and (empty) decompression, showing symmetry splitting at the phase transition.

Supplementary Note 3.6| Comparison to Low Temperature Crystallography

It is often expected that high pressure structures evolve similarly to low temperature structures. For comparison with the compressibility of (1) we therefore explored the unit cell contraction upon cooling to 100 K, Supplementary Figure 3.9. The relative contraction of the *a/b* axes is not equivalent to that of the *c* axis, leading to a non-spherical thermal expansion indicatrix. This contrasts markedly the high-pressure response of (1) (see Figure 2 in the main manuscript).

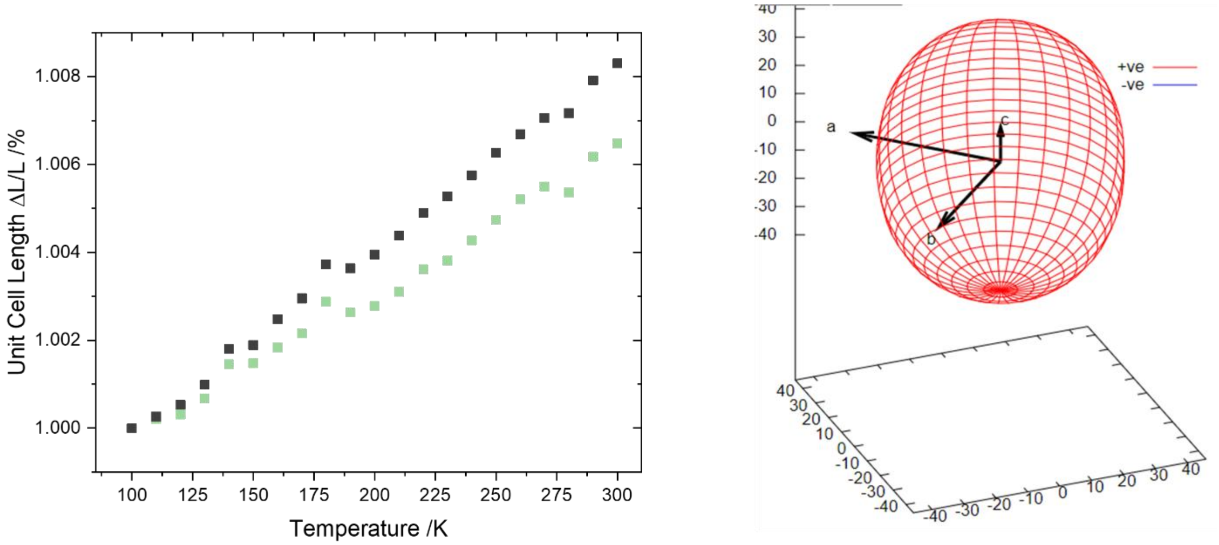

Supplementary Figure 3.9: Thermal expansion of a single crystal of (**1**). (Left) The relative expansion of the *a*=*b* (black) and *c* (green) unit cell axes. (Right) the corresponding thermal expansion indicatrix.[21]

Supplementary Note 4| Theoretical Vibrational Analysis

Supplementary Note 4.1| Simulated Vibrational Spectra at Elevated Pressures

CP (**1**) crystallizes in space group $P\bar{4}b2$, point group $D_{2\mathrm{h}}$ ($-42m$). In this point group, normal modes with symmetries A_1, B_1, B_2, and E are Raman active. *Via* group symmetry analysis it is found that the primitive unit cell of (**1**) contains a total of 150 normal modes, comprising $\Gamma_{acoustic} = B_2 + E$ and $\Gamma_{optical} = 16A_1 + 21A_2 + 16B_1 + 37E$. The simulated vibrational frequencies for (**1**) based on the $P\bar{4}b2$ geometry are given in Supplementary Table 4.1-4.3.

Supplementary Table 4.1: PBE-TS simulated vibrational frequencies for (**1**) from 0 to 2 GPa. The irreducible representation (IR) is shown in each case.

#	IR (0 GPa)	$\boldsymbol{\nu}$(0)	IR (0.5 GPa)	$\boldsymbol{\nu}$(0.5)	IR (1.0 GPa)	$\boldsymbol{\nu}$(1.0)	IR (1.5 GPa)	$\boldsymbol{\nu}$(1.5)	IR (2.0 GPa)	$\boldsymbol{\nu}$(2.0)
4	A_2	25.2	A_2	23.7	A_2	22.0	A_2	20.3	A_2	17.8
5	B_2	27.8	B_2	29.2	B_2	30.3	B_2	30.3	B_2	30.1
6	E	37.3	E	42.0	E	45.5	E	48.0	E	50.2
7	A_2	39.9	A_2	45.4	A_2	48.5	A_2	51.5	A_2	52.8
8	B_2	45.9	B_1	49.5	B_1	51.7	B_1	53.3	B_1	54.6
9	B_1	46.7	B_2	51.2	B_2	55.7	E	58.6	E	59.9
10	E	50.8	E	54.5	E	56.9	B_2	59.5	B_2	63.0
11	E	57.6	A_2	63.0	A_2	67.2	E	70.5	E	72.8
12	A_2	57.7	E	63.5	E	67.4	A_2	72.3	A_2	74.8
13	A_1	64.6	A_1	70.0	A_1	74.0	A1	77.0	A_1	79.7
14	E	65.8	E	71.7	E	76.0	E	79.5	E	82.4
15	A_1	80.0	A_1	84.2	A_1	88.1	A_1	91.2	A_1	94.0
16	E	93.3	A_2	102.1	A_2	104.5	A_2	106.0	A_2	107.4
17	A_2	96.1	B_2	102.3	B_2	105.8	B_2	108.3	B_2	110.0
18	B_2	96.3	A_2	102.5	A_2	107.6	A_2	112.0	A_2	116.1
19	B_1	98.4	E	103.4	B_2	110.6	B_2	115.0	B_2	119.1
20	A_2	98.9	B_2	105.3	E	111.7	B_1	116.3	B_1	120.3
21	B_2	100.1	B_1	105.7	B_1	111.8	E	118.2	E	121.5
22	E	117.7	E	119.4	E	120.8	E	122.2	E	125.8
23	E	134.6	E	137.1	E	139.4	E	141.5	E	143.4
24	B_2	143.6	B_2	145.8	B_2	148.0	B_2	150.3	B_2	152.5
25	A_2	144.6	A_2	147.7	A_2	150.6	A_2	153.0	A_2	155.8
26	E	144.9	E	150.6	E	155.2	E	158.6	E	162.1
27	A_2	157.4	A_2	161.0	A_2	164.2	A_2	167.0	A_2	169.9
28	B_2	161.4	B_2	166.2	E	169.9	E	171.9	E	173.6
29	E	165.6	E	168.0	B_2	170.7	B_2	174.9	B_2	178.5
30	E	184.7	E	188.8	B_2	192.6	B_2	196.4	B_2	200.8
31	B_2	186.0	B_2	189.3	E	193.0	E	197.3	E	202.2
32	A_2	188.2	A_2	193.6	A_2	199.2	A_1	204.7	A_1	207.1
33	A_1	195.1	A_1	198.8	A_1	201.9	A_2	205.0	B_1	207.5
34	B_1	196.3	B_1	199.7	B_1	202.5	B_1	205.3	A_2	211.2
35	E	197.3	E	203.1	E	207.9	E	212.5	E	216.7
36	E	211.8	E	214.2	E	216.4	E	218.6	E	221.1
37	A_1	214.2	A_1	216.0	A_1	217.7	A_1	219.5	A_1	221.6
38	B_1	214.3	B_1	217.0	B1	219.3	B_1	221.8	B_1	224.4
39	B_1	219.0	B_1	221.5	B_1	224.2	B_1	226.9	B_1	229.7
40	E	223.9	E	227.6	E	231.3	E	234.7	E	238.2
41	A_1	225.3	A_1	229.7	A_1	234.0	A_1	238.4	A_1	242.6
42	A_2	380.7	A_2	381.5	A_2	382.2	A_2	382.8	A_2	383.5
43	B_2	380.9	B_2	381.8	B_2	382.5	B_2	383.0	B_2	383.5
44	E	384.1	E	384.9	E	385.5	E	385.9	E	386.3
45	A_1	390.5	A_1	391.9	A_1	393.1	A_1	394.1	A_1	394.9

46	E	392.3	B_1	393.6	B_1	394.7	B_1	395.6	B_1	396.4
47	B_1	392.4	E	393.7	E	395.0	E	396.1	E	397.0
48	B_2	437.8	B_2	438.5	B_2	439.0	B_2	439.4	B_2	439.8
49	E	439.2	E	439.7	E	440.1	E	440.4	E	440.7
50	A_2	440.1	A_2	440.6	A_2	440.9	A_2	441.4	A_2	441.7
51	A_2	459.9	A_2	459.3	A_2	458.6	A_2	458.1	A_2	457.7
52	E	460.0	E	459.4	E	458.8	E	458.3	E	458.0
53	B_2	460.7	B_2	460.2	B_2	459.7	B_2	459.3	B_2	459.1
54	E	518.4	E	517.9	E	517.4	A_1	516.9	A_1	516.5
55	A_1	518.6	A_1	518.0	A_1	517.4	E	517.0	E	516.7
56	B_1	518.9	B_1	518.5	B_1	518.1	B_1	517.8	B_1	517.5
57	B_2	673.6	B_2	673.0	B_2	672.5	B_2	672.3	B_2	672.3
58	E	675.2	E	674.6	E	674.0	E	673.8	E	673.7
59	A_2	676.2	A_2	675.6	A_2	675.1	A_2	674.7	A_2	674.7
60	A_1	676.9	A_1	679.2	A_1	681.3	A_1	683.2	A_1	685.2
61	B_1	679.3	B_1	681.8	B_1	684.1	B_1	686.2	B_1	688.4
62	E	679.7	E	682.1	E	684.5	E	686.7	E	688.9
63	B_2	807.6	B_2	808.5	B_2	809.5	B_2	810.4	B_2	811.1
64	E	811.3	E	812.2	E	813.2	E	814.0	E	814.7
65	A_2	816.2	A_2	817.0	A_2	817.8	A_2	818.6	A_2	819.1
66	B_2	868.1	B_2	866.3	B_2	864.4	B_2	862.8	B_2	861.2
67	E	869.9	E	868.0	E	866.0	E	864.1	E	862.3
68	A_2	870.8	A_2	868.9	A_2	866.9	A_2	865.0	A_2	863.1
69	A_2	891.2	A_2	889.1	A_2	887.4	A_2	886.2	A_2	885.6
70	E	893.7	E	891.7	E	890.0	E	888.8	B_2	887.9
71	B_2	894.5	B_2	892.4	B_2	890.3	B_2	888.9	E	888.1
72	E	913.0	E	912.4	E	911.8	E	911.7	E	911.9
73	A_1	914.6	A_1	914.0	A_1	913.5	A_1	913.4	A_1	913.7
74	B_1	914.8	B_1	914.2	B_1	913.7	B_1	913.6	B_1	913.8
75	B_1	1009.2	B_1	1010.5	B_1	1011.8	B_1	1012.9	B_1	1014.0
76	E	1010.5	E	1012.0	E	1013.3	E	1014.5	E	1015.7
77	A_1	1014.7	A_1	1016.2	A_1	1017.6	A_1	1018.9	A_1	1020.1
78	A_2	1094.0	A_2	1095.4	A_2	1096.5	A_2	1097.6	A_2	1098.4
79	E	1097.3	E	1099.0	E	1100.3	E	1101.2	E	1102.1
80	B_2	1098.1	B_2	1100.0	B_2	1101.3	B_2	1102.3	B_2	1103.1
81	E	1103.0	E	1105.1	E	1107.2	E	1109.1	E	1110.9
82	B1	1105.8	B_1	1107.8	B_1	1109.9	B_1	1111.7	B_1	1113.5
83	A_1	1110.7	A_1	1112.9	A_1	1115.2	A_1	1117.2	A_1	1119.1
84	A_1	1163.1	A_1	1164.0	A_1	1164.7	A_1	1165.2	A_1	1165.6
85	E	1163.8	E	1164.9	E	1165.7	E	1166.2	E	1166.8
86	B_1	1166.7	B_1	1168.0	B_1	1169.0	B_1	1169.7	B_1	1170.4
87	B_2	1269.9	B_2	1271.5	B_2	1272.6	B_2	1273.4	B_2	1273.9
88	E	1273.2	E	1275.3	E	1276.8	E	1277.8	A_2	1278.4
89	A_2	1273.5	A_2	1275.6	A_2	1277.0	A_2	1277.8	E	1278.6
90	B_2	1301.6	B_2	1304.8	B_2	1308.0	B_2	1311.2	A_2	1314.4
91	A_2	1302.3	A_2	1305.4	A_2	1308.4	A_2	1311.4	B_2	1314.4
92	E	1303.1	E	1306.1	E	1309.1	E	1312.1	E	1315.1
93	B_2	1401.9	B_2	1403.6	B_2	1405.0	B_2	1406.2	B_2	1407.4
94	E	1403.8	E	1405.3	E	1406.7	E	1407.9	E	1409.1
95	E	1406.4	E	1407.8	E	1409.0	B_1	1410.0	B_1	1410.9
96	B_1	1406.7	B_1	1407.9	B_1	1409.0	E	1410.1	E	1411.2
97	A_2	1407.4	A_1	1409.2	A_1	1410.4	A_1	1411.5	A_1	1412.5
98	A_1	1407.9	A_2	1409.3	A_2	1411.0	A_2	1412.4	A_2	1413.9
99	B_2	1537.8	B_2	1540.0	B_2	1542.1	B_2	1544.0	B_2	1545.9
100	E	1539.8	E	1542.0	E	1544.1	E	1546.1	E	1548.1
101	A_2	1541.4	A_2	1543.6	A_2	1545.6	A_2	1547.6	A_2	1549.5
102	B_1	1554.9	B_1	1557.9	B_1	1560.7	B_1	1563.3	B_1	1565.9
103	E	1555.5	E	1558.5	E	1561.3	E	1564.0	E	1566.6
104	A_1	1557.2	A_1	1560.3	A_1	1563.2	A_1	1565.9	A_1	1568.6
105	A_1	3132.1	E	3138.4	E	3142.4	E	3145.0	A_2	3147.4

106	E	3132.1	A_1	3138.6	B_2	3142.6	B_2	3145.1	E	3147.4
107	B_1	3133.1	B_2	3139.6	A_2	3142.6	A_2	3145.1	B_2	3147.4
108	B_2	3136.0	A_2	3139.6	A_1	3144.5	A_1	3147.8	A_1	3150.3
109	A_2	3136.0	B_1	3139.6	B_1	3145.0	E	3148.0	E	3150.4
110	E	3136.3	E	3140.4	E	3145.5	B_1	3148.2	B_1	3150.8
111	E	3138.8	E	3142.4	E	3146.3	E	3152.8	E	3159.5
112	A_1	3139.3	A_1	3143.1	A_1	3147.2	A_1	3153.1	A_1	3159.6
113	B_1	3139.9	B_1	3144.0	B_1	3148.8	B_1	3154.9	B_1	3161.6

Supplementary Table 4.2: PBE-ts simulated vibrational frequencies for (**1**) from 2.5 to 4.5 GPa. The irreducible representation (IR) is shown in each case.

#	IR (2.5 GPa)	$\boldsymbol{\nu}$(2.5)	IR (3.0 GPa)	$\boldsymbol{\nu}$(3.0)	IR (3.5 GPa)	$\boldsymbol{\nu}$(3.5)	IR (4.0 GPa)	$\boldsymbol{\nu}$(4.0)	IR (4.5 GPa)	$\boldsymbol{\nu}$(4.5)
4	A_2	15.7	A_2	13.1	A_2	9.7	A_2	4.9	A_2	-6.7
5	B_2	30.2	B_2	30.7	B_2	31.3	B_2	31.8	B_2	31.4
6	E	52.1	E	54.4	E	56.6	A_2	58.8	A_2	60.7
7	A_2	54.1	A_2	55.6	A_2	57.4	E	58.9	B_1	61.0
8	B_1	55.7	B_1	57.0	B_1	58.3	B_1	59.7	E	61.2
9	E	61.1	E	62.5	E	63.8	E	65.4	E	66.8
10	B_2	66.2	B_2	69.2	B_2	71.9	B_2	74.5	B_2	76.7
11	E	74.8	E	77.0	E	78.8	E	80.1	E	81.8
12	A_2	77.5	A_2	80.1	A_2	82.6	A_2	85.1	A_2	87.3
13	A_1	82.2	A_1	84.6	A_1	87.1	A_1	89.6	A_1	91.9
14	E	85.3	E	88.4	E	91.0	E	93.3	E	95.7
15	A_1	96.6	A_1	99.1	A_1	101.4	A_1	103.8	A_1	105.9
16	A2	108.8	A_2	110.2	A_2	111.7	A_2	113.2	A_2	114.5
17	B_2	111.5	B_2	112.9	B_2	114.3	B_2	115.7	B_2	117.1
18	A_2	119.8	A_2	123.2	E	125.2	E	126.3	E	127.4
19	B_2	122.6	E	124.1	A_2	126.3	A_2	129.0	A_2	131.7
20	E	122.9	B_2	125.6	B_2	128.2	B_2	130.6	B_2	132.7
21	B_1	123.6	B_1	126.9	B_1	130.0	B_1	133.9	B1	137.4
22	E	130.5	E	134.8	E	138.3	E	141.2	E	143.5
23	E	145.2	E	147.1	E	149.0	E	151.0	E	152.8
24	B_2	154.6	B_2	156.6	B_2	158.5	B_2	160.4	B_2	162.0
25	A_2	158.3	A2	160.4	A_2	162.5	A_2	163.7	A_2	165.4
26	E	165.4	E	168.5	E	171.2	E	173.6	E	175.9
27	A_2	172.6	A_2	175.2	A_2	177.7	A_2	180.1	E	182.0
28	E	175.2	E	177.2	E	178.8	E	180.4	A_2	182.4
29	B_2	181.9	B_2	185.0	B_2	187.9	B_2	190.6	B_2	193.1
30	B_2	205.7	B_2	211.1	A_1	213.5	A_1	215.9	A_1	217.7
31	E	207.2	A_1	211.5	B_1	214.2	B_1	216.1	B_1	217.8
32	A_1	209.4	B_1	212.1	B_2	216.6	B_2	222.0	E	227.2
33	B_1	209.8	E	212.5	E	217.8	E	222.9	B_2	227.3
34	A_2	217.5	E	223.2	E	225.6	E	228.0	E	230.8
35	E	220.2	A_2	224.0	A_1	228.3	A_1	230.4	A_1	232.4
36	A_1	223.8	A_1	226.1	A_2	230.5	B_1	234.7	B_1	237.1
37	E	224.3	E	228.4	B_1	232.3	E	236.5	E	240.4
38	B_1	227.0	B_1	229.7	E	232.4	A_2	236.8	A_2	242.8
39	B_1	232.6	B_1	235.6	B_1	238.8	B_1	242.4	B_1	245.5
40	E	241.8	E	245.6	E	249.5	E	253.4	E	257.1
41	A_1	246.7	A_1	251.0	A_1	255.2	A_1	259.1	A_1	263.1
42	B_2	384.0	B_2	384.5	B_2	385.1	B_2	385.7	B_2	386.3
43	A_2	384.1	A_2	384.8	A_2	385.4	A_2	385.9	A_2	386.5
44	E	386.8	E	387.3	E	387.7	E	388.1	E	388.6
45	A_1	395.8	A_1	396.6	A_1	397.5	A_1	398.4	A_1	399.4
46	B_1	397.2	B_1	398.0	B_1	398.9	B_1	399.8	B1	400.9
47	E	398.0	E	399.0	E	400.0	E	401.1	E	402.2

48	B_2	440.1	B_2	440.5	B_2	440.9	B_2	441.4	B_2	441.8
49	E	441.0	E	441.3	E	441.6	E	441.9	E	442.2
50	A_2	442.0	A_2	442.3	A_2	442.7	A_2	443.1	A_2	443.5
51	A_2	457.4	A_2	457.3	A_2	457.3	A_2	457.5	A_2	457.9
52	E	457.9	E	457.9	E	458.2	E	458.6	E	459.1
53	B_2	459.1	B_2	459.3	B_2	459.7	B_2	460.2	B_2	460.9
54	A_1	516.1	A_1	515.8	A_1	515.5	A_1	515.3	A_1	515.0
55	E	516.4	E	516.2	E	516.0	E	515.9	E	515.7
56	B_1	517.3	B_1	517.2	B_1	517.1	B_1	517.0	B_1	516.9
57	B_2	672.5	B_2	672.9	B_2	673.4	B_2	673.9	B_2	674.5
58	E	673.9	E	674.3	E	674.7	E	675.3	E	675.8
59	A_2	674.8	A_2	675.2	A_2	675.7	A_2	676.2	A_2	676.8
60	A_1	687.0	A_1	688.8	A_1	690.6	A_1	692.3	A_1	694.0
61	B_1	690.4	B_1	692.4	B_1	694.4	B_1	696.3	B_1	698.2
62	E	690.9	E	693.0	E	695.0	E	696.9	E	698.9
63	B_2	811.8	B_2	812.5	B_2	813.2	B_2	813.8	B_2	814.4
64	E	815.3	E	815.9	E	816.5	E	817.1	E	817.6
65	A_2	819.6	A_2	820.2	A_2	820.6	A_2	821.1	A_2	821.5
66	B_2	859.8	B_2	858.4	B_2	857.2	B_2	856.1	B_2	855.1
67	E	860.7	E	859.2	E	857.9	E	856.8	E	855.8
68	A_2	861.4	A_2	859.9	A_2	858.6	A_2	857.5	A_2	856.5
69	A_2	885.4	A_2	885.5	A_2	885.8	A_2	886.3	A_2	886.7
70	B_2	887.4	B_2	887.3	B_2	887.5	B_2	887.9	B_2	888.3
71	E	887.9	E	888.0	E	888.3	E	888.8	E	889.2
72	E	912.3	E	913.0	E	913.7	E	914.5	E	915.2
73	A_1	914.2	A_1	914.8	A_1	915.6	A_1	916.4	B_1	917.1
74	B_1	914.3	B_1	914.9	B_1	915.6	B_1	916.4	A_1	917.1
75	B_1	1015.0	B_1	1016.1	B_1	1017.2	B_1	1018.1	B_1	1019.2
76	E	1016.8	E	1018.0	E	1019.1	E	1020.2	E	1021.3
77	A_1	1021.4	A_1	1022.6	A_1	1023.8	A_1	1025.0	A_1	1026.2
78	A_2	1099.1	A_2	1099.8	A_2	1100.5	A_2	1101.0	A_2	1101.6
79	E	1102.9	E	1103.7	E	1104.5	E	1105.3	E	1106.1
80	B_2	1103.9	B_2	1104.8	B_2	1105.7	B_2	1106.6	B_2	1107.5
81	E	1112.6	E	1114.4	E	1116.1	E	1117.9	E	1119.5
82	B_1	1115.1	B_1	1116.8	B_1	1118.4	B_1	1120.2	B_1	1121.9
83	A_1	1120.9	A_1	1122.7	A_1	1124.5	A_1	1126.3	A_1	1128.0
84	A_1	1166.1	A_1	1166.7	A1	1167.4	A_1	1168.2	A_1	1169.0
85	E	1167.4	E	1168.1	E	1168.8	E	1169.7	E	1170.5
86	B_1	1171.1	B_1	1172.0	B_1	1172.8	B_1	1173.8	B_1	1174.7
87	B_2	1274.4	B_2	1274.9	B_2	1275.4	B_2	1275.8	B_2	1276.2
88	A_2	1279.0	A_2	1279.5	A_2	1280.1	A_2	1280.7	A_2	1281.2
89	E	1279.3	E	1280.0	E	1280.6	E	1281.3	E	1281.9
90	A_2	1317.4	A_2	1320.5	A_2	1323.6	A_2	1326.7	A_2	1329.7
91	B_2	1317.6	B_2	1320.7	B_2	1323.8	B_2	1326.9	B_2	1330.0
92	E	1318.1	E	1321.2	E	1324.2	E	1327.3	E	1330.3
93	B_2	1408.6	B_2	1410.0	B_2	1411.3	B_2	1412.7	B_2	1414.1
94	E	1410.2	E	1411.4	E	1412.4	E	1413.5	E	1414.5
95	B_1	1411.8	E	1411.4	B_1	1413.8	B_1	1414.7	B_1	1415.7
96	E	1412.4	B_1	1412.8	E	1414.9	E	1416.3	E	1417.7
97	A_1	1413.6	E	1413.6	A_1	1415.7	A_1	1416.8	A_1	1417.8
98	A_2	1415.4	A_1	1414.6	A_2	1418.5	A_2	1420.0	A2	1421.6
99	B_2	1547.8	A_2	1416.9	B_2	1551.4	B_2	1553.2	B_2	1555.0
100	E	1549.9	B_2	1549.6	E	1553.6	E	1555.4	E	1557.2
101	A_2	1551.3	E	1551.8	A_2	1554.9	A_2	1556.6	A_2	1558.4
102	B_1	1568.4	A_2	1553.1	B_1	1573.3	B_1	1575.7	B_1	1578.0
103	E	1569.1	B_1	1570.9	E	1574.0	E	1576.4	E	1578.7
104	A_1	1571.2	E	1571.6	A_1	1576.2	A_1	1578.6	A_1	1581.0
105	A_2	3149.6	A_1	1573.7	A_2	3154.2	E	3156.5	E	3158.9
106	E	3149.7	A_2	3151.9	E	3154.2	A_2	3156.6	A_2	3158.9
107	B_2	3149.7	E	3151.9	B_2	3154.2	B_2	3156.6	B_2	3158.9

108	A_1	3152.6	B_2	3151.9	A_1	3157.3	A_1	3159.6	A_1	3162.0
109	E	3152.7	A_1	3155.0	E	3157.4	E	3159.7	E	3162.1
110	B_1	3153.1	E	3155.0	B_1	3157.8	B_1	3160.2	B_1	3162.7
111	E	3166.0	B_1	3155.5	E	3178.5	E	3184.6	E	3190.5
112	A_1	3166.1	E	3172.3	A_1	3178.6	A_1	3184.7	A_1	3190.6
113	B_1	3168.1	A_1	3172.5	B_1	3180.9	B_1	3187.0	B_1	3193.0

Supplementary Table 4.3: PBE-TS simulated vibrational frequencies for (**1**) from 5 to 7 GPa. The irreducible representation (IR) is shown in each case.

#	IR (5.0 GPa)	$\boldsymbol{\nu}$(5.0)	IR (5.5 GPa)	$\boldsymbol{\nu}$(5.5)	IR (6.0 GPa)	$\boldsymbol{\nu}$(6.0)	IR (6.5 GPa)	$\boldsymbol{\nu}$(6.5)	IR (7.0 GPa)	$\boldsymbol{\nu}$(7.0)
4	A_2	-10.7	A_2	-13.5	A_2	-15.8	A_2	-17.6	A_2	-19.5
5	B_2	31.9	B_2	33.2	B_2	33.7	B_2	34.3	B_2	34.9
6	B_1	62.2	B_1	63.1	B_1	64.0	B1	64.9	B_1	65.5
7	A_2	62.8	A_2	65.1	A_2	67.1	E	68.8	E	70.2
8	E	63.3	E	65.3	E	67.1	A_2	69.0	E	70.2
9	E	68.3	E	69.7	E	71.1	E	72.5	A_2	71.2
10	B_2	78.7	B_2	80.4	B_2	82.0	B_2	83.3	E	73.8
11	E	83.1	E	84.3	E	85.4	E	86.4	E	73.8
12	A_2	89.5	A_2	91.7	A_2	93.6	A_2	95.4	B_2	84.5
13	A_1	94.2	A_1	96.5	A_1	98.5	A_1	100.5	E	87.2
14	E	98.0	E	100.1	E	102.2	E	104.1	E	87.2
15	A_1	107.9	A_1	109.8	A_1	111.6	A_1	113.4	A_2	96.9
16	A_2	115.8	A_2	117.0	A_2	118.1	A_2	119.1	A_1	102.3
17	B_2	118.4	B_2	119.6	B_2	120.9	B_2	122.2	E	106.2
18	E	128.5	E	129.5	E	130.6	E	131.6	E	106.2
19	A_2	134.4	B_2	136.4	B_2	138.0	B_2	139.6	A_1	115.0
20	B_2	134.6	A_2	136.9	A2	139.3	A_2	141.8	A_2	120.1
21	B_1	140.2	B_1	142.6	B_1	145.3	B_1	148.0	B_2	123.6
22	E	145.4	E	147.0	E	148.3	E	149.5	E	132.6
23	E	154.8	E	156.6	E	158.4	E	160.1	E	132.6
24	B_2	163.6	B_2	165.1	B_2	166.5	B_2	167.8	B_2	141.0
25	A_2	167.1	A_2	168.6	A_2	170.1	A_2	171.5	A_2	144.3
26	E	178.0	E	179.8	E	181.3	E	182.8	B_1	149.6
27	E	183.7	E	185.5	E	187.5	E	189.5	E	150.5
28	A_2	184.6	A_2	186.7	A_2	188.7	A_2	190.7	E	150.5
29	B_2	195.5	B_2	197.9	B_2	200.1	B_2	202.3	E	161.7
30	B_1	219.4	B_1	221.1	B_1	222.5	B_1	223.8	E	161.7
31	A_1	219.5	A_1	221.3	A_1	223.0	A_1	224.7	B_2	169.0
32	E	230.3	E	232.6	E	234.5	E	236.3	A_2	173.2
33	B_2	232.3	A_1	236.3	A_1	238.0	A_1	239.7	E	184.0
34	A_1	234.4	B_2	237.1	B_2	241.6	B_1	245.8	E	184.0
35	E	234.5	E	238.5	E	242.5	B_2	245.8	E	191.6
36	B_1	239.4	B_1	241.6	B_1	243.7	E	246.3	E	191.6
37	E	244.4	E	248.1	E	251.7	E	255.3	A_2	192.6
38	A_2	248.5	B_1	251.7	B_1	254.7	B_1	257.7	B_2	204.3
39	B_1	248.7	A_2	254.0	A_2	259.1	A_2	263.9	B_1	224.9
40	E	260.7	E	264.3	E	267.8	E	271.3	A_1	226.3
41	A_1	266.9	A_1	270.6	A_1	274.2	A_1	277.6	E	237.9
42	B_2	386.8	B_2	387.4	B_2	387.9	B_2	388.4	E	237.9
43	A_2	387.1	A_2	387.7	A_2	388.3	A_2	388.9	A_1	241.3
44	E	389.1	E	389.5	E	390.0	E	390.4	B_1	247.6
45	A_1	400.5	A_1	401.5	A_1	402.6	A_1	403.8	B_2	249.7
46	B_1	401.8	B_1	402.7	B_1	403.6	B_1	404.6	E	249.9
47	E	403.4	E	404.6	E	405.8	E	407.0	E	249.9
48	B_2	442.2	B_2	442.6	B_2	443.0	B_2	443.5	E	258.8
49	E	442.6	E	442.9	E	443.2	E	443.5	E	258.8

50	A_2	443.9	A_2	444.3	A_2	444.7	A_2	445.2	B_1	260.4
51	A_2	458.3	A_2	458.9	A_2	459.5	A_2	460.3	A_2	268.4
52	E	459.8	E	460.5	E	461.4	E	462.4	E	275.1
53	B_2	461.7	B_2	462.7	B_2	463.7	B_2	464.8	E	275.1
54	A_1	514.8	A_1	514.6	A_1	514.3	A_1	514.1	A_1	280.8
55	E	515.6	E	515.4	E	515.2	E	515.1	B_2	388.9
56	B_1	516.8	B_1	516.7	B_1	516.6	B_1	516.5	A_2	389.5
57	B_2	675.1	B_2	675.8	B_2	676.4	B_2	677.0	E	390.8
58	E	676.4	E	677.1	E	677.7	E	678.4	E	390.8
59	A_2	677.5	A_2	678.1	A_2	678.8	A_2	679.5	A_1	404.7
60	A_1	695.6	A_1	697.2	A_1	698.8	A_1	700.3	B_1	405.7
61	B_1	700.0	B_1	701.8	B_1	703.6	B_1	705.3	E	408.4
62	E	700.7	E	702.6	E	704.4	E	706.2	E	408.4
63	B_2	815.1	B_2	815.7	B_2	816.4	B_2	817.1	E	443.9
64	E	818.2	E	818.8	E	819.4	E	820.0	E	443.9
65	A_2	822.0	A_2	822.5	A_2	823.0	A_2	823.5	B_2	443.9
66	B_2	854.3	B_2	853.6	B_2	853.0	B_2	852.6	A_2	445.6
67	E	855.0	E	854.3	E	853.7	E	853.3	A_2	461.1
68	A_2	855.7	A_2	855.0	A_2	854.5	A_2	854.1	E	463.5
69	A_2	887.2	A_2	887.7	A_2	888.1	A_2	888.4	E	463.5
70	B_2	888.7	B_2	889.1	B_2	889.5	B_2	889.9	B_2	466.0
71	E	889.7	E	890.2	E	890.6	E	891.0	A_1	513.8
72	E	915.9	E	916.5	E	917.0	E	917.5	E	514.9
73	B_1	917.8	B_1	918.4	B_1	918.9	B_1	919.3	E	514.9
74	A_1	917.8	A_1	918.4	A_1	919.0	A_1	919.4	B_1	516.4
75	B_1	1020.1	B_1	1021.1	B_1	1022.0	B_1	1023.0	B_2	677.7
76	E	1022.4	E	1023.4	E	1024.5	E	1025.5	E	679.0
77	A_1	1027.3	A_1	1028.5	A_1	1029.5	A_1	1030.6	E	679.0
78	A_2	1102.2	A_2	1102.8	A_2	1103.4	A_2	1103.9	A_2	680.1
79	E	1106.9	E	1107.8	E	1108.5	E	1109.3	A_1	701.9
80	B_2	1108.5	B_2	1109.5	B_2	1110.4	B_2	1111.4	B_1	707.0
81	E	1121.2	E	1122.8	E	1124.5	E	1126.1	E	707.9
82	B_1	1123.6	B_1	1125.2	B_1	1126.9	B_1	1128.5	E	707.9
83	A_1	1129.8	A_1	1131.5	A_1	1133.2	A_1	1135.0	B_2	817.8
84	A_1	1169.8	A_1	1170.7	A_1	1171.6	A_1	1172.4	E	820.7
85	E	1171.4	E	1172.3	E	1173.3	E	1174.2	E	820.7
86	B_1	1175.7	B_1	1176.7	B_1	1177.7	B_1	1178.7	A_2	824.0
87	B_2	1276.6	B_2	1276.9	B_2	1277.3	B_2	1277.5	B_2	852.2
88	A_2	1281.8	A_2	1282.3	A_2	1282.8	A_2	1283.3	E	852.9
89	E	1282.5	E	1283.1	E	1283.6	E	1284.1	E	852.9
90	A_2	1332.7	A_2	1335.8	A_2	1338.7	A_2	1341.7	A_2	853.8
91	B_2	1333.1	B_2	1336.1	B_2	1339.1	B_2	1342.1	A_2	888.7
92	E	1333.3	E	1336.3	E	1339.3	E	1342.2	B_2	890.2
93	B_2	1415.5	E	1416.6	E	1417.6	E	1418.6	E	891.3
94	E	1415.6	B_2	1416.9	B_2	1418.3	B_1	1419.6	E	891.3
95	B_1	1416.7	B_1	1417.7	B_1	1418.6	B_2	1419.8	E	917.8
96	A_1	1418.8	A_1	1419.9	A_1	1420.9	A_1	1421.9	E	917.8
97	E	1419.2	E	1420.6	E	1422.1	E	1423.6	B_1	919.7
98	A_2	1423.2	A_2	1424.8	A_2	1426.4	A2	1428.0	A_1	919.8
99	B_2	1556.8	B_2	1558.6	B_2	1560.5	B_2	1562.3	B_1	1023.9
100	E	1559.1	E	1560.9	E	1562.7	E	1564.6	E	1026.4
101	A_2	1560.2	A2	1562.0	A_2	1563.8	A2	1565.6	E	1026.4
102	B_1	1580.3	B_1	1582.6	B_1	1584.8	B1	1587.0	A_1	1031.6
103	E	1581.0	E	1583.3	E	1585.5	E	1587.7	A_2	1104.6
104	A_1	1583.4	A_1	1585.7	A_1	1588.0	A_1	1590.2	E	1110.1
105	E	3161.3	E	3163.7	E	3166.0	E	3168.4	E	1110.1
106	A_2	3161.3	A2	3163.7	A_2	3166.1	B_2	3168.5	B_2	1112.2
107	B_2	3161.3	B_2	3163.7	B_2	3166.1	A_2	3168.5	E	1127.8
108	A_1	3164.4	A_1	3166.8	A_1	3169.2	A_1	3171.6	E	1127.8
109	E	3164.6	E	3167.0	E	3169.4	E	3171.9	B_1	1130.0

110	B_1	3165.1	B_1	3167.5	B_1	3169.9	B_1	3172.4	A_1	1136.7
111	E	3196.2	E	3201.8	E	3207.4	E	3212.8	A_1	1173.1
112	A_1	3196.3	A_1	3201.9	A_1	3207.5	A_1	3212.9	E	1175.2
113	B_1	3198.8	B_1	3204.6	B_1	3210.2	B_1	3215.7	E	1175.2

Our PBE-TS simulated frequencies, obtained in this work within the framework of Density Functional Perturbation Theory (DFPT), agree well with those previously reported *via* the Finite Differences (FD) method with PBE-D3, Table S4.4.

Supplementary Table 4.4: Comparison of the PBE-TS simulated frequencies obtained in this work against the PBE-D3 frequencies reported previously.[18]

#	Literature PBE-D3 Finite Differences[18]	This work: PBE-TS DFPT	#	Literature PBE-D3 Finite Differences[18]	This work: PBE-TS DFPT
4	26.944	25.2	59	675.929	676.2
5	28.839	27.8	60	681.692	676.9
6	38.754	37.3	61	684.204	679.3
7	39.527	39.9	62	684.925	679.7
8	39.619	45.9	63	808.992	807.6
9	44.095	46.7	64	812.676	811.3
10	51.407	50.8	65	817.547	816.2
11	56.600	57.6	66	867.488	868.1
12	58.699	57.7	67	868.357	869.9
13	58.918	64.6	68	868.436	870.8
14	65.428	65.8	69	891.164	891.2
15	85.021	80.0	70	892.673	893.7
16	98.522	93.3	71	892.937	894.5
17	99.098	96.1	72	907.960	913.0
18	101.221	96.3	73	909.512	914.6
19	101.605	98.4	74	909.714	914.8
20	105.280	98.9	75	1015.317	1009.2
21	106.892	100.1	76	1016.719	1010.5
22	119.810	117.7	77	1021.089	1014.7
23	138.041	134.6	78	110.891	1094.0
24	141.312	143.6	79	1103.559	1097.3
25	143.063	144.6	80	1104.086	1098.1
26	151.544	144.9	81	1109.229	1103.0
27	162.612	157.4	82	1111.667	1105.8
28	168.846	161.4	83	1116.670	1110.7
29	169.842	165.6	84	1165.617	1163.1
30	187.367	184.7	85	1166.237	1163.8
31	187.844	186.0	86	1169.508	1166.7
32	195.033	188.2	87	1273.517	1269.9
33	199.651	195.1	88	1276.589	1273.2
34	201.173	196.3	89	1276.663	1273.5
35	203.159	197.3	90	1307.749	1301.6
36	210.895	211.8	91	1308.002	1302.3
37	210.936	214.2	92	1308.927	1303.1
38	212.971	214.3	93	1405.579	1401.9
39	222.794	219.0	94	1407.828	1403.8
40	230.559	223.9	95	1410.211	1406.4
41	231.656	225.3	96	1410.438	1406.7
42	386.900	380.7	97	1411.706	1407.4
43	387.048	380.9	98	1411.728	1407.9
44	390.022	384.1	99	1543.686	1537.8
45	393.700	390.5	100	1545.804	1539.8
46	395.505	392.3	101	1547.470	1541.4
47	395.706	392.4	102	1559.688	1554.9
48	440.346	437.8	103	1560.350	1555.5

49	441.600	439.2	104	1562.076	1557.2
50	442.539	440.1	105	3139.117	3132.1
51	461.775	459.9	106	3139.147	3132.1
52	461.794	460.0	107	3140.487	3133.1
53	462.496	460.7	108	3141.432	3136.0
54	515.373	518.4	109	3141.523	3136.0
55	515.489	518.6	110	3141.542	3136.3
56	515.889	518.9	111	3144.659	3138.8
57	673.673	673.6	112	3144.915	3139.3
58	675.310	675.2	113	3145.420	3139.9

Supplementary Note 4.2| Normal Mode Eigenvector Analysis

Vibrational analysis (Supplementary Note 4.1) demonstrates softening of a low frequency A_2 species with increasing pressure. To analyse the normal mode in more detail we extracted the average absolute cartesian components of the corresponding eigenvector, Supplementary Table 4.5 and Supplementary Figure 4.1. Owing to the orthogonal crystallographic axes, the cartesian axes align as *x*=*a*, *y*=*b*, and *z*=*c*. It is hence immediately apparent that the A_2 mode is polarized almost exclusively along the crystallographic *c* axis (i.e. the along the CP chains). The increase in magnitude along the *c* axis is consistent with softening of the vibrational mode (see Supplementary Tables 4.1-4.3 and Figure 4 in the main manuscript)

Supplementary Table 4.5: Cartesian components of the A_2 'soft mode' eigenvector (see Supplementary Tables 4.1-4.3)

Pressure /GPa	X	Y	Z
0	0.71	0.71	4.62
0.5	0.72	0.72	4.76
1.0	0.73	0.73	4.83
1.5	0.74	0.74	4.90
2.0	0.74	0.74	4.95
2.5	0.75	0.75	4.99
3.0	0.76	0.76	5.03
3.5	0.76	0.76	5.06
4.0	0.77	0.77	5.08
4.5	0.77	0.77	5.09
5.0	0.78	0.78	5.11
5.5	0.79	0.79	5.12
6.0	0.80	0.80	5.13
6.5	0.81	0.81	5.14
7.0	0.82	0.82	5.15

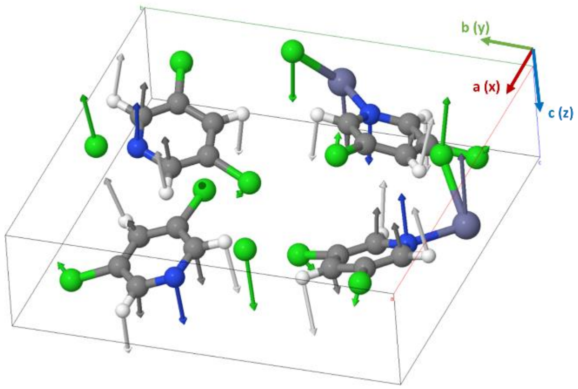

Supplementary Figure 4.1: Visual representation of the *A2* ‘soft mode’ eigenvector simulated by PBE-TS at P=0 GPa. Atoms are colored as H – white; C – grey; N – blue; Cl – green; Zn – dark grey.

Supplementary Note 5| Raman Spectroscopy

Raman spectra were collected for a powdered sample of (1) loaded in a diamond anvil cell in a 1:1 pentane-isopentane solution. Upon closing the cell, a pressure of < 0.2 GPa was measured. As this is within the accuracy of the measurement at these pressures, this is taken as an upper limit. The Raman band positions are in good agreement with our previously reported[18] ambient pressure Raman spectra (see Supplementary Table 5.1) albeit with slightly blue shifted bands in most cases, indicating some pressure is likely present. DFT-TS computed bands (p = 0 GPa) are compared against experimentally obtained values, Supplementary Table 5.1, and demonstrate excellent agreement. Note that as (**1**) crystallizes in space group $P\bar{4}b2$, point group $D_{2\mathrm{h}}$ $(-42m)$, only those normal modes with point symmetries A_1, B_1, B_2, and E are Raman active. The experimental Raman spectra at elevated pressures are shown in Supplementary Figures 5.2-5.3

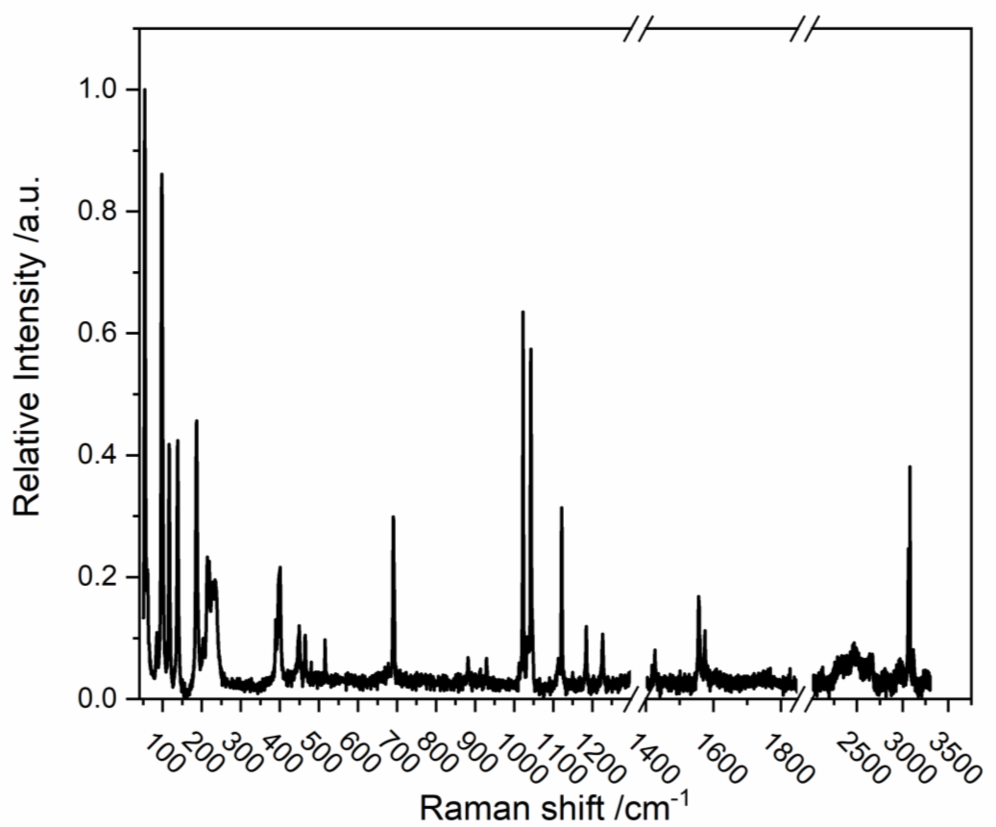

Supplementary Figure 5.1: Raman spectrum for (**1**) loaded at *ca.* 0.2 GPa pressure. Band assignments are given for select features according to PBE-TS simulated eigenvectors (see Supplementary Table 5.1).

Supplementary Table 5.1: Experimental Raman band frequencies for (**1**), alongside DFT (PBE-TS) simulated values.

N	Bhattacharya *et al.*[18]	*ca*. 0.2 GPa DAC	DFT
1	--	53.52	50.8
2	--	60.87	57.7/64.6
3	--	84.47	80.0
4	--	97.54	96.4/98.5/100.2
5	116.17	116.32	117.7
6	137.78	138.17	134.6/143.6/144.9
7	189.08	186.80	184.8/186.1
8	--	202.76	197.4
9	--	209.96	211.8
10	213.67	214.58	214.3
11	218.23	218.69	219.1
12	--	226.89	223.9
13	235.65	233.55	225.3
14	398.89	389.34	384.2/390.6/392.3/392.5
15	447.30	448.55	439.2
16	464.46	464.87	460.0
17	515.09	515.54	518.5
18	678.20	--	
19	690.77	690.69	679.7
20	882.30	881.56	869.9/893.8
21	913.90	--	
22	929.20	929.06	914.6/914.8
23	1012.70	--	1009.3/
24	1022.67	1023.06	1010.6/1014.6/1014.7
25	1034.10	1033.22	
26	1042.64	1042.90	
27	1111.80	--	1094.3/1097.4/1098.1
28	1121.66	1122.08	1110.8
29	1150.00	--	
30	1181.20	1184.50	1163.8/1166.8/1166.7
31	1226.40	1227.10	1267.9
32	--	1427.09	1407.9
33	1558.89	1556.74	1555.6
34	1575.10	1575.52	1557.2
35	3060.60	3058.34	3132.1
36	3062.70	3062.73	3136.3
37	3079.67	3079.25	3138.8
38	3117.60	--	3139.4

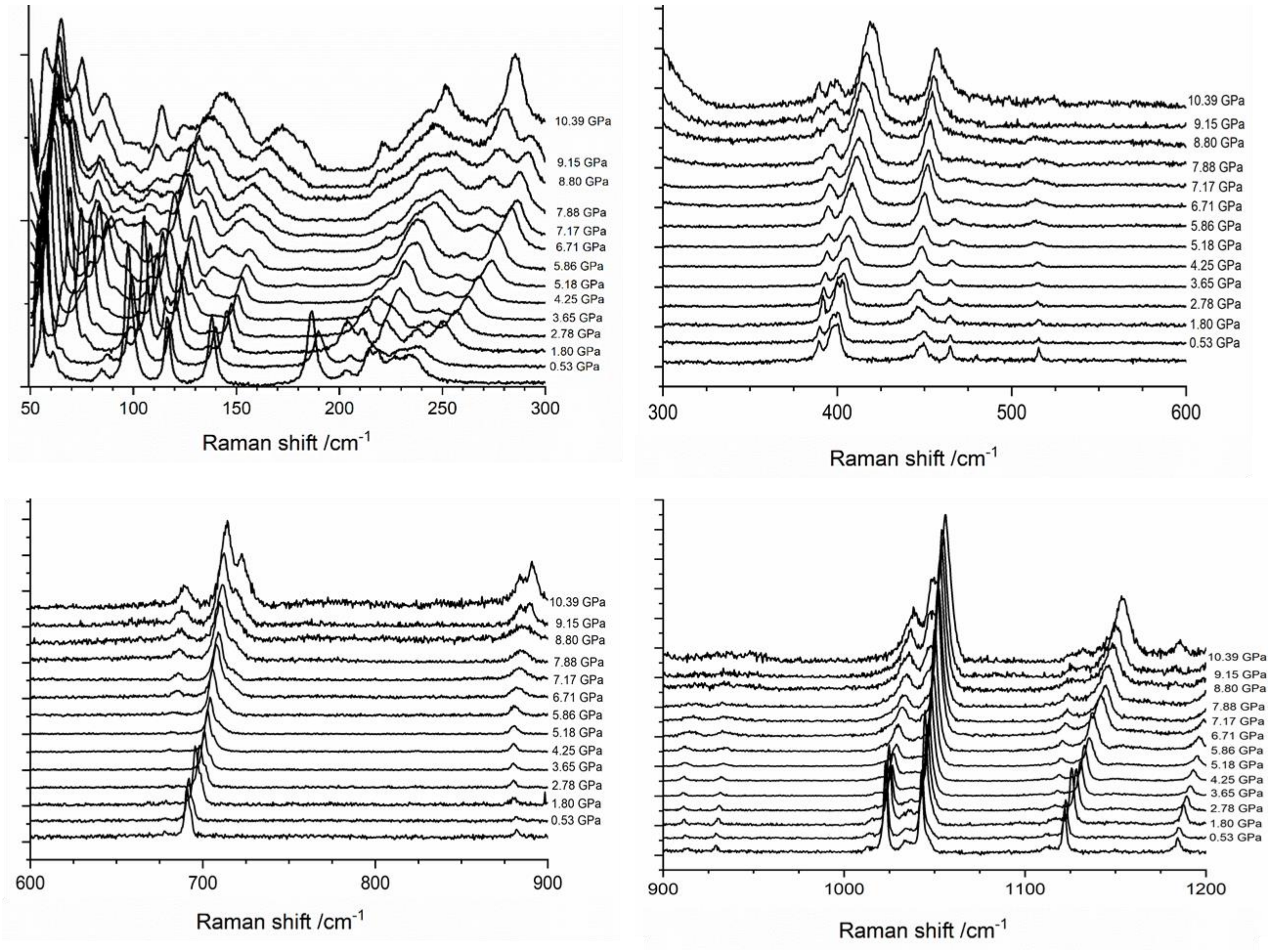

Supplementary Figure 5.2: Experimental Raman spectra of (**1**) at elevated pressures for spectral range 50-1200 cm^{-1}.

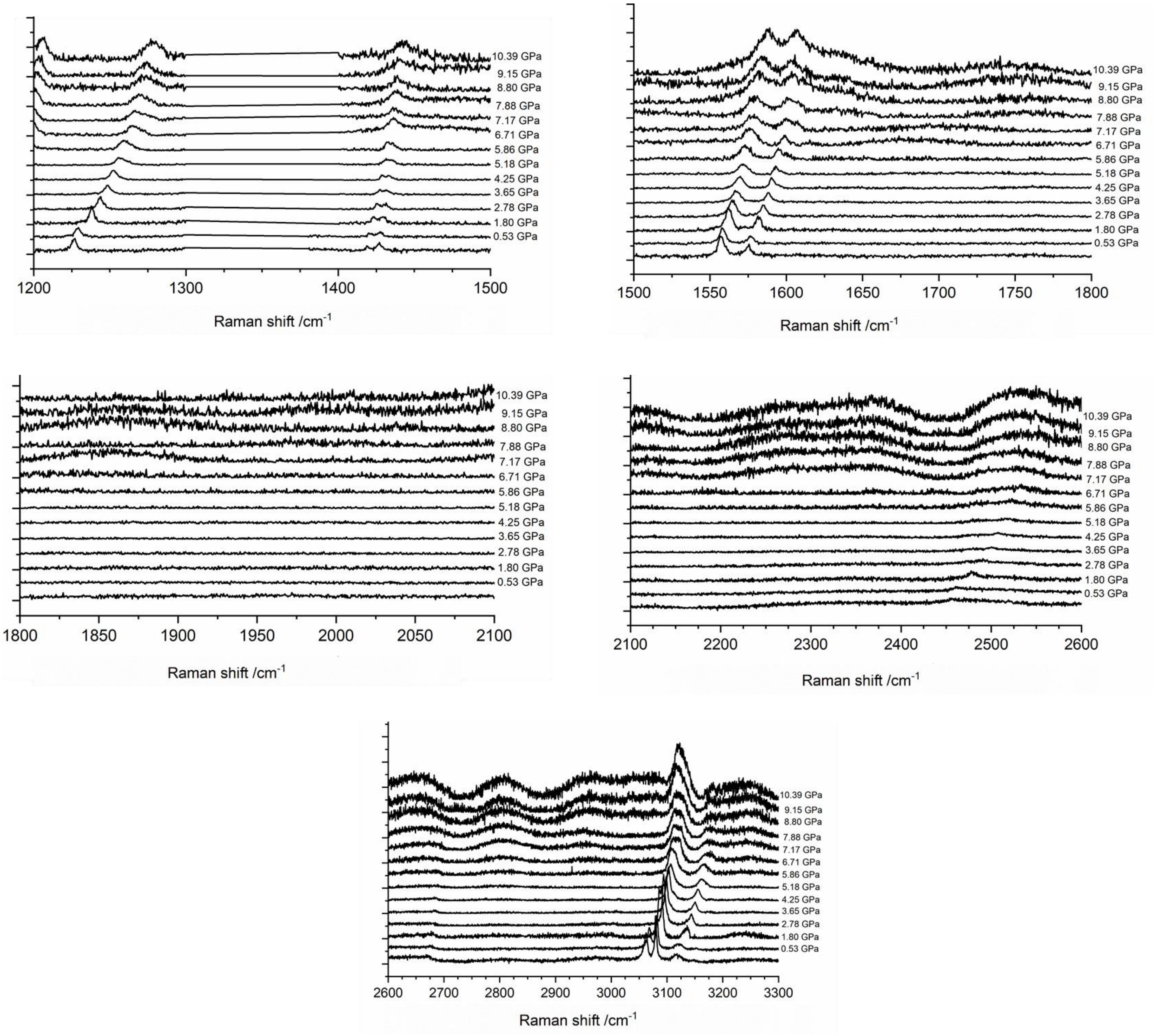

Supplementary Figure 5.3: Experimental Raman spectra of (**1**) at elevated pressures for spectral range 1200-3300 cm^{-1}.

Supplementary Note 6| Anisotropic Unit Cell Response

Supplementary Note 6.1| Simulated Anisotropic Compression

The unit cell of (**1**) was relaxed under an anisotropic external load, applied equally along the crystallographic *a* and *b* axes. The *c* axis was left to relax in the absence of any constraint. The *a* and *b* axes compressed under the load, whereas the *c* axis was found to expand, Supplementary Figure 6.1 and Supplementary Table 6.1.

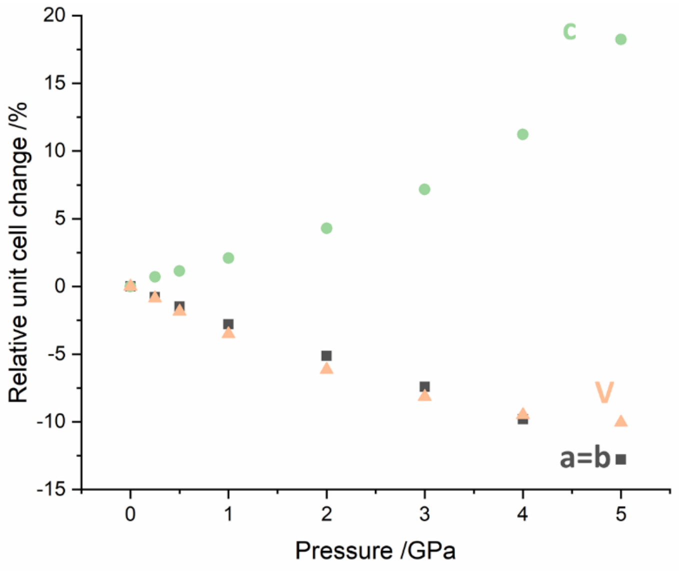

Supplementary Figure 6.1: Effects of simulated (PBE-TS) anisotropic compression of the unit cell upon compression of the crystallographic *a* and *b* axes. The crystallographic *c* axis was left to relax under no external field.

Supplementary Table 6.1: Unit cell parameters obtained from relaxation (PBE-TS) of (**1**) under anisotropic external field along the *a* and *b* vectors. The *c* axis was left to relax under no external load.

p/GPa	*a* /Å	*b* /Å	*c* /Å	V /Å^3
0	13.872 (+0.4%)	13.872 (+0.4%)	3.681 (+1.12%)	710.421 (+1.86%)
0.25	13.763	13.763	3.707	702.160
0.5	13.666	13.666	3.723	695.373
1.0	13.485	13.485	3.758	683.466
2.0	13.161	13.161	3.839	664.901
3.0	12.843	12.843	3.945	650.745
4.0	12.513	12.513	4.094	641.100
5.0	12.100	12.100	4.352	637.217

With compression along the *a* and *b* axes, the lowest frequency A_2 phonon mode was again found to soften with pressure, Supplementary Figure 6.2. Moreover, the eigenvectors of this normal mode correspond to the same soft mode observed under hydrostatic compression, Supplementary Figure 6.3. This confirms that softening of this vibrational normal mode results

from compression along the *a* and *b* axes. This can be interpreted in that the potential energy surface along the *c* axis remains largely unperturbed, whereas the PES along the *a* and *b* vectors hardens, thereby converting the ambient pressure 'minimum energy structure' into a 'maximum energy structure' along the PES in the *c*-direction. This leads to phonon softening and drives the observed phase transition.

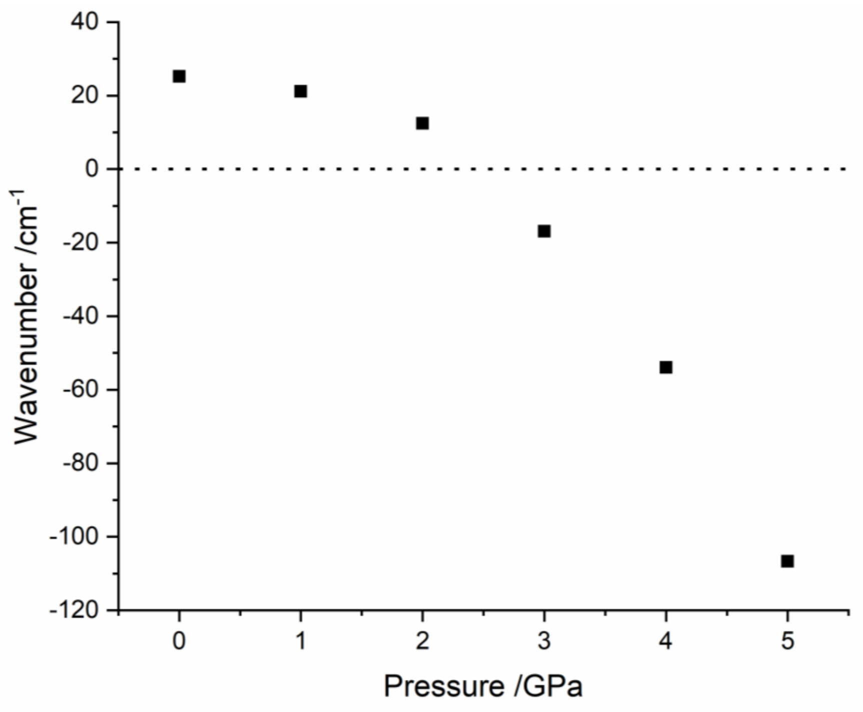

Supplementary Figure 6.2: Softening of the lowest frequency A_2 phonon mode with compression of the crystallographic *a* and *b* axes.

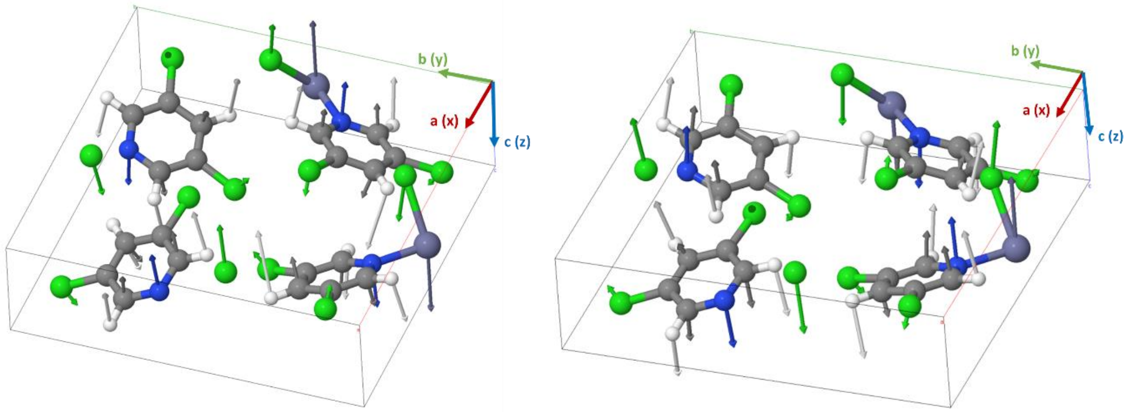

Supplementary Figure 6.3: Eigenvectors of the soft mode leading to the phase transition in (**1**). (Left) Soft mode eigenvectors obtained from anisotropic compression of the *a* and *b* axes. (Right) Soft mode eigenvectors obtained from hydrostatic compression of the unit cell axes. Note the eigenvector phases have been inverted but are equivalent by symmetry.

Supplementary Note 7| Microfocus Synchrotron X-Ray Diffraction

Supplementary Note 7.1| Reciprocal Space of Bent Crystals

To better understand the effects of bending on the crystallographic structure of (**1**), X-ray diffraction data were collected for select locations within the deformed region of the single crystals using microfocus (0.922 x 3.67 μm) synchrotron X-ray diffraction, Supplementary Figure 7.1. Data collected directly within the bent region of the crystal (Supplementary Figure 7.1b-e) exhibit a significant degree of mosaicity, which appears to be dominant in the crystallographic *ac* and *bc* planes. Notably this suggests formation of microdomains between CP chains. Data collected 100 μm outside of the bend (Supplementary Figure 7.1f-i) show very clear indications of twinning (see Supplementary Figure 7.1i). These data suggest that deformation of the lattice is localized primarily to within the bent region itself, relaxing towards the parent lattice structure with distance from the bend.

We note that slight indications for the onset of splitting in Bragg reflections is visible in the 2D diffraction images for data collected at 9.35 GPa (see Supplementary Figure 7.1j). The origin of this splitting is not yet known but is presumably due to a loss of hydrostaticity of the pressure-transmitting medium at these elevated pressures. Loss of hydrostaticity leads to shear stress being exerted on the crystal, somewhat akin to three-point-bending. Notably, these shear distortions are fully reversible upon decompression, unlike the distortions observed by three-point-bending.

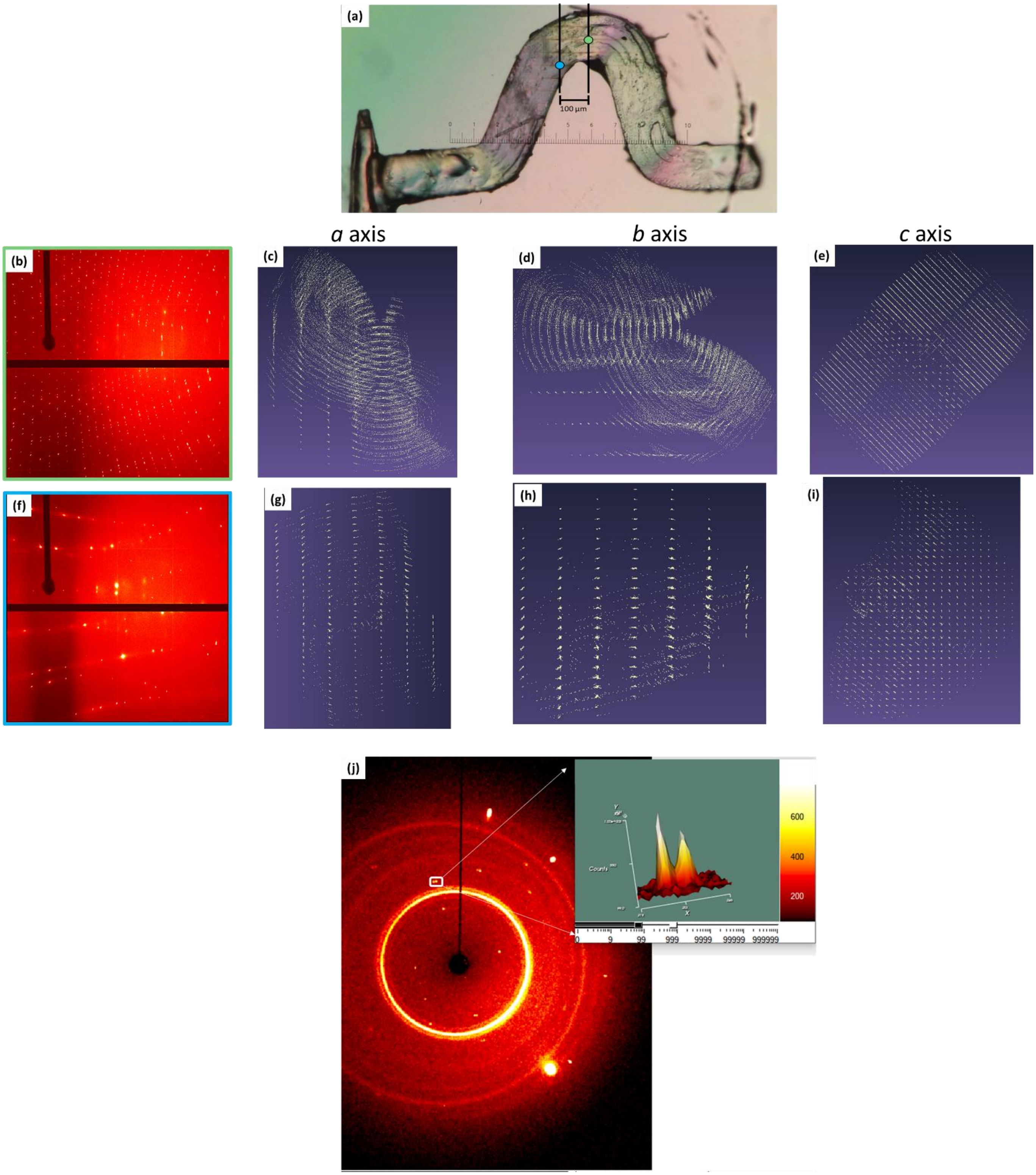

Supplementary Figure 7.1: Microfocus synchrotron X-ray diffraction data collected for select regions within the bend single crystal of (**1**), at positions shown in (a). (b-i) Diffraction profiles and Ewald representations of reciprocal space for data collected in the green and blue regions of the crystal (see (a)), along crystallographic *a*-, *b-, and* c-axes*.* (j) 2D diffraction profile for diffraction

data collected at 9.35 GPa with inset showing the 3D representation of splitting in a representative Bragg reflection.

Supplementary Note 7.2| Diffraction across bent region

Microfocus synchrotron X-ray diffraction was collected for a series of points (spaced 50 μm apart) to explore how different regions within the bend are affected by the mechanical perturbation, Supplementary Figure 7.2. Notably, the diffraction profiles obtained from each of these regions, Supplementary Figure 7.3, suggest no marked change across the region, with the exception of the outermost point (A in Supplementary Figure 7.3). The diffraction profile for this outermost point exhibits extensive smearing of the Bragg reflections, suggesting significant strain remains within the lattice at this point. We note that this may also result from the peeling of surface layers from the crystal. In contrast, all other points exhibit well defined Bragg reflections, albeit with a significant degree of mosaicity (see comparison with Figure 5 in the main manuscript).

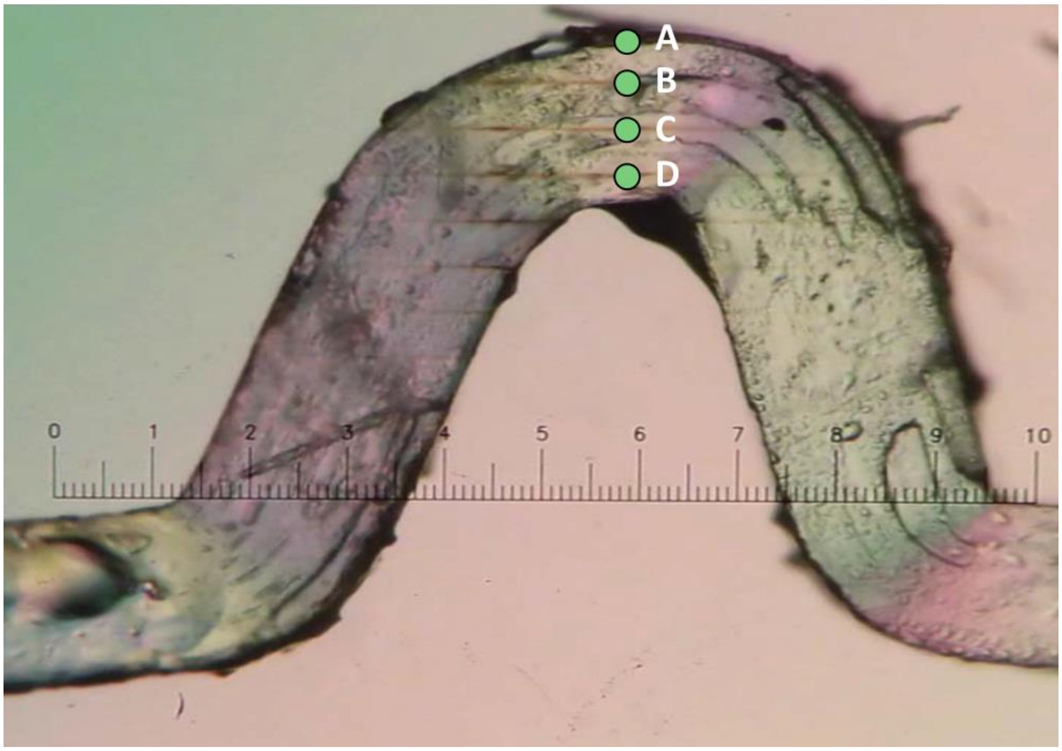

Supplementary Figure 7.2: Location at which microfocus synchrotron X-ray diffraction data were collected over the bent region of a single crystal of (**1**). Note that each increment on the scale bar is equivalent to 8.33 μm. Unit cell parameters extracted from each region are tabulated in Supplementary Table 7.1 and 2D diffraction profiles are shown in Supplementary Figure 7.3

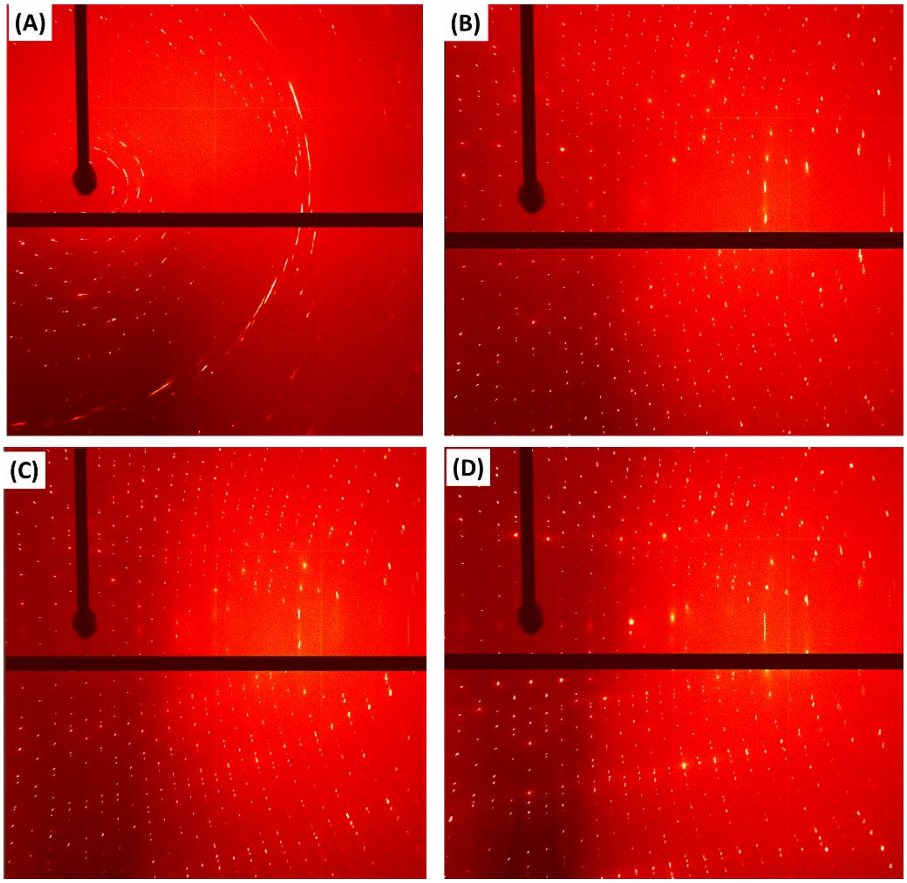

Supplementary Figure 7.3: 2D diffraction images taken over the *hk*0 plane for different points within the bent region of the unit cell. See Supplementary Figure 7.2 for positions A-D.

Supplementary Note 7.3| Unit Cell Parameters of Bent Region

Unit cell dimensions could be successfully extracted from a series of points across the bent region of (**1**), from the convex (A in Supplementary Figure 7.2) to the concave (D in Supplementary Figure 7.2) sides of the crystal. Note that successive points are separated vertically by 50 μm steps. No marked changes in the unit cell geometry were observed across this region, Supplementary Table 7.1, suggesting that no residual stress on the unit cell is not retained following plastic deformation. We do note an exception on the outermost edge of the crystal (position A) which appears to have expanded along all three axes ($\Delta V \approx 1\%$), consistent with tensile stress on the convex side of the arc.

We note that the unit cell geometry – consistent with the more significantly distorted 2D diffraction profile observed in Supplementary Figure 7.3A – is larger than the geometry obtained from the remaining positions within the bent crystal. The increase in volume is consistent with tensile stresses being present on the outer surface of the crystal. It is however possible that the data collected from the outer surface represents a 'peeled' layer of the crystal, requiring further dedicated investigation.

Supplementary Table 7.1: Unit cell parameters obtained from microfocus synchrotron X-ray diffraction data across the bent region of a single crystal of (1), corresponding to Supplementary Figure 7.2. Note that symmetry has been constrained to $P\bar{4}b2$ in all cases.

**Position**	**a /Å**	**b /Å**	**c /Å**	**Vol /Å^3**
A	13.9336(13)	13.9336(13)	3.6705(4)	712.61(15)
B	13.8847(8)	13.8847(8)	3.6618(3)	705.94(10)
C	13.8849(7)	13.8849(7)	3.6614(2)	705.88(8)
D	13.8843(7)	13.8843(7)	3.6613(3)	705.80(9)

Supplementary References

(1) Shen, G.; Wang, Y.; Dewaele, A.; Wu, C.; Fratanduono, D. E.; Eggert, J.; Klotz, S.; Dziubek, K. F.; Loubeyre, P.; Fat'yanov, O. V.; Asimow, P. D.; Mashimo, T.; Wentzcovitch, R. M. M.; other members of the IPPS task group. Toward an International Practical Pressure Scale: A Proposal for an IPPS Ruby Gauge (IPPS-Ruby2020). *High Press. Res.* **2020**, *40* (3), 299–314. https://doi.org/10.1080/08957959.2020.1791107.

(2) Dolomanov, O. V.; Bourhis, L. J.; Gildea, R. J.; Howard, J. A. K.; Puschmann, H. *OLEX2* : A Complete Structure Solution, Refinement and Analysis Program. *J. Appl. Crystallogr.* **2009**, *42* (2), 339–341. https://doi.org/10.1107/S0021889808042726.

(3) Sheldrick, G. M. *SHELXT* – Integrated Space-Group and Crystal-Structure Determination. *Acta Crystallogr. Sect. Found. Adv.* **2015**, *71* (1), 3–8. https://doi.org/10.1107/S2053273314026370.

(4) Sheldrick, G. M. Crystal Structure Refinement with *SHELXL*. *Acta Crystallogr. Sect. C Struct. Chem.* **2015**, *71* (1), 3–8. https://doi.org/10.1107/S2053229614024218.

(5) Schmid, T.; Dariz, P. Raman Microspectroscopic Imaging of Binder Remnants in Historical Mortars Reveals Processing Conditions. *Heritage* **2019**, *2* (2), 1662–1683. https://doi.org/10.3390/heritage2020102.

(6) Yasuda, N.; Fukuyama, Y.; Toriumi, K.; Kimura, S.; Takata, M.; Garrett, R.; Gentle, I.; Nugent, K.; Wilkins, S. Submicrometer Single Crystal Diffractometry for Highly Accurate Structure Determination; Melbourne (Australia), 2010; pp 147–150. https://doi.org/10.1063/1.3463161.

(7) Yasuda, N.; Murayama, H.; Fukuyama, Y.; Kim, J.; Kimura, S.; Toriumi, K.; Tanaka, Y.; Moritomo, Y.; Kuroiwa, Y.; Kato, K.; Tanaka, H.; Takata, M. X-Ray Diffractometry for the Structure Determination of a Submicrometre Single Powder Grain. *J. Synchrotron Radiat.* **2009**, *16* (3), 352–357. https://doi.org/10.1107/S090904950900675X.

(8) Clark, S. J.; Segall, M. D.; Pickard, C. J.; Hasnip, P. J.; Probert, M. I. J.; Refson, K.; Payne, M. C. First Principles Methods Using CASTEP. *Z. Für Krist. - Cryst. Mater.* **2005**, *220* (5/6). https://doi.org/10.1524/zkri.220.5.567.65075.

(9) Monkhorst, H. J.; Pack, J. D. Special Points for Brillouin-Zone Integrations. *Phys. Rev. B* **1976**, *13* (12), 5188–5192. https://doi.org/10.1103/PhysRevB.13.5188.

(10) Perdew, J. P.; Burke, K.; Ernzerhof, M. Generalized Gradient Approximation Made Simple. *Phys. Rev. Lett.* **1996**, *77* (18), 3865–3868. https://doi.org/10.1103/PhysRevLett.77.3865.

(11) Tkatchenko, A.; Scheffler, M. Accurate Molecular Van Der Waals Interactions from Ground-State Electron Density and Free-Atom Reference Data. *Phys. Rev. Lett.* **2009**, *102* (7), 073005. https://doi.org/10.1103/PhysRevLett.102.073005.

(12) Refson, K.; Tulip, P. R.; Clark, S. J. Variational Density-Functional Perturbation Theory for Dielectrics and Lattice Dynamics. *Phys. Rev. B* **2006**, *73* (15), 155114. https://doi.org/10.1103/PhysRevB.73.155114.

(13) Giannozzi, P.; Baroni, S.; Bonini, N.; Calandra, M.; Car, R.; Cavazzoni, C.; Ceresoli, D.; Chiarotti, G. L.; Cococcioni, M.; Dabo, I.; Dal Corso, A.; de Gironcoli, S.; Fabris, S.; Fratesi, G.; Gebauer, R.; Gerstmann, U.; Gougoussis, C.; Kokalj, A.; Lazzeri, M.; Martin-Samos, L.; Marzari, N.; Mauri, F.; Mazzarello, R.; Paolini, S.; Pasquarello, A.; Paulatto, L.; Sbraccia, C.; Scandolo, S.; Sclauzero, G.; Seitsonen, A. P.; Smogunov, A.; Umari, P.; Wentzcovitch, R. M. QUANTUM ESPRESSO: A Modular and Open-Source Software Project for Quantum Simulations of Materials. *J. Phys. Condens. Matter* **2009**, *21* (39), 395502. https://doi.org/10.1088/0953-8984/21/39/395502.

(14) Bloechl, P. E. Projector Augmented-Wave Method. *Phys. Rev. B* **1994**, *50* (24), 953–979.

(15) Maintz, S.; Deringer, V. L.; Tchougréeff, A. L.; Dronskowski, R. LOBSTER: A Tool to Extract Chemical Bonding from Plane-Wave Based DFT. *J. Comput. Chem.* **2016**, *37* (11), 1030–1035. https://doi.org/10.1002/jcc.24353.

(16) Deringer, V. L.; Tchougréeff, A. L.; Dronskowski, R. Crystal Orbital Hamilton Population (COHP) Analysis As Projected from Plane-Wave Basis Sets. *J. Phys. Chem. A* **2011**, *115* (21), 5461–5466. https://doi.org/10.1021/jp202489s.
(17) Dronskowski, R.; Bloechl, P. E. Crystal Orbital Hamilton Populations (COHP): Energy-Resolved Visualization of Chemical Bonding in Solids Based on Density-Functional Calculations. *J. Phys. Chem.* **1993**, *97* (33), 8617–8624. https://doi.org/10.1021/j100135a014.
(18) Bhattacharya, B.; Michalchuk, A. A. L.; Silbernagl, D.; Rautenberg, M.; Schmid, T.; Feiler, T.; Reimann, K.; Ghalgaoui, A.; Sturm, H.; Paulus, B.; Emmerling, F. A Mechanistic Perspective on Plastically Flexible Coordination Polymers. *Angew. Chem. Int. Ed.* **2020**, *59* (14), 5557–5561. https://doi.org/10.1002/anie.201914798.
(19) Birch, F. Finite Elastic Strain of Cubic Crystals. *Phys. Rev.* **1947**, *71* (11), 809–824. https://doi.org/10.1103/PhysRev.71.809.
(20) Vinet, P.; Rose, J. H.; Ferrante, J.; Smith, J. R. Universal Features of the Equation of State of Solids. *J. Phys. Condens. Matter* **1989**, *1* (11), 1941–1963. https://doi.org/10.1088/0953-8984/1/11/002.
(21) Cliffe, M. J.; Goodwin, A. L. *PASCal* : A Principal Axis Strain Calculator for Thermal Expansion and Compressibility Determination. *J. Appl. Crystallogr.* **2012**, *45* (6), 1321–1329. https://doi.org/10.1107/S0021889812043026.
